# *Neurospora intermedia* from a traditional fermented food enables waste-to-food conversion

**DOI:** 10.1038/s41564-024-01799-3

**Published:** 2024-08-29

**Authors:** Vayu Maini Rekdal, José Manuel Villalobos-Escobedo, Nabila Rodriguez-Valeron, Mikel Olaizola Garcia, Diego Prado Vásquez, Alexander Rosales, Pia M. Sörensen, Edward E. K. Baidoo, Ana Calheiros de Carvalho, Robert Riley, Anna Lipzen, Guifen He, Mi Yan, Sajeet Haridas, Christopher Daum, Yuko Yoshinaga, Vivian Ng, Igor V. Grigoriev, Rasmus Munk, Christofora Hanny Wijaya, Lilis Nuraida, Isty Damayanti, Pablo Cruz-Morales, Jay. D. Keasling

**Affiliations:** 1 Department of Bioengineering, University of California Berkeley, Berkeley, CA USA; 2 Miller Institute for Basic Research in Science, University of California Berkeley, Berkeley, CA USA; 3 Joint BioEnergy Institute, Emeryville, CA USA; 4 Department of Plant and Microbial Biology, University of California Berkeley, Berkeley, CA USA; 5 Environmental Genomics and Systems Biology Division, Lawrence Berkeley National Laboratory, Berkeley, CA USA; 6 Tecnológico de Monterrey, Institute for Obesity Research, Monterrey, Nuevo León Mexico; 7 ALCHEMIST Explore, Research and Development, Alchemist Aps, Copenhagen, Denmark; 8 Basque Culinary Center, Mondragon Universitatea, Donostia, Gipuzkoa Spain; 9 Department of Chemical and Biomolecular Engineering, University of California Berkeley, Berkeley, CA USA; 10 Harvard John A. Paulson School of Engineering and Applied Sciences, Harvard University, Cambridge, MA USA; 11 Biological Systems and Engineering Division, Lawrence Berkeley National Laboratory, Berkeley, CA USA; 12 Novo Nordisk Foundation Center for Biosustainability, Technical University of Denmark, Kgs. Lyngby, Denmark; 13 US Department of Energy Joint Genome Institute, Lawrence Berkeley National Laboratory, Berkeley, CA USA; 14 Department of Food Science and Technology, Faculty of Agricultural Engineering, IPB University (Bogor Agricultural University), Dramaga, Indonesia; 15 California Institute of Quantitative Biosciences (QB3), University of California Berkeley, Berkeley, CA USA

**Keywords:** Systems biology, Fungal evolution, Industrial microbiology, Fungal genomics

## Abstract

Fungal fermentation of food and agricultural by-products holds promise for improving food sustainability and security. However, the molecular basis of fungal waste-to-food upcycling remains poorly understood. Here we use a multi-omics approach to characterize oncom, a fermented food traditionally produced from soymilk by-products in Java, Indonesia. Metagenomic sequencing of samples from small-scale producers in Western Java indicated that the fungus *Neurospora intermedia* dominates oncom. Further transcriptomic, metabolomic and phylogenomic analysis revealed that oncom-derived *N. intermedia* utilizes pectin and cellulose degradation during fermentation and belongs to a genetically distinct subpopulation associated with human-generated by-products. Finally, we found that *N. intermedia* grew on diverse by-products such as fruit and vegetable pomace and plant-based milk waste, did not encode mycotoxins, and could create foods that were positively perceived by consumers outside Indonesia. These results showcase the traditional significance and future potential of fungal fermentation for creating delicious and nutritious foods from readily available by-products.

## Abstract (web summary)

A multi-omics analysis of oncom, an Indonesian fermented food made from soymilk waste, shows how associated fungi break down food waste to yield nutritious and positively received foods.

## Main

Minimizing food waste is important for improving the resiliency and sustainability of the food system^1–3^. In industrialized countries such as the United States, approximately a third of food is wasted, and loss and waste of food accounts for approximately half of total greenhouse gas emissions caused by the food system on a global level^4,5^. Upgrading waste into value-added products (a process termed upcycling) can reduce the climate impact of food production while also enhancing food security and promoting financial benefits^6,7^. In particular, microbial processes for upcycling have shown promise for converting otherwise wasted substrates into sustainable protein^8^ and new foods^9^.

Filamentous fungi, a diverse group of microorganisms that includes moulds and mushrooms, are ideally suited for microbial upcycling of waste streams (by-products) generated in the transformation of crops into foods. For instance, growing fungi on by-products in large-scale liquid fermentations for heterologous production of animal proteins or for edible biomass can offset global greenhouse gas emissions and land use changes caused by resource-intensive animal agriculture^8,10^. Similarly, solid-state fermentation (SSF), a process in which fungi are directly grown on solid substrates rather than in submerged tanks, can reduce global land pressure and greenhouse gas emissions while promoting food system circularity, particularly when used to produce human food^1,2,11^. In contrast to large-scale liquid fermentations, SSF is an accessible technology that requires minimal equipment and inputs. The low technological barrier, alongside the local availability of many by-products, makes SSF ideally suited as a decentralized solution to improve food security and nutrition for vulnerable populations^12–15^.

Despite the promises of SSF for transforming by-products into human foods with improved nutrition and sensory appeal^15,16^, engineering by-product–strain combinations in SSF for desired outcomes remains challenging. So far, a majority of scientific and gastronomic investigations of by-product SSF for human food have been conducted on a trial-and-error basis with a poor understanding of the underlying molecular mechanisms^9,15–18^. Characterizing fungal waste-to-food conversion in molecular detail is important for expanding the engineering possibilities with SSF and enabling design of fermentations for specific organoleptic properties and nutritional profiles.

**Fig. 1:**
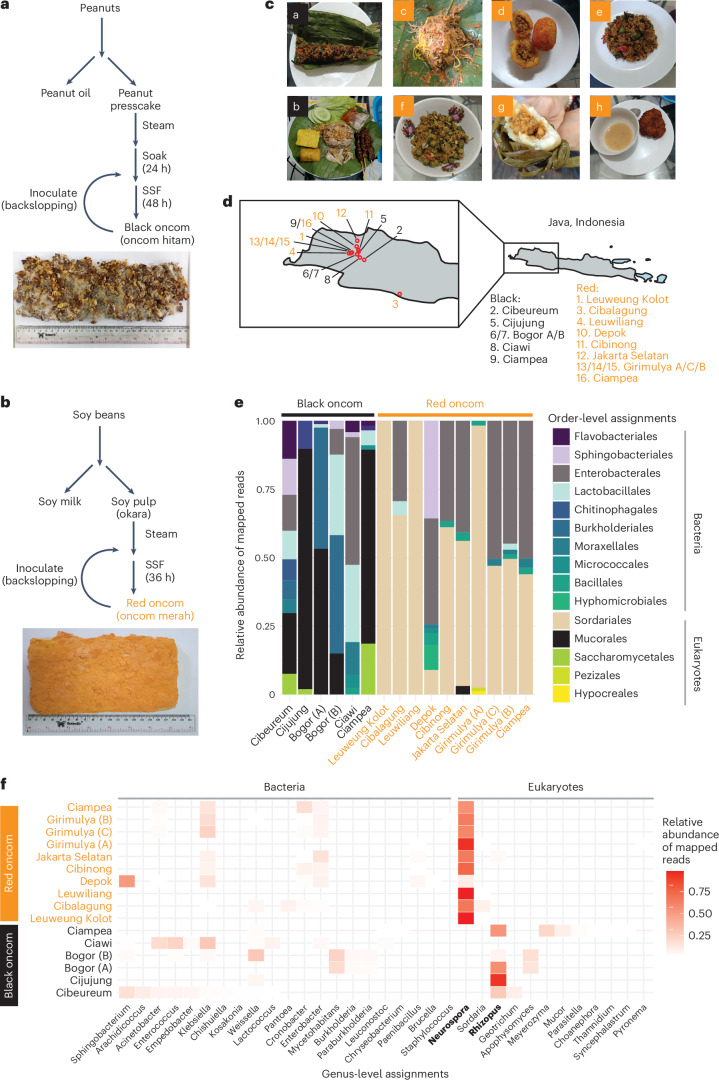
Metagenomic survey of black and red oncom collected from Java, Indonesia. **a**, Traditional production method for black oncom. A typical sample of black oncom is shown. **b**, Traditional production method for red oncom. A typical sample of red oncom is shown. **c**, Typical preparations of oncom in West Java, Indonesia. a, ‘pepes oncom’ (black oncom cooked in a banana leaf with spices); b, ‘nasi tutug oncom’ (black oncom cooked in rice with spices and served with side dishes and condiments); c, ‘toge goreng’ (red oncom forms the basis of the spicy sauce used to season vegetables, other ingredients); d, ‘combro’ (red oncom is the main ingredient in stuffing); e, ‘krecek oncom’ (red oncom stir fried with vegetables, spices); f, ‘oncom leunca’ (red oncom cooked with leunca, a nightshade vegetable); g, ‘buras oncom’ (red oncom is the main filling of the pouch steamed in banana leaf); h, ‘perkedel oncom’ (fried cakes made from red oncom, spices and other ingredients). **d**, Production sites of oncom samples analysed by metagenome sequencing in this study. The island of Java is shown in grey, and the enlarged picture shows specific production locations. Numbers correspond to locations where samples were collected. Letters indicate that more than one producer was sampled from a specific geographical location. Precise coordinates are found in Supplementary Table 1. **e**, Order-level taxonomy assignments based on metagenome sequencing data. *Neurospora* belongs to Sordariales, while *Rhizopus* belongs to Mucorales. Relative abundances of mapped, non-viral reads (>1% abundance) are shown. **f**, Genus-level abundance of the same samples shown in **e**. Relative abundances of mapped, non-viral reads (>1% abundance) are shown. *Neurospora* and *Rhizopus*, the fungal organisms traditionally associated with red and black oncom, respectively, are highlighted in bold. Source data

Here we use multi-omics analyses to characterize oncom, a fermented food traditionally produced through SSF of by-products on the island of Java, Indonesia. There are two types of oncom. Whereas the red type (oncom merah) is produced from soymilk by-products (okara), the black type (oncom hitam) is made from the oil presscake left over from peanut oil production^19^ (Fig. 1a,b). Oncom fermentation is initiated by backslopping from a previous batch, followed by a 36–48 h fermentation period that transforms the by-product into a rectangular block that is readily used as a meat substitute and serves diverse culinary roles in the local cuisine (Fig. 1a–c)^20^. While red and black oncom fermentations are assumed to be driven by fungi of the *Neurospora* and *Rhizopus* genera, respectively^13,21–23^, details of the microbial, biochemical and genetic dynamics underlying the fermentation process are lacking.

## Results

### Metagenomic survey of oncom across Indonesian producers

**Extended Data Fig. 1:**
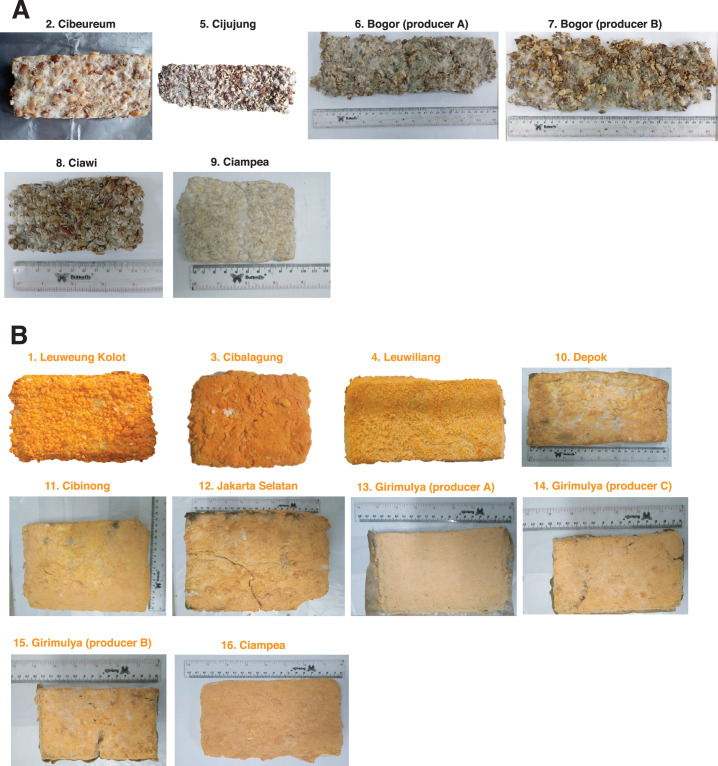
Photos of black and red oncom samples collected from different producers in Java, Indonesia. The numbers refer to the locations shown in Supplementary Table 1 and in Fig. 1 in the main text. All these samples were subjected to metagenome sequencing for taxonomic profiling. Three red (**b**) and two black (**a**) oncom samples were also subjected to amplicon sequencing.

Although *Neurospora* and *Rhizopus* have been isolated from red and black oncom, respectively, and are traditionally considered the major organisms driving fermentation^13,23^, no culture-independent method has been deployed to characterize oncom samples across different producers, painting an incomplete picture of the underlying microbial dynamics. We first collected red oncom (*n* = 10) and black oncom (*n* = 6) samples from artisanal producers in Western Java and used shotgun metagenome sequencing to profile microbial composition of the samples, assigning taxonomic identities to the short reads using kaiju^24,25^ (Fig. 1d, Extended Data Fig. 1 and Supplementary Table 1).

This metagenomic survey revealed striking differences in microbial community composition across oncom type and producer origin. Whereas black oncom samples displayed notable microbial variability between producers (Fig. 1e,f and Supplementary Note 1), *Neurospora* was the main fungus detected across all red oncom samples, and its abundance ranged from complete dominance (>95% of all mapped reads) to co-existence with bacteria and even low levels of other fungi (<9%) in some samples (Fig. 1e,f). In all red oncom samples but the one collected from Depok, in which a majority of reads were assigned to the Gram-negative bacterium *Sphingobacter*, *Neurospora* was the most abundant single microorganism, making up >50% of all classified reads (Fig. 1f). In the samples that were dominated by *Neurospora* and harboured bacteria, the main bacterial reads were assigned to Proteobacteria such as *Klebsiella, Enterobacter* and *Cronobacter*. These bacteria were also found in some black oncom samples (Fig. 1e,f). Such organisms are not typically considered major players in the production of fermented foods and it remains unclear from our data whether these bacteria are actively participating in fermentation or are contaminants and spoilage organisms that appear post production, which is the major context in which Proteobacteria are frequently observed in human food production^26,27^. The microbial difference between the two oncom types was further supported by 16S and internal transcribed spacer (ITS) amplicon sequencing (Supplementary Figs. 1 and 2).

**Extended Data Fig. 2:**
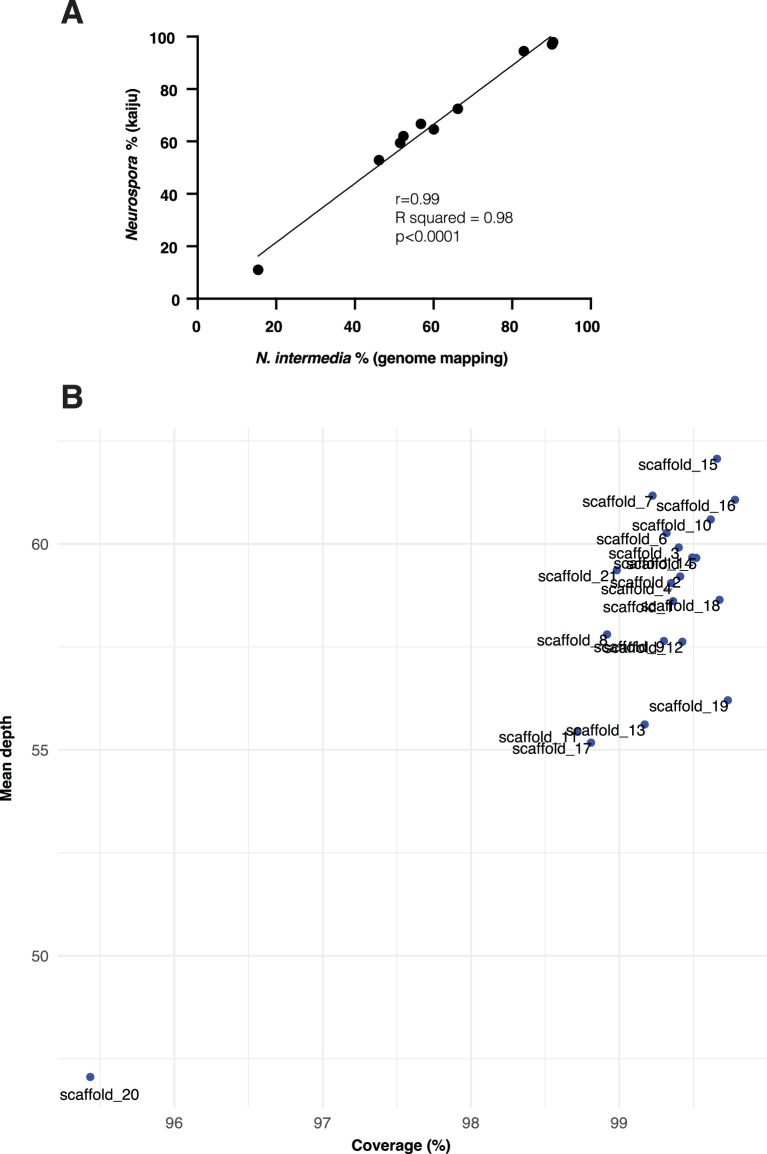
Mapping of shotgun metagenome data to *N. intermedia* genome. **A**) Correlation between % reads assigned to the *Neurospora* genus using the kaiju database, which does not contain a genome for *Neurospora intermedia*, and the % of reads mapped to our sequenced *N. intermedia* FGSC #2613 genome using this as the only query (<2 mismatches allowed). There was a strong correlation (Correlation test, r = 0.99, R squared = 0.98, p < 0.0001, n-10 pairs) between the % abundance by these two methods, indicating that a majority of the % reads assigned to *Neurospora* by kaiju also readily map to the sequenced *N. intermedia* FGSC #2613 genome. **B**) Coverage of the *N. intermedia* FGSC #2613 genome by scaffold. The shotgun metagenome sequences from all samples were combined and mapped against the *N. intermedia* genome. The results indicate >98.5% coverage for all scaffolds but scaffold 20 (mean coverage=99%). Source data

*Neurospora intermedia* has historically been the main fungus isolated from red oncom samples^13,22,23^. While our data displayed a consistent abundance of *Neurospora* in red oncom, the standard kaiju taxonomy database does not contain an *N. intermedia* genome, making it difficult to validate these previous culture-based observations. To enable greater metagenomic species-level resolution, we first generated a genome of the oncom-derived reference strain *N. intermedia* FGSC #2613 using long-read sequencing and annotating the genes using transcriptomics. The 39 Mb genome has a GC content of 49%, was spread across 21 contigs instead of >1,100 and had a 60-fold higher N50 (the shortest contig length that needs to be included for covering 50% of the genome), reflecting the superior genome contiguity and quality compared with the previous best *N. intermedia* genome^28^ (Supplementary Table 2). Using this genome to map the metagenomic reads, we found a strong and significant correlation between the metagenomic abundance of *Neurospora* and the percent of reads mapping to *N. intermedia* (Extended Data Fig. 2a). In addition, the shotgun metagenome data covered 99% of the *N. intermedia* genome (Extended Data Fig. 2b). These results reinforce that *N. intermedia* is the major fungal species in red oncom. The abundance and prevalence of *N. intermedia* across samples made us wonder whether this fungus harboured specific genetic and biochemical features making it uniquely suitable for waste-to-food upcycling. Thus, we focused our further characterization efforts on red oncom.

### Biochemical changes during *N. intermedia* fermentation

**Extended Data Fig. 3:**
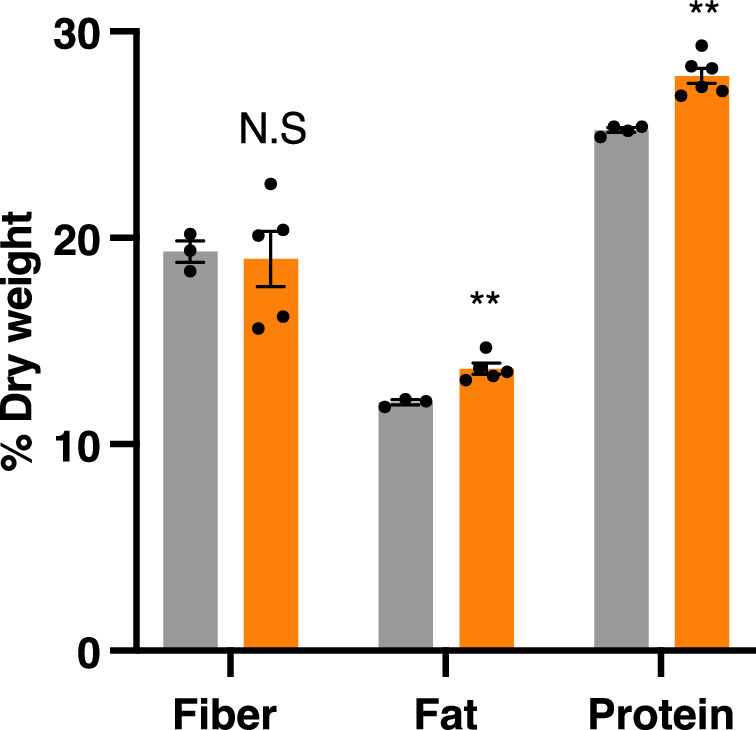
Nutrient profile of raw and fermented okara. While the fiber did not change during the fermentation, both protein (**=p = 0.0065, unpaired two-sided t-test) and lipid content (**=p = 0.0054, unpaired t-test) significantly increased, from 25% to 28% and from 12–14%, respectively (dry weight basis). For protein, results are mean and SEM from n = 6 biological for fermented and n = 4 biological replicates for unfermented samples. For lipids and fiber, results are mean and SEM from n = 3 biological for fermented and n = 5 biological replicates for unfermented samples. Source data

**Extended Data Fig. 4:**
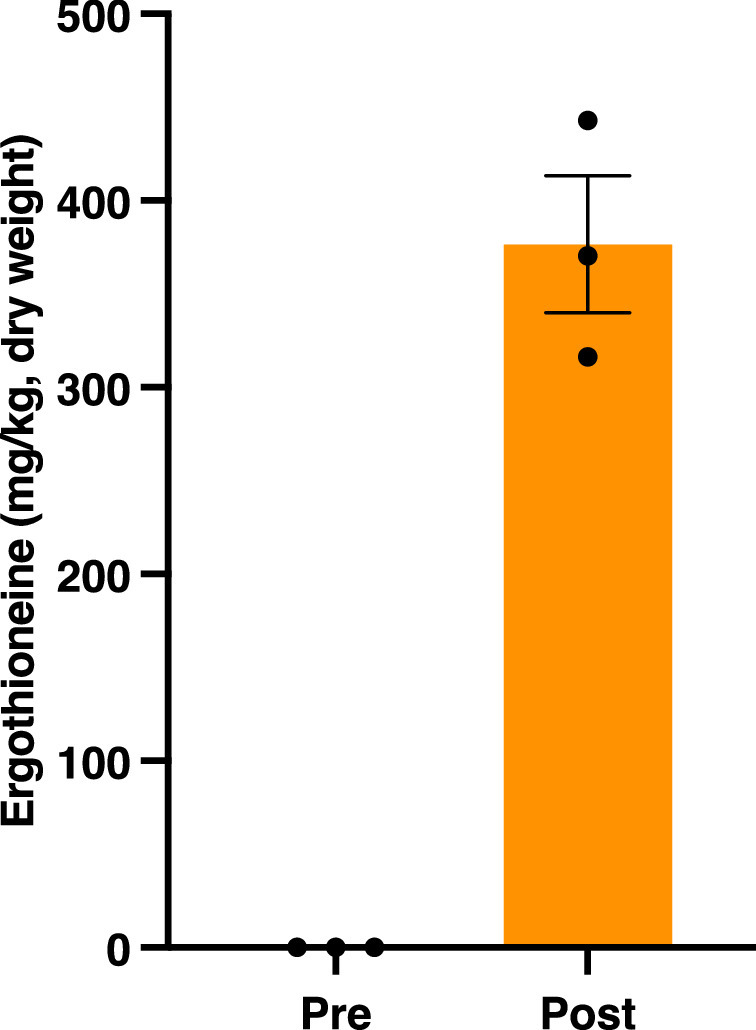
Ergothioneine levels in raw and fermented okara. Okara samples were fermented with *N. intermedia* for 48 hours in solid-state. The fermentation produced ergothioneine, a potent antioxidant that is associated with several health benefits. Ergothioneine was absent in the starting material, suggesting active fungal biosynthesis during the fermentation process. Results are mean and SEM on a dry weight basis from n = 3 biological replicates and were analyzed by LC-MS. Source data

We next set out to characterize the red oncom fermentation at the biochemical and genetic levels by cultivating *N. intermedia* on okara. SSF of okara increased the protein content (from 25% to 28%, *P* < 0.001, unpaired *t*-test) and lipid content (from 12% to 14%, *P* < 0.01, unpaired *t*-test) of okara but did not change crude fibre (Extended Data Fig. 3). Amino acid composition did not change majorly, and fermented okara retained all essential amino acids contained in the starting material (Supplementary Fig. 3a). In contrast, SSF released free amino acids, most notably high levels of glutamine as well as glutamate, an amino acid that contributes to the umami taste in oncom^19^ (Supplementary Fig. 3b,c). We also detected changes in the volatile aroma composition of okara upon *N. intermedia* fermentation, including a 40-fold decrease in the off-flavour hexanal to barely detectable levels^29^ (Supplementary Table 3). SSF also produced high levels of ergothioneine, a potent antioxidant that has been associated with several health benefits in humans^30,31^. Absent in okara, post-fermentation ergothioneine levels reached ~25% of those found in oyster mushrooms, the dietary source containing the highest levels^30,32^ (Extended Data Fig. 4). These results suggest that *N. intermedia* fermentation modulates the biochemical composition of okara in ways that potentially make it more palatable and functionally beneficial to humans.

**Fig. 2:**
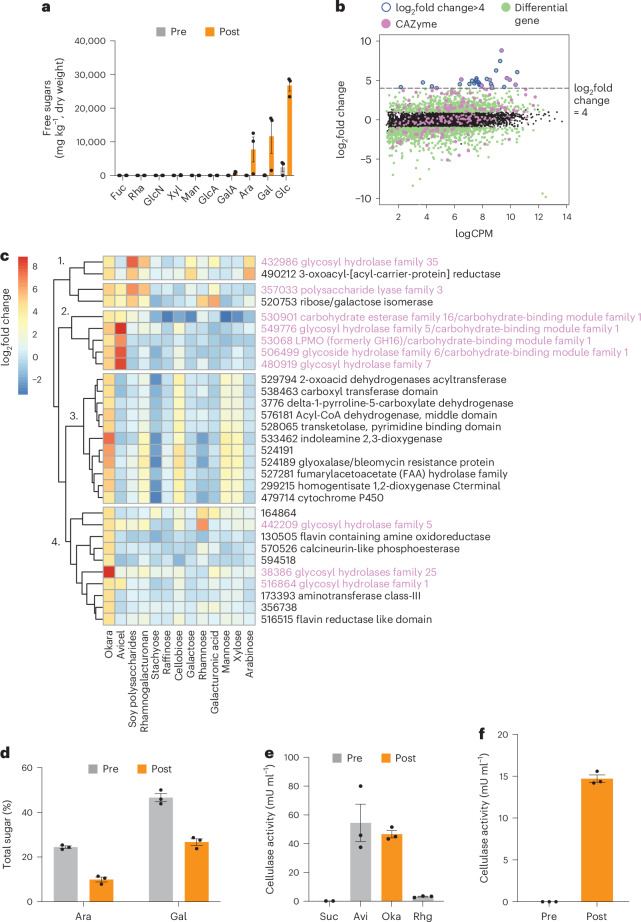
Transcriptomics identifies cellulose and pectin degradation during okara breakdown by *N. intermedia.* **a**, Free sugars detected in okara subjected to SSF by *N. intermedia* before and after the fermentation. Fuc, fucose; Rha, rhamnose; GlcN, *N*-acetylglucosamine; Xyl, xylose; Man, mannose; GlcA, glucuronic acid; GalA, galacturonic acid; Ara, arabinose; Gal, galactose; Glc, glucose. Results are mean ± s.e.m. across 3 biological replicates. **b**, *N. intermedia* gene expression profile in response to okara. Results are shown as the log_2_FC compared to a no-carbon control. Green indicates significantly differentially expressed genes. Purple indicates a predicted CAZyme. The blue border around the circle indicates a gene significantly induced above the log_2_FC threshold of 4, which captured 10 CAZymes and 21 genes with other predicted functions. **c**, Expression of the genes that were most highly induced in okara (log_2_FC > 4) across purified carbon sources of decreasing complexity. As in **b**, purple indicates a predicted CAZyme. The substrates were chosen on the basis of their presence in soybeans or okara polymers. Clustering of the data revealed the existence of four separate clusters displaying distinct expression patterns. **d**, Sugar analysis of the hydrolysed pectin-containing fraction in okara samples subjected to SSF by *N. intermedia*. Results are mean ± s.e.m. on a dry weight basis from 3 biological replicates. **e**, Cellulase activity across different carbon sources in liquid cultures. Oka, okara; Av, avicel; Suc, sucrose; Rhg, rhamnogalacturonan. Results are mean ± s.e.m. of 3 biological replicates. **f**, Cellulase activity detected in okara subjected to SSF by *N. intermedia*. Results are mean ± s.e.m. of 3 biological replicates. Source data

We next sought to identify the genes and enzymes supporting the growth of *N. intermedia* on okara during the fermentation process. As the by-product of extraction of soluble protein and sugars into soymilk, okara is largely composed of insoluble polysaccharides originating from the plant cell wall, including pectin and cellulose^33,34^. Upon further characterization of *Neurospora-*fermented okara, we noted the release of free sugars, including arabinose, galactose and glucose, which were undetectable or found at low levels in the starting material (Fig. 2a). As these free sugars could be derived from breakdown of complex polysaccharides, these results strongly suggested activity of carbohydrate active enzymes (CAZymes), a group of enzymes that are associated with degradation of complex polysaccharides and support fungal growth on plant biomass^35^. We used transcriptomics to identify potential CAZymes involved in okara degradation by *N. intermedia*.

Okara dramatically changed the global gene expression profile, including significantly upregulating several predicted CAZymes (Fig. 2b and Supplementary Table 4). Above a cut-off of log_2_(fold change (FC)) > 4, which we reasoned would capture the most highly expressed genes in response to okara^35^, 10 of these CAZymes remained, in addition to 21 other genes with diverse predicted functions (Fig. 2b and Supplementary Table 5). However, assigning these genes to specific metabolic pathways or substrates within the complex okara biomass was challenging using computational prediction alone, so we repeated the experiment and instead exposed the strain to purified mono-, di- and polysaccharide components of okara as the sole carbon source (*n* = 12 unique substrates) and then mapped the top genes overexpressed on okara to the transcriptomics data from the purified substrates.

**Extended Data Fig. 5:**
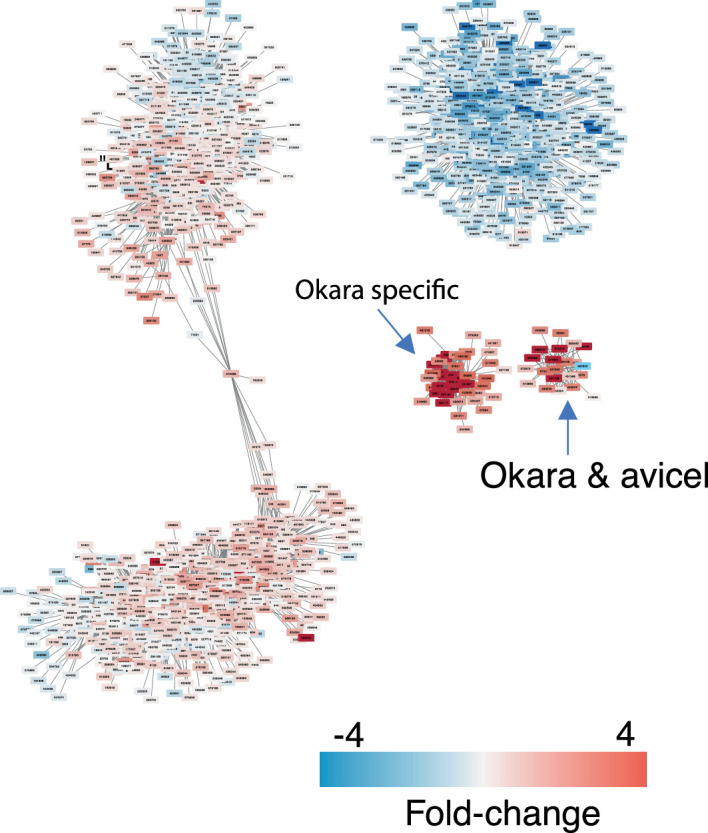
Global co-expression analysis reveals a shared transcriptional module between okara and avicel as well as genes uniquely expressed on okara. The figure shows the global co-expression network among all genes and conditions, which revealed a shared transcriptional module between okara and avicel, as well as a transcriptional module that was unique to okara. In the network, the values of log2 Fold-change are observed in the comparison okara vs no carbon control (see Supplementary table 4). Source data

Initial heat map analysis using hierarchical clustering revealed a diversity of genes that fell into four distinct groups and were either unique to okara or shared with the purified substrates (Fig. 2c). While the gene expression profile in group 1 suggested pectin degradation and potential uptake of released sugars, group 2 included several genes that were shared between okara and Avicel (purified cellulose), indicating active cellulose degradation. Groups 3 and 4 included glycosyl hydrolases, genes involved in amino acid metabolism, predicted proteins with poorly understood functions, or hypothetical proteins. Co-expression analysis across all conditions confirmed a shared transcriptional module between okara and Avicel, further reinforcing the presence of active cellulose degradation during okara fermentation (Extended Data Fig. 5).

**Extended Data Fig. 6:**
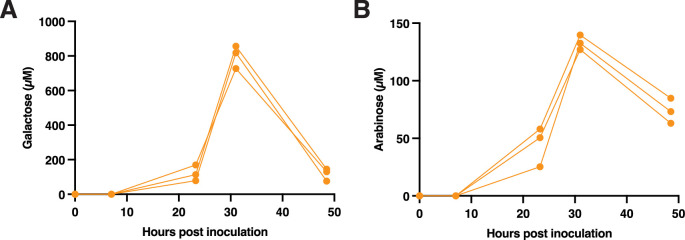
Profiling of arabinose and galactose during *N. intermedia* growth on okara as the sole carbon source in liquid cultures. Results display three biological replicates. Supernatants of VMM medium liquid cultures harboring okara as the sole carbon source (1 % w/v) were analyzed for secreted galactose (**A**) and arabinose (**B**) by HPAEC. Analysis of okara SSF had suggested both of these sugars are released during the fermentation (Fig. 2 in main text) and are also depleted from the pectin fraction, suggesting active release from pectin and subsequent fungal consumption. The graph above supports this conclusion as it reveals initial release of these sugars from the okara substrate, followed by disappearance from the culture medium, suggesting active fungal hydrolysis and subsequent uptake. Source data

To biochemically validate these transcriptomics observations, we focused on pectin and cellulose degradation, as these are relevant to upcycling of diverse plant-based by-products for human consumption. Analysis of the sugar composition in the hydrolysed pectin fraction of okara revealed a clear decrease in both arabinose (60% decrease) and galactose (40% decrease), suggesting release of pectin-bound sugars (Fig. 2d). Liquid cultures using okara as the only carbon source confirmed that arabinose and galactose were initially released to the medium and then taken up by the fungus, with only a small amount remaining, suggesting direct consumption during the fermentation (Extended Data Fig. 6). These two sugars are abundant in rhamnogalacturonan, the main pectic fibre in soy, and the position of these sugars within the side chain of the polymer makes them accessible for hydrolytic release and microbial degradation, which is consistent with our data^33,36^. We did not detect a clear decrease of galacturonic acid in SSF, which is found in more inaccessible portions of the pectin polymer^36^, or in any other sugar (Supplementary Fig. 4). Similarly, consistent with the transcriptomics data, we detected active cellulose degradation (as assessed by cellulase enzyme activity) during growth on Avicel and okara as the sole carbon source, but not on sucrose or rhamnogalacturonan, which were included as negative controls (Fig. 2e). We also detected cellulase activity in okara fermented by *N. intermedia* through SSF, and this activity was absent in the starting material, suggesting active fungal secretion during SSF (Fig. 2f). These data establish degradation of both pectin and cellulose, two polymers that are abundant in many plant-based by-products, during *N. intermedia* growth on okara. While cellulose degradation may explain the release of free glucose, pectin metabolism could account for the release of both arabinose and galactose during SSF (Fig. 2a).

### Oncom-associated *N. intermedia* is genetically distinct

**Fig. 3:**
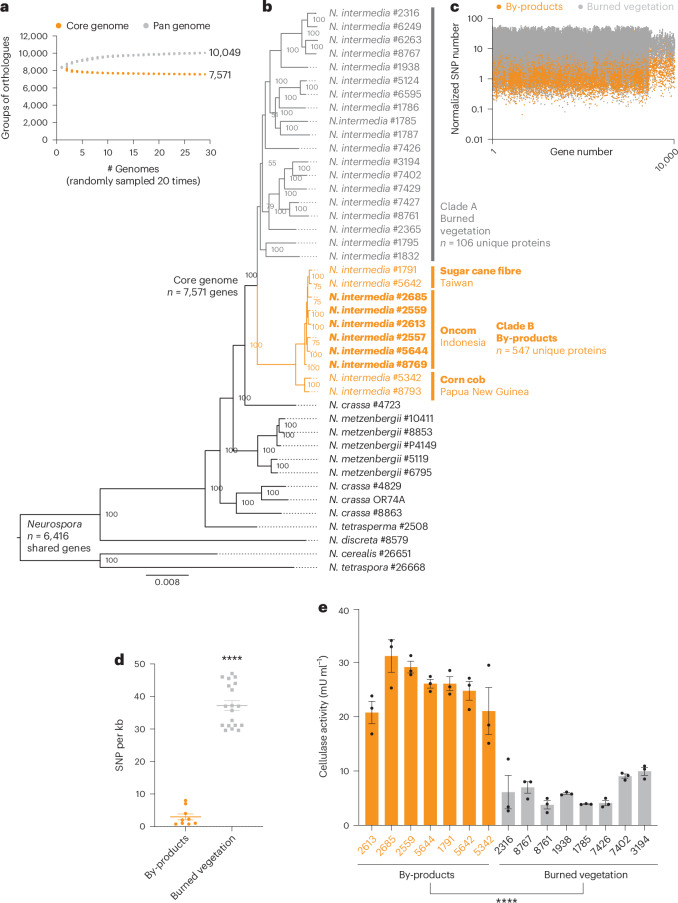
*N. intermedia* strains used for oncom belong to a genetically distinct subpopulation associated with by-products of human activity. **a**, Core/Pan genome analysis for *N. intermedia*. Grey and orange circles show the number of protein families in the pan and core genomes for a subset of *n* strains (*x* axis). This calculation was done 20 times for a random sample of *n* genomes. The number of core and unique genes across all strains are shown alongside the curves. **b**, Phylogenetic tree of *Neurospora* species based on shared coding genes (*n* = 6,416). Clade A was associated with burned vegetation, a common site for isolating *Neurospora* species. Clade B included all oncom-derived strains (highlighted in bold) collected from Indonesia, as well as strains isolated from sugar cane bagasse (#1791, #5642), the fibrous by-product of sugar production, or leftover corn cobs (#5342, #8793). Oncom-derived strain #2559 and burned strain #8767 were both isolated from the town of Bogor on the island of Java, Indonesia, reinforcing a genetic rather than geographical distinction between the two clades. **c**, Analysis of SNPs across all genes in *N. intermedia* strains. SNPs were fixed to the reference strain #2613 and mapped across all shared genes among the remaining 28 strains. Results were normalized to gene length. **d**, The number of SNPs per kb in the predicted cellulase (#480919, glycosyl hydrolase family 7) that was highly expressed on both okara and Avicel. Mean ± s.e.m. are shown, with individual data points representing the value from each strain (****P < 0.0001, unpaired two-sided *t*-test). **e**, Secreted cellulase activity between burn-associated and by-product-associated strains during growth on okara. Results are mean ± s.e.m. across 3 biological replicates. There was a 4.1-fold lower mean secreted cellulase activity among burn-associated strains compared with by-product-associated strains (****P < 0.0001, unpaired two-sided *t*-test). Source data

**Extended Data Fig. 7:**
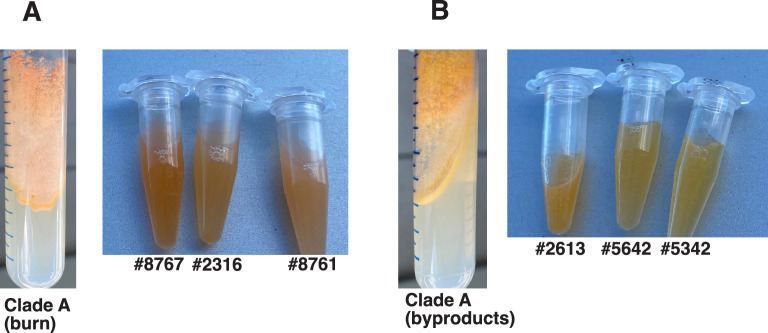
Representatives from the two clades of *Neurospora intermedia* have previously reported differences in conidial pigmentation. Prior to our work, two types of phenotypically distinct *N. intermedia* strains had been described, which differed in conidial size and pigmentation. We found direct evidence of two genetically distinct clades of *N. intermedia*, which associated with different ecological origins (Fig. 3 in main text). Phenotypic analysis confirmed that these two clades had the previously reported differences in conidial pigmentation, suggesting that the spore coloration is consistent with underlying genetic differences between the two subpopulations. **A**) A representative strain (#2316) from clade A (burn-associated) growing on a slant indicates an orange pigmentation, which is also clearly visible in conidial suspensions of representative members. **B**) In contrast, byproduct-associated strains have a more yellow-orange coloration, as can be seen during growth on a slant as well as in conidial suspensions of representative strains.

Many fungi traditionally used for food production display genomic adaptations to the substrate on which they are typically grown^37–40^, leading us to wonder about the origins and evolutionary history of *N. intermedia* strains used for oncom. Whole-genome phylogenetic analysis of 29 *N. intermedia* genomes generated between this study and an unrelated study^28^ revealed that *N. intermedia* strains were split into two distinct, closely related groups that segregated on the basis of ecological but not on geographical origin (Fig. 3a,b and Supplementary Table 6). Clade A harboured strains isolated from burned vegetation, while Clade B harboured all sequenced oncom strains (*n* = 6), as well as strains isolated from fibre-rich, plant-based by-products, including sugar cane bagasse, the by-product of sugar production, and leftover corn cobs. Notably, strains from clades A and B displayed differences in conidial pigmentation that have been described for *N. intermedia* strains collected from nature^23^ (Extended Data Fig. 7). These data showcase that *N. intermedia* exists as two genetically distinct populations and that oncom strains are closely related to strains associated with by-products of human activity.

Once we established phylogenetic relationships among *N. intermedia* strains, we used comparative genomics analyses to search for genomic features associated with the by-product-associated lifestyle. The pan-genome of the by-product-associated strains (*n* = 741 genes) was larger compared with that of burn-associated strains (*n* = 132 genes) and was enriched in a range of predicted metabolic functions, including hydrolase activity (*n* = 24 genes, the most enriched) (Supplementary Fig. 5). However, only a few genes could be predicted, and the genome-wide phylogenetic tree strongly suggested that the observed divergence may arise from differences in shared genes rather than differences in gene content (Fig. 3b). Consistent with this prediction, analysis of genome-wide single nucleotide polymorphisms (SNP) in coding sequences clearly revealed the existence of two populations (Fig. 3c). The genes harbouring the most SNPs per kilobase (kb) (*n* = 209 genes, >2 standard deviations beyond the genome’s average mutation rate) included transcription factors, transporters, an ergothioneine biosynthetic enzyme, a polyketide synthase, a predicted plant toxin, as well as several CAZymes (Supplementary Table 7).

**Extended Data Fig. 8:**
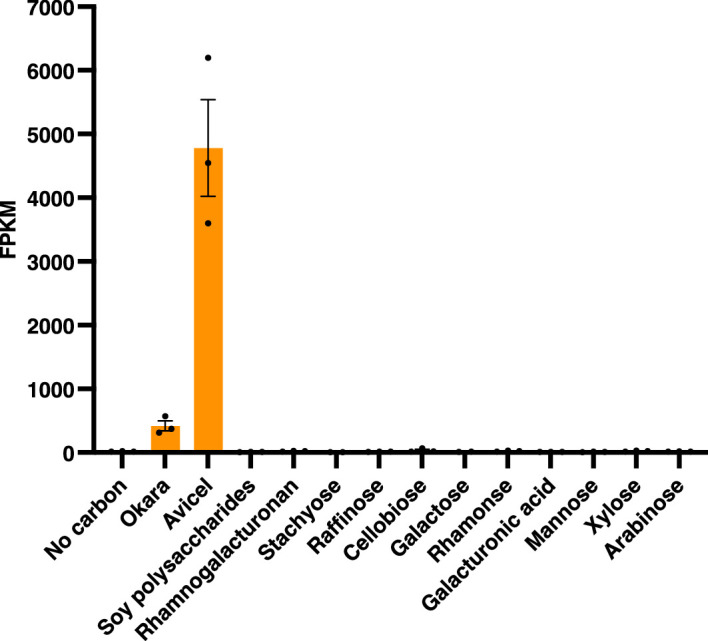
Expression of predicted cellulase belonging to the glycosyl hydrolase 7 family across carbon sources. Expression of the predicted cellulase belonging to the glycosyl hydrolase 7 family (JGI protein ID #480919) that was highly mutated in the burn-associated clade (clade A in Fig. 3 in main text) relative to the byproduct-associated clade (clade B in Fig. 3 in main text). Results are shown as fragments per kilobase million (FPKM) and are mean and SEM of three biological replicates. The gene was highly expressed on okara, and even higher on avicel, the purified cellulose substrate, implicating this gene in cellulose metabolism. Source data

**Extended Data Fig. 9:**
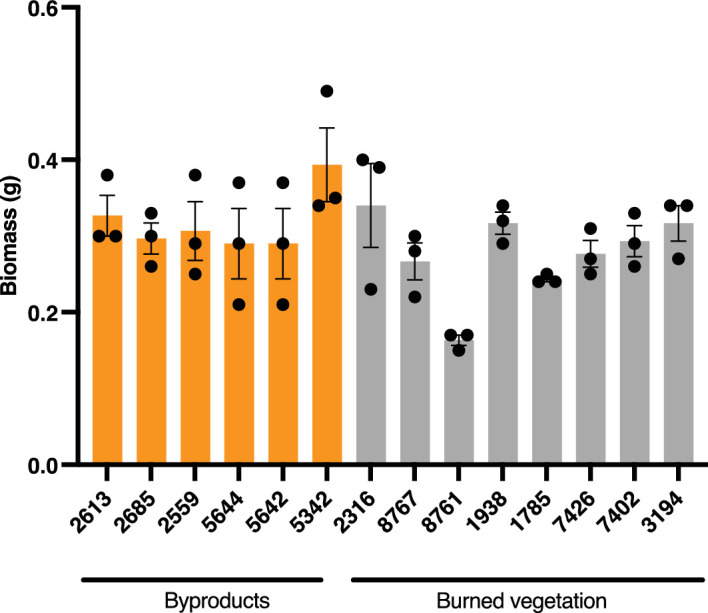
Biomass yield of byproduct-associated and burned-associated strains on okara. Strains were grown in liquid cultures using okara as the sole carbon source. Biomass yield was measured after 72 hours of growth. Results are mean + SEM of three biological replicates. There was no significant difference in mean biomass yield between the two groups. Source data

Cross-referencing the genes harbouring the most SNPs with the full transcriptomics dataset identified a predicted cellulase (glycosyl hydrolase family 7, JGI protein ID #480919 in Fig. 3c and Supplementary Table 5) that was highly expressed on both okara and Avicel (Fig. 2c and Extended Data Fig. 8) and harboured significantly different numbers of SNPs per kb between the two subpopulations (Fig. 3d, *P* < 0.0001, unpaired *t*-test). While the by-product-associated group had an average of <3 SNPs per kb in this cellulase, the burn-associated group harboured 37 coding SNPs, which were mainly located outside the predicted active site (Supplementary Fig. 6). Consistent with this mutation pattern, the secreted cellulase activity was >4-fold higher among by-product-associated strains during growth on okara (Fig. 3e, *P* < 0.0001, unpaired *t*-test). There was no difference in overall biomass yield of the two groups of strains (Extended Data Fig. 9), indicating that the elevated cellulase activity was explained by biochemical rather than growth differences. While we cannot conclude that the increased cellulase activity results from a mutation in the cellulase without further genetic experiments, these results highlight that the two distinct subpopulations of *N. intermedia* display genetic and biochemical differences in metabolic enzymes that are involved in growth on by-products.

### *N. intermedia* grows on diverse plant-based by-products

The observation that the strains used for oncom production belong to a genetically distinct waste-associated subpopulation raised questions about the broader ability of *N. intermedia* to upcycle plant-based by-products beyond okara for applications in non-traditional contexts. To evaluate this possibility, we screened the growth of oncom-derived *N. intermedia* #2613 on a library of *n* = 30 industrially relevant by-product substrates, including plant-based milk by-products, fruit and vegetable pomace, grain and agricultural processing by-products, brewing by-products, oilseed presscakes and others.

**Fig. 4:**
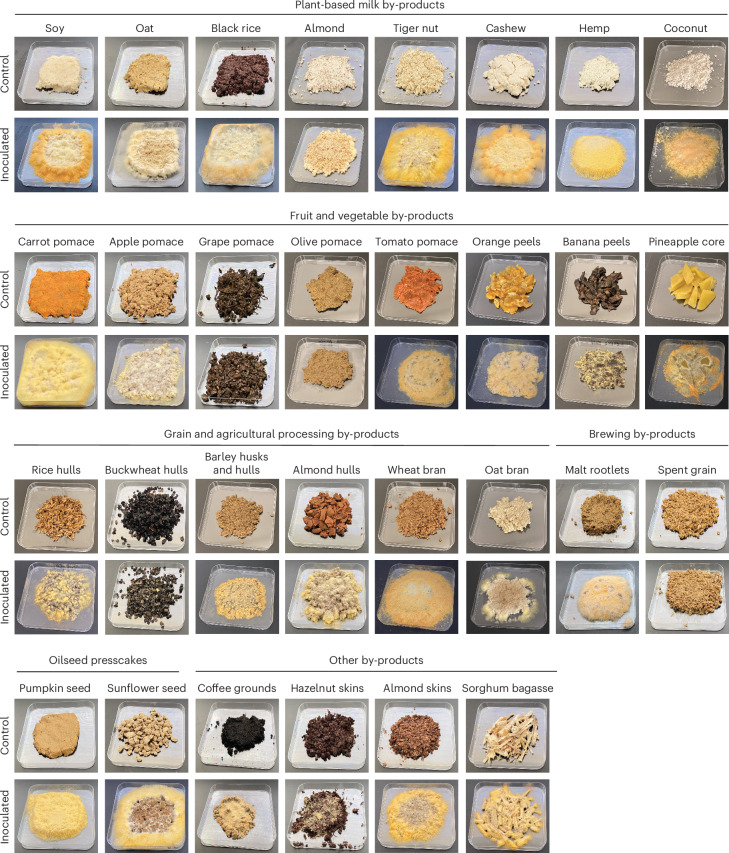
*N. intermedia* grows on diverse industrial by-products from food and agricultural processing. SSF screening of oncom-derived *N. intermedia* #2613 growth across diverse industrially relevant by-products. Okara was included as a positive control and benchmark for fungal growth. *N. intermedia* grew on all substrates tested except for grape pomace, olive pomace and buckwheat hulls, as assessed by the development of mycelia and/or conidia. Source data

Following 72 h of SSF, we found that *N. intermedia* grew on all by-products but grape pomace, olive pomace and buckwheat hulls, as assessed by the development of mycelia and conidia (Fig. 4). However, the extent of growth varied between substrates. There was a moderate amount of growth on coffee grounds, almond hulls, banana peels, pineapple core, oat bran and almond milk by-product, while only minimal growth was observed on hazelnut skins and spent grain (Fig. 4 and Extended Data Table 1). All others supported a high level of growth comparable to that observed on okara. Strikingly, among a subset of samples that supported high *N. intermedia* growth and were selected for further analysis, we found a consistent increase in protein content after SSF (Supplementary Table 8). These results illuminate the broad substrate range of oncom-derived *N. intermedia* and suggest potential opportunities for upcycling diverse by-products into more nutritious foods.

### *N. intermedia* produces safe and positively received foods

The growth on diverse by-products available across a variety of geographical locations suggested promise for *N. intermedia* as a fungus for waste-to-food conversion for new applications beyond oncom production. However, any such future applications must account for potential issues surrounding the safety (mycotoxin production) and acceptance of *N. intermedia* fermentations among consumers outside the traditional setting of Java, Indonesia.

**Fig. 5:**
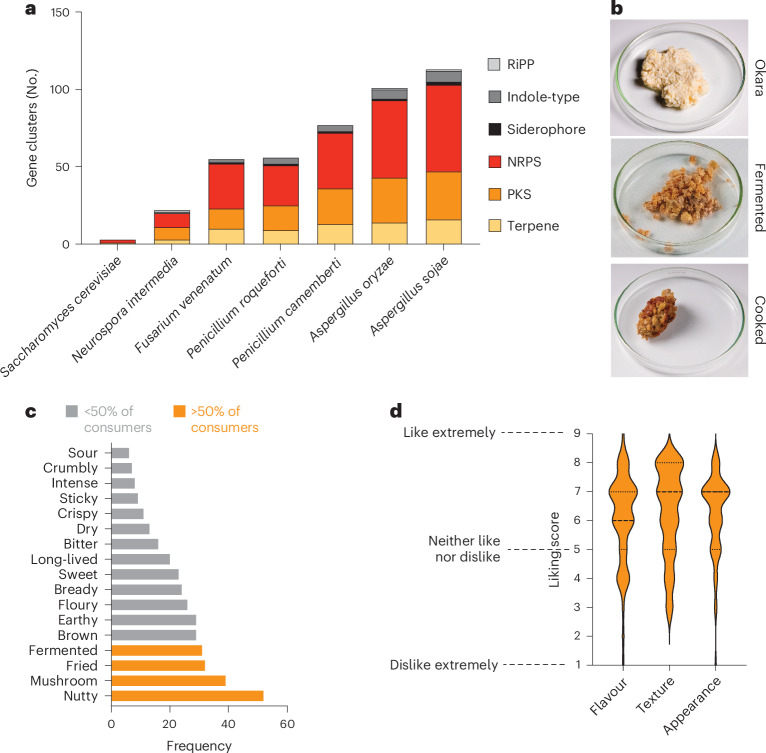
*N. intermedia* has low potential for secondary metabolite production and is positively perceived by consumers outside Indonesia. **a**, Predicted natural products production capacity of *N. intermedia* and other sequenced Ascomycete moulds used for food production. RiPP, ribosomally synthesized and post-translationally modified peptide; NRPS, non-ribosomal peptide synthase; PKS, polyketide synthase. Compared with other ascomycete moulds used for food production, *N. intermedia* encodes for a small number of predicted natural products (*n* = 22) and does not have the genomic capacity to produce any known mycotoxins. Of these, 8 were predicted polyketide synthases and 9 were predicted non-ribosomal-peptide synthases. The full results of the analysis are shown in Supplementary Table 8. **b**, Preparation of fermented, pan-fried okara using *N. intermedia*. The pan-fried okara was used in sensory trials. **c**, Results of a Check-All-That-Apply (CATA) sensory analysis with *n* = 61 adult Danish participants who had never tried oncom. Okara was subjected to fermentation by *N. intermedia* followed by a CATA evaluation of the cooked product. Sensory attributes assigned by >10% of participants are shown in the graph, and those characteristics assigned by >50% of participants are highlighted in orange. **d**, Liking score by the same sensory panel tasting the fermented, cooked okara. Liking scores are shown on the *y* axis. 1 = Dislike extremely, 2 = Dislike very much, 3 = Dislike moderately, 4 = Dislike slightly, 5 = Neither like nor dislike, 6 = Like slightly, 7 = Like moderately, 8 = Like very much, 9 = Like extremely. The mean flavour, texture and appearance was rated >6 by the sensory panel, indicating a liking towards the product. Violin plots show distribution of ratings (dashed line, median; dotted lines, quartiles) for each category for the *n* = 61 participants. Results are shown for *n* = 61 individuals. Source data

A major concern with fungal fermentation is the potential production of secondary metabolites such as mycotoxins that could be harmful to human health^41^. While many of these secondary metabolites may not be produced on the substrates typically used for fermentation, the genomic potential to produce these are concerning when exposing fungi to new substrates or growth conditions^41,42^. To assess this possibility for *N. intermedia*, we next analysed the capacity for secondary metabolite production using the #2613 reference genome (Fig. 5a and Supplementary Table 9). Compared with other sequenced, edible Ascomycete fungi frequently used for food production, *N. intermedia* encoded for a strikingly small number of predicted natural products. For example, *Aspergillus oryzae* (koji mould), a mould that is frequently explored for by-product SSF across scientific and gastronomic contexts^9^, harboured >100 natural product gene clusters, while *N. intermedia* encoded for only 22, with 8 of these being predicted polyketide synthases and 9 being predicted non-ribosomal-peptide synthases. Unlike *Fusarium venenatum*, a fungus used for alternative meat production in Quorn^43^, and *Aspergillus and Penicillium* moulds used for fermented foods^37,42^, *N. intermedia* did not encode for any known mycotoxins. In addition, we could not detect active production of any known mycotoxins or natural products during SSF of okara by *N. intermedia* using untargeted metabolomics (see Methods for details). While the *Neurospora* genus has been proposed as safe on the basis of historical evidence, its traditional use in oncom^44^ and recent animal trials^45^, these results provide initial genomic evidence for the *N. intermedia* safety profile.

Ultimately, however, the broader potential of *N. intermedia* for upcycling will depend not just on safety, but also on sensory appeal outside the traditional context. We next conducted a Check-All-That-Apply (CATA) consumer survey^46^ of fermented, cooked okara among Danish consumers (*n* = 61 participants) who had never tried oncom previously (Fig. 5b and Supplementary Table 10). These participants assigned mainly positive attributes to the cooked product, characterizing it as ‘nutty’, ‘mushroom’, ‘fermented’, ‘brown’ and ‘earthy’ (attributes selected by >50% of respondents) (Fig. 5c). Importantly, the consumers also overall liked the appearance, flavour and texture of the cooked product (Fig. 5d, mean liking score >6 out of 9 across all categories). The favourable rating is in stark contrast to consumer attitudes towards okara, which has a relatively low acceptance threshold even when used as a minor ingredient^47,48^. Both the liking score and the positive attributes suggest that *N. intermedia* by-product fermentation can create foods that are liked outside the context where red oncom is traditionally produced and consumed, highlighting the potential of *N. intermedia* for fungal waste-to-food upcycling in new contexts and applications.

## Discussion

Fungi have promise for upcycling of food waste for increased sustainability and food security, particularly in the conversion of by-products into human food through SSF^1,6,11^. However, engineering fungal by-product transformation for specific outcomes remains challenging, in part due to the limited availability of fast-growing, well-characterized edible fungal strains and our poor understanding of fungal waste-to-food conversion at the genetic and molecular level. Here we used multi-omics analysis of oncom, a traditional Indonesian fermented food produced from readily available by-products, as a model system to identify strains, enzymes and processes that could enable by-product upcycling in SSF. We initially surveyed black and red oncom using metagenomics, but focused further efforts on red oncom due to the fungal dominance of *N. intermedia* and the fact that red oncom is a unique food involving intentional cultivation of *Neurospora*^44^. We found that black oncom harboured more diverse microbial communities than red oncom, and further work is needed to understand the biochemical and genetic basis of black oncom production.

In addition to providing insight into oncom, our study sheds light on human domestication of microorganisms for sustainability challenges. Although many fungi have been domesticated from their wild relatives for food production throughout human history, *N. intermedia* stands out among characterized fungi in that it appears to have been uniquely recruited for waste-to-food conversion^37–40,49,50^. Recent work has revealed that wild *Neurospora* strains probably exist as plant endophytes^51,52^, and we propose that the genomic changes observed among by-product-associated *N. intermedia* reflect a transition to a new lifestyle and ecological niche associated with the human generation of by-products. This unique domestication of *N. intermedia*, alongside its safety, palatability, fast^53^ and robust growth across diverse substrates and culinary versatility^16,54^, could make it particularly suitable for addressing urgent challenges in waste-to-food upcycling. We envision that our findings here could guide the creation of delicious and nutritious foods from diverse by-products in the near future.

This study serves as another reminder that traditional foods may hold clues to addressing global challenges in sustainability^3,55–57^, and highlights the importance of preserving and characterizing these traditions in the face of rapid global change and food system industrialization. While such traditional biological resources could benefit the world, it is critical that new exploration and innovations centred on these practices procure benefits for the original inventors.

## Methods

### Collection and characterization of oncom samples from traditional producers

Oncom samples were collected from 10 red oncom and 6 black oncom producers throughout Western Java, Indonesia. Before sampling, a survey was carried out on the location of the oncom industry and observations related to detailed information on raw materials, the starter culture used and the production process method used. Samples were taken aseptically, placed in sterile plastic, brought to the laboratory using an ice box and immediately used for analysis. Two replicates were collected. One sample was lyophilised for subsequent sequencing analysis, the other one was used fresh to analyse pH and texture. For pH measurements, a total of 50 g of wet sample was aseptically put into 450 ml of buffer, then crushed and homogenized to obtain a sample suspension of 10% dilution. The pH value was measured using a standardized pH meter. A total of 50 ml of the derived suspension was measured for pH and read when the value was stable. Measurements were made twice, then the average was taken. Texture analysis of oncom samples was carried out by visual observation and by using a penetrometer. A previously described framework^13^ was used to score the appearance and texture of the oncom by observing the growth of mould covering the surface of oncom and the texture density of oncom with the following scale: + (poor mould growth, non-compact texture); ++ (decent growth of mould, texture is quite compact and dense); +++ (good mould growth, compact and dense texture); ++++ (very good mould growth, very compact and dense texture). The hardness level was measured using the Precision Scientific Penetrometer (73501) based on the level of penetration of the penetrometer needle into the sample for a certain time^58^. The oncom sample was placed in the space provided, then the needle was inserted vertically above the surface of the sample at five different points. The measurement was carried out for 5 s, so the hardness was expressed as mm per 5 s.

### 16S and ITS amplicon sequencing of oncom samples

#### DNA extraction, PCR, sequencing and sequence processing

Lyophilised oncom samples were ground into a fine powder using a mortar and pestle. Powder was placed into a DNA Isolation Bead Plate. DNA was extracted following Qiagen’s instructions (MagAttract PowerSoil DNA kit, 27100-4-EP) on a KingFisher robot. Bacterial 16S rRNA genes were PCR amplified with dual-barcoded primers targeting the V4 region (515F 5’-GTGCCAGCMGCCGCGGTAA-3’ and 806R 5’-GGACTACHVGGGTWTCTAAT-3’) ITS: (ITSF 5-CCTCCGCTTATTGATATGC-3 ITS2-R CCGTGARTCATCGAATCTTTG), following the protocol in ref. ^59^. Input DNA concentration was normalized before sequencing. Amplicons were sequenced with an Illumina MiSeq system using the 250 bp paired-end kit (v.3). Sequences were analysed using DADA2 (ref. ^60^) and Phyloseq^61^ following the recommended procedures.

### Metagenome sequencing and analysis of oncom samples

#### DNA extraction and library preparation

Lyophilised oncom samples were ground into a fine powder using a mortar and pestle. DNA from the lyophilised material was extracted using the Qiagen MagAttract PowerSoil DNA KF kit (27100-4-EP) using a KingFisher robot. DNA quality was evaluated visually via gel electrophoresis and quantified using a Qubit 3.0 fluorometer (Thermo Fisher). Libraries were prepared using an Illumina Nextera library preparation kit (Illumina).

#### Sequencing, data curation and sequence processing

Paired-end sequencing (150 bp ×2) was done on a NextSeq 500 system. Shotgun metagenomic sequence reads were processed with the Sunbeam pipeline. Initial quality evaluation was done using FastQC v.0.11.5 (http://www.bioinformatics.babraham.ac.uk/projects/fastqc/). Processing occurred in four steps: adapter removal, read trimming, low-complexity-reads removal and host-sequence removals. Adapter removal was done using cutadapt (v.2.6)^62^. Trimming was done with Trimmomatic (v.0.36)^63^ using custom parameters (LEADING:3 TRAILING:3 SLIDINGWINDOW:4:15 MINLEN:36). Low-complexity sequences were detected with Komplexity (v.0.3.6)^64^. For metagenomics analysis, the concatenated paired-end-read fastq files were analysed using kaiju (v.1.9.0)^24,25^. The NCBI BLAST nr+euk database, which contains bacteria, yeasts, viruses and microbial eukaryotes, was used to perform taxonomic analysis. To reduce noise caused by false positives in identification, a 1% abundance filter was applied to define the presence and absence of each taxon^65^. To evaluate how much of the *N. intermedia* genome was recovered in the sequencing of the oncom samples, all fastq files were concatenated and mapped to the *N. intermedia* genome using HiSAT2 (https://daehwankimlab.github.io/hisat2/).

### General growth conditions and husbandry of *N. intermedia* strains

*N. intermedia* was grown on Vogels minimal medium (VMM)^66^ unless otherwise indicated. The standard medium (1 l) was made by combining the 50X salt solution with 15 g sucrose (unless another carbon source was used) and 1 l distilled water. Agar (15 g) were added for solid medium. To generate spore suspensions, which were used for inoculation in liquid and solid-state cultures, *N. intermedia* was grown from glycerol stocks on VMM agar slants for at least 72 h at 30 °C, or until robust pigmented conidial growth was established. Conidial suspensions were generated by adding sterile water to the slants followed by vortexing. Conidial concentrations were established using a haemocytometer. Liquid and solid-state cultures were maintained in the dark unless otherwise indicated.

### Preparation of SSF okara, sensory analysis and GC–MS analysis of volatile aromas

#### Fermented okara preparation

To make okara, 250 g of raw soybeans were soaked with 4 l of water at 4 °C overnight and rinsed with water before processing. It was then mixed with three parts water (1:3 w/w), processed in the Thermomix (Thermomix TM6) (high speed; 20 s) and drained. Immediately, the solid (okara) was separated from the liquid and steamed in the oven for 30 min at 100 °C. Okara was cooled to 30 °C before inoculating with *Neurospora intermedia* #2613. For inoculation, *N. intermedia* was grown on two separate slants on VMM for 1 week at 30 °C. A 12 ml conidial suspension was created by adding distilled water to each slant and the resulting 12 ml solution was added to 300 g of substrate. The oncom was covered loosely with a sterile cloth and incubated for 24 h at 29 °C and 60% relative humidity (RH). After 24 h the spores germinated and the oncom was fermented without cloth for 24 h. After an additional 24 h, the aerial orange mycelium covered all the surface. The samples were kept at −80 °C until analysis.

#### Volatile aroma extraction

To 5 g of each sample (wet weight, okara, oncom, cooked oncom), 11 g of water (containing 0.9% NaCl) was added. The samples were vortexed, placed in stomacher bags (BagPage, Interscience) and homogenized in a laboratory blender (Stomacher 400, Seward) for 2 min at maximum speed. Next, 8 ml were transferred to 20 ml gas-tight vials, 2.88 g of NaCl was added and the samples stored at −80 °C. Ten microlitres of 2-methyl-3-heptanone was used as an internal standard (diluted to 27.2 mg l^−1^ in liquid chromatography–mass spectrometry (LC–MS)-grade methanol). The negative control consisted of distilled water with the same concentration of salt and internal standard^67^. The volatile composition of the samples was determined using headspace solid phase micro-extraction (HS-SPME) in combination with gas chromatography–mass spectrometry (GC–MS), as described below.

#### SPME–GC/MS analysis

We used the method in ref. ^67^ with minor modifications. After the extraction, the samples were incubated at 45 °C for 15 min before inserting into the GC injection port. The GC–MS analysis was performed on a Thermo Scientific TRACE 1310 gas chromatograph equipped with a Thermo Scientific Q Exactive Orbitrap mass spectrometry system with a Thermo fused-silica capillary column of cross-linked TG-5SILMS (30 m × 0.25 mm × 0.25 µm) (Thermo Fisher). The GC conditions were as follows: inlet and transfer line temperatures, 250 °C; oven temperature programme, 40 °C for 2 min, 5 °C min^−1^ to 120 °C for 2 min, 7 °C min^−1^ to 220 °C for 5 min, 50 °C min^−1^ to 325 °C for 3 min; inlet helium carrier gas flow rate, 1 ml min^−1^; split ratio, 20:1. The electron impact (EI)-MS conditions were as follows: ionization energy, 70 eV; ion source temperature, 250 °C; full-scan *m*/*z* range, 30–350 Da; resolution, 60,000; AGC target, 1 × 10^6^; maximum IT, 200 ms. Method of identification: (1) by comparison of the MS spectra with the NIST library and (2) by comparison of RI (Kovat indices). The areas were normalized to the ISTD 2-methyl-3-heptanone. Retention index was based on an Agilent DB-5MS column using C7-C27 as external references. Data were acquired and analysed with Thermo TraceFinder 4.1 software package (Thermo Fisher).

#### Sensory analysis

Sensory analysis was conducted using a hedonic test with a total of 61 consumers from Copenhagen, Denmark. Sensory analysis was designed in compliance with the Declaration of Helsinki and the 2016/679 EU Regulation on the Protection of Natural Persons Regarding the Processing of Personal Data. The study protocol was reviewed and approved by the Ethics Committee at Mondragon Unibertsitatea before analysis. The experimental procedure was explained to the participants who then completed a written consent form indicating voluntary participation in novel food sensory analysis and gave permission for data processing. Okara oncom was prepared to conduct the sensory analysis, following the method above. Approximately 10 g of each sample was cut in squares, fried and served. The test was conducted in the same room, under controlled temperature and relative humidity (20 ± 2 °C; 95 ± 5% RH) and natural illumination. The 9-point hedonic scale (1 = ‘dislike extremely to 9 = ‘like extremely’) was used to evaluate the participants’ level of liking, as well as three different characteristics (flavour, texture and appearance). The CATA attributes used for the sensory attributes were selected on the basis of trials with Tempeh, a similar fungal fermented Indonesian food from the island of Java^68^. All consumers were instructed to rinse their mouths with water between samples.

### Nutritional analysis of raw and fermented okara and other by-products

Nutritional analyses were performed by Cumberland Valley Analytical Services. All samples analysed were lyophilised and ground into a fine powder before analysis. Fibre (crude) was analysed according to the protocol in ‘Fibre (crude) in animal feed and pet food (978.10)’ described in ref. ^69^. Protein (crude) was analysed using the combustion method, following the method of ‘Protein (crude) in animal feed (990.03)’ described in ref. ^69^. The combustion was performed using a Leco FP-528 Nitrogen Combustion Analyzer (Leco). Crude protein was calculated as Nitrogen × 6.25. Fat (crude) was analysed using the protocol described in ‘Crude fat in feeds, cereal grains and forages (2003.05)’ described in ref. ^70^. A Tecator Soxtec System HT 1043 Extraction unit was used for lipid extraction (Tecator). The amino acid composition of all amino acids except tryptophan was analysed using standard acid hydrolysis of the material and following AOAC Official Methods 994.12 and 982.30 (refs. ^70,71^). Tryptophan was analysed by the standard alkaline hydrolysis (Official Method 988) in AOAC Official Methods^70,72^. High-performance liquid chromatography (HPLC) was used for detection of amino acids.

### Extraction and LC–MS analysis of ergothioneine and free amino acids in raw and fermented okara

#### Extraction

All samples were lyophilised before analysis. For extraction, samples were ground into a fine powder using a mortar and pestle, ~30 mg was transferred to bead beating tubes for homogenization (Lysing Matrix Z, MP Biomedicals, 116961050-CF), 1 ml of 20% methanol with 0.1% formic acid was added and samples were subjected to bead beating for 2× 1 min using a Biospec Mini beadbeater. Following bead beating, samples were spun down at 12,000 *g* for 10 min to separate the solids and 500 µl supernatant was transferred to a centrifugal spin filter (Amicon Ultra, Sigma-Aldrich, UFC500324) to remove any particulates and larger molecules (3 kDa cut-off). The flow-through was collected and subjected to analysis by LC–MS.

#### LC–MS analysis

For LC–MS, analytes were chromatographically separated with a Kinetex HILIC column (100 mm length, 4.6 mm internal diameter, 2.6 µm particle size; Phenomenex) at 20 °C using a 1260 Infinity HPLC system (Agilent Technologies). The injection volume for each measurement was 2 µl. The mobile phase was composed of 10 mM ammonium formate (prepared from a pre-made solution; Sigma-Aldrich) and 0.2% formic acid (from an original stock at ≥98% chemical purity; Sigma-Aldrich) in water (as mobile phase A) and 10 mM ammonium formate and 0.2% formic acid in 90% acetonitrile with the remaining solvent being water (as mobile phase B). The solvents used were of LC–MS grade, purchased from Honeywell–Burdick & Jackson. Analytes were separated via the following gradient: linearly decreased from 90% B to 70% B in 4 min, held at 70% B for 1.5 min, linearly decreased from 70% B to 40% B in 0.5 min, held at 40% B for 2.5 min, linearly increased from 40% B to 90% B in 0.5 min, held at 90% B for 2 min. The flow rate was changed as follows: 0.6 ml min^−1^ for 6.5 min, linearly increased from 0.6 ml min^−1^ to 1 ml min^−1^ for 0.5 min, held at 1 ml min^−1^ for 4 min. The total run time was 11 min. The HPLC system was coupled to an Agilent Technologies 6520 quadrupole time-of-flight mass spectrometer (QTOF–MS) with a 1:4 post-column split. Nitrogen gas was used as both the nebulizing and drying gas to facilitate the production of gas-phase ions. Drying and nebulizing gases were set to 12 l min^−1^ and 25 psi, respectively, and a drying gas temperature of 350 °C was used throughout. Fragmentor, skimmer and OCT1 RF voltages were set to 100 V, 50 V and 250 V, respectively. Electrospray ionization (ESI) was conducted in the positive-ion mode with a capillary voltage of 3.5 kV. MS experiments were carried out in the full-scan mode (*m*/*z* 70–1,100) at 0.86 spectra per second for the detection of [M + H]^+^ ions. The instrument was tuned for a range of *m*/*z* 50–1,700. Before LC-ESI-TOF MS analysis, the TOF MS was calibrated with the Agilent ESI-Low TOF tuning mix. Mass accuracy was maintained via reference ion mass correction, which was performed with purine and HP-0921 (Agilent Technologies). Data acquisition was carried out using MassHunter Workstation Software v.B.08.00 (Agilent Technologies). Data processing was carried out using MassHunter Workstation Qualitative Analysis v.B.06.00 and MassHunter Quantitative Analysis v.10.00. External calibration curves were used to quantify the analytes.

### High-performance anion exchange chromatography (HPAEC) for sugar profiling

#### Extraction of free sugars

All samples were lyophilised before analysis. For extraction from solid, samples were ground into a fine powder using a mortar and pestle, ~30 mg was transferred to tubes for homogenization (Lysing Matrix Z, MP Biomedicals, 116961050-CF), 1 ml of 20% methanol with 0.1% formic acid was added and samples were subjected to bead beating for 2× 1 min. Following bead beating, samples were spun down at 12,000 RCF for 10 min to separate the solids and 500 µl supernatant was transferred to a centrifugal spin filter to remove any particulates and larger molecules (3 kDa cut-off) (Amicon Ultra, Sigma-Aldrich, UFC500324). The flow-through was collected and subjected to analysis by HPAEC. For extraction from culture supernatants, 1 ml culture supernatant was collected and subjected to lyophilisation, 1 ml of 20% methanol with 0.1% formic acid was added and samples were extracted by continuous vortexing for 10 min. Samples were then spun down at 12,000 *g* for 10 min and 500 µl supernatant was transferred to a centrifugal spin filter to remove any particulates and larger molecules (3 kDa cut-off) (Amicon Ultra, Sigma-Aldrich, UFC500324). The flow-through was collected and subjected to analysis by HPAEC.

#### Extraction of pectin-bound sugars

The method of extraction was adapted from ref. ^73^. Lyophilised material was ground to a fine powder before boiling in 96% ethanol for 30 min. After a centrifugation step of 5 min at 20,000 *g*, the supernatant was discarded. The resultant pellet was washed with 70% ethanol until the supernatant was clear. Last, the pellet was washed with 100% acetone and dried in a vacuum concentrator. Samples were hydrolysed in 2 N trifluoroacetic acid for 1 h at 120 °C.

#### HPAEC for sugar profiling

The method was adapted from ref. ^73^ with minor modifications. HPAEC with pulsed amperometric detection was performed on an ICS-6000 (Dionex) using a CarboPac PA20 (3 150 mm, Dionex) anion exchange column at a flow rate of 0.4 ml min^−1^. Before sample injection, the column was equilibrated with 5 mM NaOH for 5 min. The elution programme involved two isocratic elution steps with 5 mM NaOH from 0 to 23 min to separate the neutral sugars, followed by a ramp step to 450 mM NaOH from 23.1 to 41 min, which allowed separation of uronic acids and washing of the column. Monosaccharide standards comprised l-Fuc, l-Rha, l-Ara, CGal, d-Glc, d-Xyl, d-GalA and d-GlcA, as well as GlcNac and d-Man. A run of a standard mixture containing the eight monosaccharides was performed with each sample set to enable sample quantitation by linear regression.

### Library preparation and sequencing to generate the high-quality genome of oncom-derived *N. intermedia* FGSC #2613

#### Extraction of high-quality genomic DNA from *N. intermedia*

*N. intermedia* was grown in VMM with sucrose as the sole carbon source at 25 °C and 120 r.p.m. for 72 h. Mycelia were collected by vacuum filtration and immediately ground in a mortar and pestle with liquid nitrogen to generate a fine powder. Approximately 12 g of finely ground mycelium was subjected to further DNA extraction. The mycelium was transferred to a 50 ml falcon tube containing 10 ml lysis buffer (0.15 M NaCl, 0.1 M EDTA, 2% SDS at pH 9.5). Protease K (Thermo Fisher, EO0491) was then added at a final amount of 1 mg. The tube was left overnight at 37 °C with shaking at 90 r.p.m. Samples were then centrifuged at 6,000 *g* for 10 min to pellet the cellular debris. After centrifugation, avoiding the bottom pellet of debris, 15 ml of the supernatant was transferred to a new tube. Distilled water (10 ml) was then added to the supernatant, as well as 25 ml of phenol:chloroform:isoamyl alcohol (25:24:1) reagent (Sigma-Aldrich, P3803). The sample was rotated and shaken to precipitate proteins, spun down at 7,000 RCF for 15 min to separate the layers, and 23 ml of the top aqueous layer was moved to a new 50 ml falcon tube. Nucleic acids present in this fraction were precipitated with 0.6 volumes (14 ml) of ice-cold isopropanol, which had been frozen at −80 °C for 15 min. The precipitated solution was spun down at 7,000 *g* for 10 min to pellet the DNA. Then 6 ml TE buffer (10 mM Tris, 1 mM EDTA pH 8.0) was added and the sample was resuspended. RNA was digested by overnight incubation at 37 °C with RNase A (300 μg, Sigma-Aldrich, 10109142001). Then, an additional protein digestion step was performed by adding 300 μg protease K (Thermo Fisher, EO0491) and incubating at 37 °C for 2 h. A volume of 6 ml of the solution was extracted once with 6 ml of phenol:chloroform:isoamyl alcohol (25:24:1) reagent (Sigma-Aldrich, P3803). DNA in the top aqueous layer was precipitated with 0.6 volumes (3.5 ml) of ice-cold isopropanol, and following centrifugation, the pellet was dried at room temperature, followed by resuspension in TE buffer. To further purify the DNA for PacBio sequencing, we performed an additional cleanup step, which removed contaminating carbohydrates and organic reagents. In this protocol, 150 µl DNA suspension was mixed with 150 µl chloroform and 50 µl TE buffer. Samples were inverted several times to mix, 150 µl of the top layer was removed to a new Eppendorf tube, and 15 µl sodium acetate (pH 5.2) and 450 µl 100% ethanol (ice cold) were added. Following centrifugation to pellet the precipitated DNA, the supernatant was removed and the pellet was washed twice with 800 µl 70% ethanol, followed by air drying for 30 min at room temperature. The final pellet was resuspended in 150 µl TE buffer. DNA quality was assessed by agarose gel electrophoresis and nanodrop. DNA concentration was assessed using the Qubit DNA BR assay quantification kit (Q32850). The DNA was deemed of sufficiently high quality for reference genome sequencing using PacBio and Illumina.

#### Library preparation and genome sequencing of *N. intermedia* #2613

For the PacBio sequencing (multiplexed at >10 kb with Blue Pippin Size Selection, Tubes), an input of 1.5 µg of high-quality genomic DNA (extracted according to the protocol above) was sheared around 10 kb using Megaruptor 3 (Diagenode) or g-TUBE (Covaris). The sheared DNA was treated with exonuclease to remove single-stranded ends, DNA damage repair enzyme mix, end-repair/A-tailing mix and ligated with barcoded overhang adapters using SMRTbell Express Template Prep kit 2.0 (PacBio). Up to 16 libraries were pooled in equimolar concentrations and purified with AMPure PB beads (PacBio). Pooled libraries were size selected using the 0.75% agarose gel cassettes with Marker S1 and High-Pass protocol on the Blue Pippin (Sage Science). PacBio sequencing primer was then annealed to the SMRTbell template library and sequencing polymerase was bound to them using Sequel II Binding kit 2.0. The prepared SMRTbell template libraries were then sequenced on a PacBio Sequel IIe sequencer using sequencing primer, 8M v.1 SMRT cells and v.2.0 sequencing chemistry with 1 × 1,800 sequencing movie run times.

#### Genome assembly and annotation

Filtered subread data were processed with the JGI QC pipeline to remove artefacts. The mitochondria-filtered CCS reads were then assembled with Flye v.2.8.1-b1676 (https://github.com/fenderglass/Flye) [-g 40 M–asm-coverage 50 -t 32–pacbio-hifi] and subsequently polished with two rounds of RACON v.1.4.13 racon [-u -t 36] (https://github.com/lbcb-sci/racon). Small scaffolds (<1 kb) were removed from the assembly. The cleaned nuclear genome assembly was annotated using the JGI Annotation Pipeline^74^. The annotation pipeline uses a combination of ab initio, homology-based and transcriptome-based gene predictors. The best representative gene model at each locus was selected through automated filtering based on homology and transcriptome support to produce the gene model sets available in MycoCosm^74^.

### Growth of *N. intermedia* FGSC #2613 across carbon sources and RNA extraction for sequencing

#### Growth experiment across carbon sources

We adapted the approach used for *Neurospora crassa* in ref. ^35^, with minor modifications. For all RNA-seq experiments, we first grew *N. intermedia* #2613 from a glycerol stock for 7 days on VMM slants harbouring sucrose as the sole carbon source. Conidia were then collected by addition of 4 ml sterile water to the slant, followed by vortexing. Conidia were counted using a haemocytometer and added at a final concentration of 5 × 10^5^ conidia per ml into 3 ml VMM medium harbouring 2% (w/v) sucrose in 24-well Whatman Uniplates. Each well harboured 3 ml medium. Importantly, the bottoms of the wells for each Uniplate were initially scratched with a sharp syringe needle to allow adherence and formation of a mycelial mat. The plates were placed in a shaker (light on) for 2 h at 30 °C to enable conidial germination and initial mat formation. This step was necessary to establish a mat that could be manipulated in subsequent steps. Then, the shaker was turned on at 30 °C (200 r.p.m., light on). After 15 h of growth at 30 °C, mycelial mats were removed from the bottom of the plate where they had attached and were then washed three times in 3 ml of VMM without any added carbon to remove the sucrose medium. The mycelial mats were finally transferred to 1x VMM with the indicated carbon source for induction. All conditions were done in triplicate. In addition to the carbon sources, a set of samples were ‘induced’ in a no-carbon control, which facilitated downstream analysis to identify genes that were uniquely induced on the carbon source of interest. For induction conditions with carbon sources, 2 mM mono and disaccharides were used, while for complex carbon sources, including complex polysaccharides and plant biomass, 1% (w/v) was used. The following complex carbon sources were used in the experiments at 1% (w/v): Okara flour (Renewal Mill), soluble soy polysaccharides (Creative Enzymes Inc, NATE-1284), Avicel PH-101 (Sigma-Aldrich, 11365), rhamnogalacturonan from soy pectic fibre (Megazyme, P-RHAGN) and stachyose hydrate (Sigma-Aldrich, S4001-100MG). The following carbon sources were used in the experiment at 2 mM: d-(+)-raffinose pentahydrate (TCI, R0002), d-(+)-galactose (Sigma-Aldrich, G0750-10G), l-(+)-arabinose (Sigma-Aldrich, A3256-25G), d-(+)-xylose (Sigma-Aldrich, X3877-25G), d-(+)-galacturonic acid monohydrate (Sigma-Aldrich, 48280-25G-F), l-rhamnose monohydrate (Sigma-Aldrich, 83650-50 G), d-(+)-cellobiose (Sigma-Aldrich, C7252-100G) and d-(+)-mannose (MP Biomedicals, 02102250-CF). After 4 h of induction, mycelia were collected over Miracloth filter paper and flash frozen in liquid nitrogen for storage at −80 °C in Lysing Matrix Z tubes (MP Biomedicals, 116961050-CF), which allowed subsequent bead beating upon defrosting for RNA extraction. Three biological replicates were used for each condition.

#### RNA extractions

RNA extractions were performed on −80 °C stored biomass using TRIzol (Invitrogen, 15596026) and chloroform. Half of the mycelial biomass from a 3 ml culture was used for RNA extractions, and 1 ml of TRIzol reagent was added to the bead-beating tube. Tubes containing biomass, TRIzol and beads were bead beaten for 1 min, then allowed to incubate at room temperature for 5 min on a rocker. Chloroform (200 µl) was then added to each tube, which was vortexed and centrifuged for phase separation. Of the aqueous phase from each sample, 400 µl was combined with 400 µl isopropanol and incubated at room temperature for 10 min on a rocker for RNA precipitation. Samples were centrifuged at 4 °C for 10 min. RNA pellets that formed were washed with 75% ethanol and centrifuged at 4 °C for 2 min. Ethanol was removed via pipette, and the RNA pellet was allowed to dry for several minutes with the cap open. The RNA pellet was resuspended in 40 µl water and treated with 2 µl of Turbo DNAse (Thermo Fisher, AM2238) in a 50 μl reaction. After 20 min incubation at 37 °C, RNA was cleaned up using Qiagen RNeasy mini kit (Qiagen, 74104), eluting at the final step in 30 µl RNAse-free water. RNA was tested for quality using agarose gel electrophoresis and nanodrop. RNA was quantified using the Qubit RNA quantification kit (Thermo Fisher, Q10210).

### Library preparation and sequencing of *N. intermedia* FGSC #2613 transcriptomes

Illumina sequencing was used to capture the transcriptomic profile of *N. intermedia* #2613 grown across carbon sources. RNA was extracted according to the protocol above. Plate-based RNA sample preparation was performed on the PerkinElmer Sciclone NGS robotic liquid handling system using Illumina’s TruSeq Stranded mRNA HT sample prep kit utilizing poly-A selection of mRNA following the protocol outlined in the Illumina user guide, and with the following conditions: total RNA starting material was 1 µg per sample and 8 cycles of PCR was used for library amplification. The prepared libraries were then quantified using the KAPA Illumina library quantification kit (Roche, NC2242092) and run on a LightCycler 480 real-time PCR instrument (Roche). The quantified libraries were then multiplexed and the pool of libraries was then prepared for sequencing on the Illumina NovaSeq 6000 sequencing platform using NovaSeq XP v1.5 reagent kits (Illumina, 20028401) and S4 flow cell, following a 2 × 150 indexed run recipe.

### Analysis of transcriptome data from *N. intermedia* FGSC #2613 grown across carbon sources

#### RNA-seq data processing

Raw fastq file reads were filtered and trimmed using the JGI QC pipeline resulting in the filtered fastq file (*.filter-RNA.gz files). Using BBDuk^75^, raw reads were evaluated for artefact sequence by *k*-mer matching (*k*-mer = 25), allowing 1 mismatch, and detected artefact was trimmed from the 3’ end of the reads. RNA spike-in reads, PhiX reads and reads containing any Ns were removed. Quality trimming was performed using the phred trimming method set at Q6. Finally, the reads under the length threshold were removed. Filtered reads from each library were aligned to the reference genome using HISAT2 (v.2.2.0)^76^. Strand-specific coverage bigWig files (fwd and rev) were generated using deepTools (v.3.1)^77^. featureCounts^78^ was used to generate the raw gene counts (counts.txt) file using gff3 annotations. Only primary hits assigned to the reverse strand were included in the raw gene counts (-s 2 -p–primary options). Raw gene counts were used to evaluate the level of correlation between biological replicates using Pearson’s correlation.

#### Differential expression analysis

To determine the genes that change significantly, we only considered as expressed those genes that have at least a sum of 10 counts per million in at least 15 libraries. All comparisons were paired and the profile in each carbon source was compared against the non-carbon-source condition; this was done with the edgeR package^79^. We used the generalized linear model (GLM) likelihood ratio test for this. To determine whether there is consistency between biological replicates, we performed a multidimensional scaling plot of distances between gene expression profiles. The parameters used to call a gene ‘differentially expressed’ between conditions were FDR < 0.05 and log_2_FC > |1|. A common dispersion between replicates of 0.01339982 was calculated and a tagwise dispersion was also calculated. Raw gene counts (counts.txt), not normalized counts, were used for differential gene expression analysis.

#### Annotation of the genes

To annotate genes, orthologous genes between *N. intermedia* and *N. crassa* were obtained. This was done with the OrthoFinder programme^80^. The curated annotation was inherited from *N. crassa* to *N. intermedia*. The general classification as ‘PlantDegradBio’ is based on the *Neurospora crassa* genes predicted to code for plant biomass degrading enzymes and transporters^35^.

#### Co-expression network

With the matrix of accounts, we built a co-expression network using the WGCNA software^81^. The transformation of the count table was performed with the VST (variance stabilizing transformation) function of DESeq2 (ref. ^82^). Subsequently, the Pearson correlation matrix and the weighted adjacency matrix with continuous values of 0 and 1 were obtained. To evaluate scale independence and mean connectivity, we utilized a gradient method by systematically adjusting the power value from 1 to 20. After identifying a degree of independence surpassing 0.90, the construction of a scale-free network was initiated using the blockwiseModules function, employing a power value of 8. To define the modules of the network, the minimum size was established at 30 genes per module and the threshold for merging similar modules was established at 0.15. Furthermore, the weighted adjacency matrix was transformed into a topological overlap measurement matrix (TOM) to estimate connectivity in the network. Finally, we exported the network using the function exportNetworkToCytoscape with a correlation threshold of 0.25. The network was visualized using the Cytoscape v.3.9.1 programme. Network metrics were obtained using Cytoscape v.3.9.1. To determine enriched metabolic pathways in the modules, we used the KEGG tool (https://www.genome.jp/kegg/).

### Time-course profiling of galactose and arabinose in *N. intermedia* #2613 liquid cultures using okara as sole carbon source

Conidia (5 × 10^5^) from *N. intermedia* #2613 were inoculated into 50 ml VMM medium harbouring okara flour as the sole carbon source (1% w/v) and 250 ml flasks were used. Flasks were then left to shake at 160 r.p.m. at 30 °C in the dark. Culture supernatants (1 ml) were removed at regular intervals, spun down to remove the solid material and mycelium, and immediately flash frozen at −80 °C. Clarified culture supernatants were then lyophilised and extracted for sugar analysis using HPAEC (see below).

### Enzyme assays for cellulase detection

For all cellulase assays, we used the fluorescence-based cellulase kit from Abcam (Abcam, ab189817) and followed the standard protocol. All cellulase assays were performed in 96-well plates (Corning, Falcon Tissue Culture Plate, 353072) and fluorescence was measured at 550 nm excitation and 595 nm emission using a BIOTEK Synergy H1 microplate reader (Agilent). Readings were recorded every 3 min for 18 min. For enzyme assays from liquid cultures, 1 ml culture supernatants were removed from cultures by pipetting. These were then spun down to pellet the insoluble material and mycelium. Of the clarified supernatant, 20 or 25 µl was then used as the enzyme source in cellulase assays in the 100 µl reactions. For cellulase assays from solid-state samples, ~300 mg (wet weight) of raw or fermented okara was placed in an Eppendorf tube. Water (1 ml) was added followed by vortexing. To homogenize the sample, sonication was used (10 s on, 25 s off, 2 min total, 25% amplitude). The resulting slurry was spun down at maximum speed to clarify the supernatant, and 25 µl of the resulting supernatant was used as the enzyme source in the 100 µl enzyme reactions. Cellulase activity was calculated using the formula (*B*/(Δ*T* × *V*)) × *D*, where *B* is the amount of resorufin in the sample well calculated from the standard curve (µmol); Δ*T* is the linear phase reaction time *T*2 – *T*1 (min); *V* is the original sample volume added into the reaction well (µl); *D* is the sample dilution factor if the sample was diluted to fit within the standard curve range. Results from calculations were expressed as mU ml^−1^; 1 unit (U) was defined as the amount of enzyme that cleaves the substrate to generate 1.0 μmol of molecule per min.

### Cellulase activity in liquid cultures of *N. intermedia* strains

#### Confirmation of cellulase activity in *N. intermedia* #2613 liquid cultures

To confirm the presence of cellulases suggested by the RNA-seq data, we replicated the RNA-seq culture setup in which the cellulase gene expression was first detected. We first grew *N. intermedia* #2613 from a glycerol stock for 7 days on VMM slants harbouring sucrose as the sole carbon source. Conidia were then collected by addition of 4 ml sterile water to the slant, followed by vortexing. Conidia were counted using a haemocytometer and added at a final concentration of 5 × 10^5^ conidia per ml into 3 ml VMM medium harbouring 2% (w/v) sucrose in 24-well Whatman Uniplates. The bottoms of the wells for each Uniplate were initially scratched with a sharp syringe needle to allow adherence and formation of a mycelial mat. The plates were placed in a shaker (light on) for 2 h to enable conidial germination and initial mat formation. This step was necessary to establish a mat that could be manipulated in subsequent steps. Then, the shaker was turned on (200 r.p.m., light on). After 15 h of growth, mycelial mats were removed from the bottom of the plate where they had attached and were then washed three times in 3 ml of VMM without any added carbon to remove the sucrose medium. The mycelial mats were finally transferred to 1x VMM with either sucrose (1.5% w/v), avicel (1% w/v), okara flour (1% w/v) or rhamnogalacturonan (1% w/v). All conditions were done in triplicate. Cultures were left for 72 h at 30 °C and supernatants were then analysed for cellulase activity using the protocol above.

#### Screen for secreted cellulase activity in liquid cultures among *N. intermedia* strains grown on okara

We first grew *N. intermedia* strains from glycerol stocks for 7 days on VMM slants harbouring sucrose as the sole carbon source. Conidia were then collected by addition of 4 ml sterile water to the slant, followed by vortexing. The following strains were used from the by-product-associated clade: #2613, #2685, #2559, #5644, #1791, #5642, #5342. The following strains were used from the burn-associated clade: #1316, #8767, #8761, #1938, #1785, #7426, #7402, #3194. Conidia were counted using a haemocytometer and added at a final concentration of 5 × 10^5^ conidia per ml into 250 ml flasks with 50 ml VMM medium harbouring 1% (w/v) okara flour as the sole carbon source. All strains were grown in triplicate and the experiment was repeated three times to verify the consistency of the results. Cultures were left for 72 h at 30 °C and supernatants were analysed for cellulase activity using the protocol above. Biomass was collected and dried at the end of the experiment. The biomass was dried for 7 days at 50 °C before the weight was recorded.

### Genome sequencing of additional *N. intermedia* strains beyond the oncom-derived reference strain *N. intermedia* #2613

#### Extraction and sequencing

*N. intermedia* strains subjected to sequencing (FGSC #2559, #2685, #5644, #2557, #1791, #5642, #5342) were grown in VMM-sucrose medium (50 ml medium in 250 ml flasks) for 72 h before collection by vacuum filtration and flash freezing in liquid nitrogen. Genomic DNA extraction, sample quality assessment, DNA library preparation, sequencing and bioinformatics analysis were conducted at Azenta Life Sciences. Genomic DNA was extracted using DNeasy Plant mini kit following manufacturer instructions (Qiagen, #69204). Genomic DNA was quantified using the Qubit 2.0 fluorometer (Thermo Fisher). NEBNext Ultra II DNA Library Prep kit for Illumina (New England Biolabs, #E7645L) clustering and sequencing reagents were used throughout the process following manufacturer recommendations. Briefly, the genomic DNA was fragmented by acoustic shearing with a Covaris S220 instrument. Fragmented DNA was cleaned up and end repaired. Adapters were ligated after adenylation of the 3’ ends, followed by enrichment by limited cycle PCR. DNA libraries were validated using a High Sensitivity D1000 ScreenTape on the Agilent TapeStation (Agilent Technologies) and quantified using the Qubit 2.0 fluorometer. The DNA libraries were also quantified by real-time PCR (Applied Biosystems). The sequencing library was clustered onto the lanes of an Illumina HiSeq 4000 (or equivalent) flow cell. After clustering, the flow cell was loaded onto the Illumina HiSeq instrument according to manufacturer instructions. The samples were sequenced using a 2 × 150 bp paired-end configuration. Image analysis and base calling were conducted using the HiSeq Control Software. Raw sequence data (.bcl files) generated from Illumina HiSeq were converted into fastq files and demultiplexed using Illumina bcl2fastq 2.17 software. One mismatch was allowed for index sequence identification.

#### Assembly and annotation

The reads were filtered with TrimmomaticPE (v.0.39)^63^ with the following parameters: LEADING:30 TRAILING:30 MINLEN:120. The filtered reads were used for de novo assembly using the SPAdes genome assembler (v.3.13.1-1)^83^ with the following parameters: –careful–cov-cut-off 100. The resulting assemblies were then processed with AUGUSTUS (v.3.4.0)^84^ to obtain coding sequences and protein predictions. Augustus was executed using a gene model for *Neurospora crassa* to identify start and stop codons, introns and exons.

### Genomic and phylogenomic analysis of *N. intermedia* strains

#### Core/pan genome analysis for *N. intermedia*

In addition to the seven *N. intermedia* draft genomes obtained in our study using short reads and the genome of *N. intermedia* strain #2613 obtained using long and short reads, we retrieved the genomes of 29 *Neurospora* spp. strains from a previous study^28^ and 5 strains which were obtained from the public GenBank database. These genomes were annotated following the protocol described above and their protein sequences were used for core genome calculation using BPGA^85^ with a sequence identity cut-off of 0.5; this analysis led to a set of 6,416 conserved orthologous proteins. The functional analysis of the resulting sets of core and unique genes was done with blast2go^86^ implemented in omicsBox v.3.029 (https://www.biobam.com/omicsbox).

The taxonomic affiliation of the *N. intermedia* strains used in this study was defined by a multilocus phylogenetic tree constructed with the set of conserved orthologous proteins found in their core genome. For each set of orthologues, the amino acid sequences were aligned^28^ and trimmed^87^; after this process, 6,353 alignments were kept. They were then concatenated to form a matrix with 3,203,239 sites in 6,353 partitions. An evolutionary model was calculated for each partition. Then a phylogenetic tree was calculated with IQtree2 (v.2.0.7)^88^ using maximum likelihood with 10,000 bootstrap replicates. The entire process was executed automatically using a script available at https://github.com/WeMakeMolecules/Core-to-Tree.

#### SNP analysis

For the identification and analysis of SNPs, we assembled a set of DNA sequencing reads for a total of 28 *N. intermedia* strains obtained with the Illumina Hi-seq platform in the 2 × 150 paired-end-read format. This dataset included the reads obtained for the 6 *N. intermedia* strains sequenced in this project and 22 more from a previous study^28^. Strain FGSC #2613 obtained for this project was used as reference.

The reads were downloaded from the genbank FTP using fastq-dump v.2.113 (https://github.com/ncbi/sra-tools). Each of the 28 paired reads were aligned with Bowtie 2 (v.2.4.4)^89^, the Sequence Alignment and Map (SAM) formatted files where then converted to binary alignment and map (BAM) format and sorted using samtools (v.1.13)^90^. The sorted and bam-formatted alignment and mapping files were then used for variant calling, using the mpileup, call, view and filter utilities of the bcftools package (v.1.13)^90^. For this, we set the ploidy to 1 and the quality cut-off for the SNPS of QUAL = >50. A simple script to perform these operations is available at https://github.com/WeMakeMolecules/SNP_CALL_and_MAPPING/raw/main/map_and_vcf.sh.

We used a simple script to process the variant call files (vcf formatted) to obtain a table with the number of SNPs per gene in each of the analysed strains when compared with strain 2613. The script is available at https://github.com/WeMakeMolecules/SNP_CALL_and_MAPPING/raw/main/raw_snps_counter.pl.

The raw SNP counts were divided by the length of their corresponding genes in kilobases to obtain the SNP per Kilobase value. To select for genes that have accumulated significantly more mutations (outliers) and that may be implied in the emergence of new food-associated traits we (1) identified genes with SNPs per Kb value of equal to or more than the average SNPs per Kb value for that gene plus two standard deviations (that is, 2 × s.d. + average), then (2) we selected genes with values that passed this filter in at least 10 of the strains located in the burnt-vegetation clade. The functional analysis of the resulting set of outlier genes was done with blast2go^86^ implemented in omicsBox v.3.029 (https://www.biobam.com/omicsbox).

### Screen for growth of *N. intermedia* across okara and other by-products in solid-state cultures

By-products were received from restaurants and large-scale industrial food producers who wished to remain anonymous. By-products that were received in dry form were soaked overnight and then drained to remove excess moisture, accomplishing a % moisture of ~50% w/w. By-products were then autoclaved for 15 min at 121 °C for sterilization. For inoculation, we first grew *N. intermedia* #2613 from a glycerol stock for 7 days on VMM slants harbouring sucrose as the sole carbon source. Conidia were then collected by addition of 4 ml sterile water to the slant, followed by vortexing. Of this conidial suspension, 2 ml was added to ~50 g of the solid material, which was then mixed by hand to distribute the conidia. The inoculated by-products were placed in square Petri dishes with the lid on (Greiner Bio, 688102) and grown at 30 °C for 72 h. Autoclaved, uninoculated by-products were saved for comparison. Growth was assessed by visual inspection and a growth score was assigned on the basis of the development of mycelia and conidia within and around the substrate and compared with okara, which served as a positive control to benchmark the high growth. Scoring was as follows: 0 = no growth, 1 = minimal growth, 2 = moderate growth, 3 = high growth. Samples were lyophilised for storage and a subset of samples was subjected to protein analysis.

### Natural products prediction across *N. intermedia* and other edible ascomycetes

For the prediction of natural product production capacity, the genome assembly and gene calling file for strain #2613 was then used for functional annotation and mining for natural products biosynthetic gene clusters using Antismash (v.7)^91^. The same gene calling and annotation process described above in ‘Genome sequencing of additional *N. intermedia* strains beyond the oncom-derived reference strain N. intermedia #2613’ was done for the genomes of *Saccharomyces cerevisiae* YJM1342 (GenBank accession GCA_000977265.3), *Fusarium venenatum* A3/5 (GenBank accession GCA_900007375.1), *Penicillium roqueforti* JCM 22842 (GenBank accession GCA_001599855.1), *Penicillium camemberti* FM013 (GenBank accession GCA_014839975.1), *Aspergillus oryzae* RIB40 (GenBank accession GCF_000184455.2) and *Aspergillus sojae* SMF134 (GenBank accession GCA_008274985.1).

### Metabolomic analysis of secondary metabolite production of *N. intermedia*

#### Extraction

Lyophilised powdered samples of *N. intermedia* (2 mg) grown in okara obtained by SSF were transferred to vials, followed by the addition of 2 ml methanol. The vials were placed in an ultrasound bath for 60 min. At the end of this period, centrifugation was performed and the supernatant was then transferred to a 1.5 ml glass vial in preparation for subsequent LC–MS/MS analysis.

#### LC–MS/MS data acquisition and untargeted metabolomics analysis

Untargeted metabolomics analysis of extracts was performed using LC–MS/MS on a Vanquish Duo UHPLC binary system (Thermo Fisher) coupled to an IDX-Orbitrap mass spectrometer (Thermo Fisher). The chromatographic separation was performed using a Waters ACQUITY BEH C18 column (10 cm × 2.1 mm, 1.7 μm) equipped with an ACQUITY BEH C18 guard column kept at 40 °C, with a flow rate of 0.35 ml min^−1^. The mobile phases consisted of MilliQ water + 0.1% formic acid (A) and acetonitrile + 0.1% formic acid (B). The initial composition was 2% B held for 0.8 min, followed by a linear gradient to 5% in 3.3 min, and then a second gradient until reaching 100% B in 10 min. This condition was then held for 1 min. The system re-equilibration time was 2.7 min before loading the next sample into the instrument. The MS measurement was done in positive and negative-heated electrospray ionization (HESI) mode with voltages of 3,500 V and 2,500 V, respectively. Full MS/MS spectra DDA (data-dependent acquisition-driven MS/MS) were acquired within the mass range of 70–1,000 Da. The DDA acquisition settings were as follows: automatic gain control (AGC) target value set at 4 × 10^5^ for full MS and 5 × 10^4^ for MS/MS spectral acquisition, and the mass resolution was set to 120,000 for full-scan MS and 30,000 for MS/MS events. Precursor ions were fragmented by stepped high-energy collision dissociation (HCD) using collision energies of 20, 40 and 60. To search for known natural products and mycotoxins in *N. intermedia* grown in okara, the tandem mass spectrometry data were analysed using the Global Natural Product Social Molecular Networking (GNPS)^92^ platform (available online at https://gnps.ucsd.edu/ProteoSAFe/static/gnps-splash.jsp) and SIRIUS (4)^93^. For MS/MS dereplication via molecular networking analysis and SIRIUS 4, MS/MS data were converted to mzXML format using MS-Convert software, which is part of ProteoWizard.

### Statistics and reproducibility

No statistical method was used to predetermine sample size. *n* = 3 was chosen as the minimal number of replicates for experimental characterization. We determined this to be sufficient based on internal controls (with *N. intermedia* grown on okara) to capture biological variability between replicates. No data were excluded from the analyses. The experiments were not randomized. The investigators were not blinded to allocation during experiments and outcome assessment.

### Reporting summary

Further information on research design is available in the Nature Portfolio Reporting Summary linked to this article.

## Supplementary information

Supplementary Information Supplementary Note 1, Figs. 1–6 and captions for Tables 1–12.

Reporting Summary

Supplementary Tables 1–12 Single file with all supplementary tables (1–12) as individual tabs, with captions provided in Supplementary Information file.

## Source data

Source Data Fig. 1 Source data.

Source Data Fig. 2 Source data.

Source Data Fig. 3 Source data.

Source Data Fig. 4 Source data.

Source Data Fig. 5 Source data.

Source Data Extended Data Fig. 2 Source data.

Source Data Extended Data Fig. 3 Source data.

Source Data Extended Data Fig. 4 Source data.

Source Data Extended Data Fig.5 Source data.

Source Data Extended Data Fig. 6 Source data.

Source Data Extended Data Fig. 8 Source data.

Source Data Extended Data Fig. 9 Source data.

## Figures and Tables

**Extended Data Table 1:**
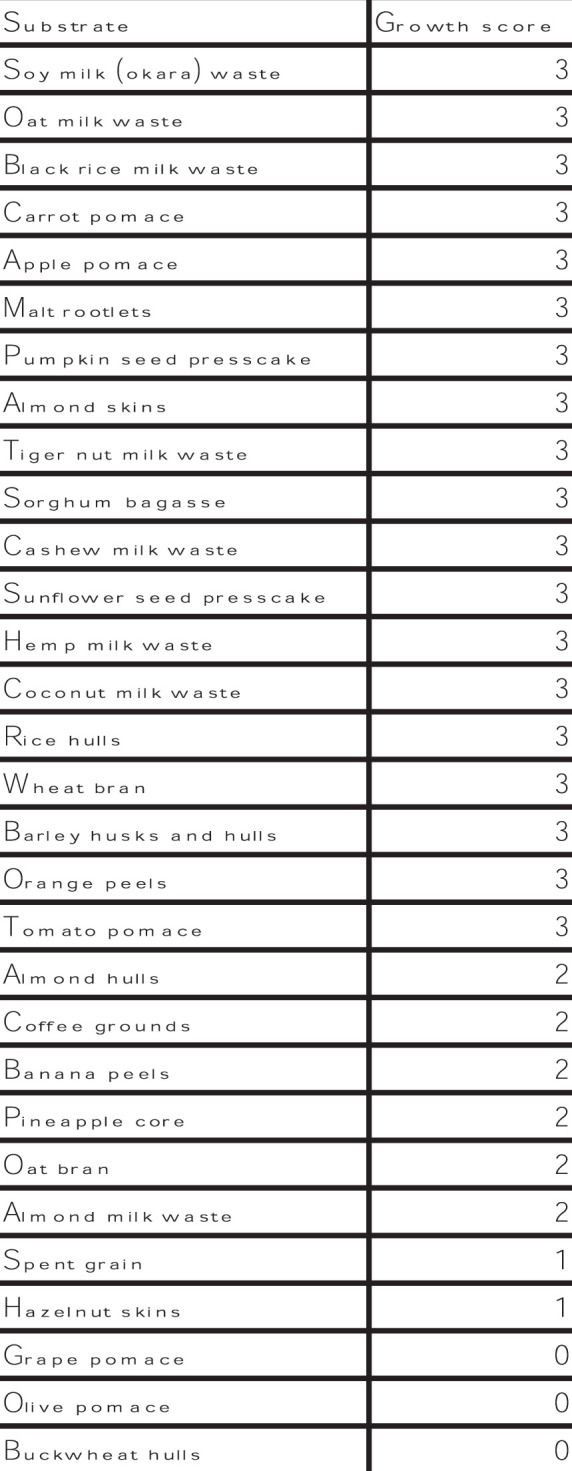
Growth of *N. intermedia* on diverse byproducts

We quantified the growth by visual inspection and assigned a growth score based on the amount of mycelia that had formed within and around the substrate (0 = no growth, 1 = minimal growth, 2 = moderate growth, 3 = high growth). Okara was used as a control to benchmark the high growth. Photos are found in Fig. 4 in main text and in the source data.

## Data Availability

The genome assembly and annotation of *Neurospora intermedia* FGSC #2613 is available through the MycoCosm portal at https://mycocosm.jgi.doe.gov/Neuin1 and GenBank through Bioproject PRJNA982925. The transcriptomics data from *N. intermedia* FGSC #2613 grown across carbon source has been deposited to the Sequence Read Archive (SRA); the specific access information is specified in Supplementary Table 11. The genomes and reads for *N. intermedia* strains #1791, #2557, #2559, #2685, #5342, #5642 and #5644 are deposited at Genbank under Bioproject PRJNA996151; the sequencing information can be found in Supplementary Table 12. The 16S and ITS amplicon sequencing, as well as the metagenome sequencing data have also been deposited at Genbank under Bioproject PRJNA996151. All other data are available in the supplementary material and the source data files. Source data are provided with this paper.

## Code availability

The scripts used for phylogenetic and SNP analysis of *N. intermedia* have been deposited to GitHub and are available through Zenodo^94,95^.
