# Supplementary material for: Neurospora intermedia from a traditional fermented food enables waste-to-food conversion
Source: Nat Microbiol. 2024 Aug 29;9(10):2666–83. doi: 10.1038/s41564-024-01799-3 (PMC11445060; doi:10.1038/s41564-024-01799-3)
Supplement: Supplementary file 1 — Supplementary Note 1, Figs. 1–6 and captions for Tables 1–12. [file 41564_2024_1799_MOESM1_ESM.pdf]

nature microbiology

Supplementary information https://doi.org/10.1038/s41564-024-01799-3

Neurospora intermedia from a traditional fermented food enables waste-to-food conversion

In the format provided by the authors and unedited

Supplementary information

Neurospora intermedia from a traditional fermented food enables waste-to-food conversion

Vayu Maini Rekdal et al.

Supplementary note 1

Black oncom samples displayed significant microbial variability between producers compared to red oncom. For instance, *Rhizopus*, assumed to be the main driver of black oncom fermentation, was detected only in five of six black samples, with the sample collected from Ciawi harboring no detectable level of *Rhizopus* and instead being dominated by bacteria (Fig. 1F of main text). Within the samples containing *Rhizopus*, the abundance of this fungus varied widely, ranging from complete dominance to comprising less than 10% of total assigned reads. It was also not always the most abundant fungus, and overall, black oncom harbored a diversity of fungi beyond *Rhizopus*, including *Geotrichum*, which is typically associated with dairy fermented foods[1], *Mucor,* which is found in diverse Asian fermented foods[2], *Meyerozyma,* a yeast that grows in a variety of habitats[3], and *Apophysomyces*, a fungus associated with subtropical climates[4] (Fig. 1E and 1F of main text). Bacterial communities also varied between samples. Notable bacteria included *Burkholderia*[5] and *Mycetohabitans*[6]*,* which are known endophytes of *Rhizpous*, as well as lactic acid bacteria such as *Weisella* and *Lactococcus,* which were present in several samples (Fig. 1E and 1F of main text). Such bacteria may be involved in the lactic acid fermentation that takes place during the soaking of the peanut presscake prior to inoculation (Fig. 1A of main text). Finally, it is worth noting that the microbial community composition of red oncom differs from that of other microbially characterized soy-based foods from Asia, including miso, dajiang-meju, qu, soy sauce, sufu, and cheonggukjang. These communities do not contain *Neurospora* and instead harbor microbial communities that vary across product type and include fungi such as *Aspergillus, Rhizopus, Penicillium*, as well as diverse bacteria[7-11].

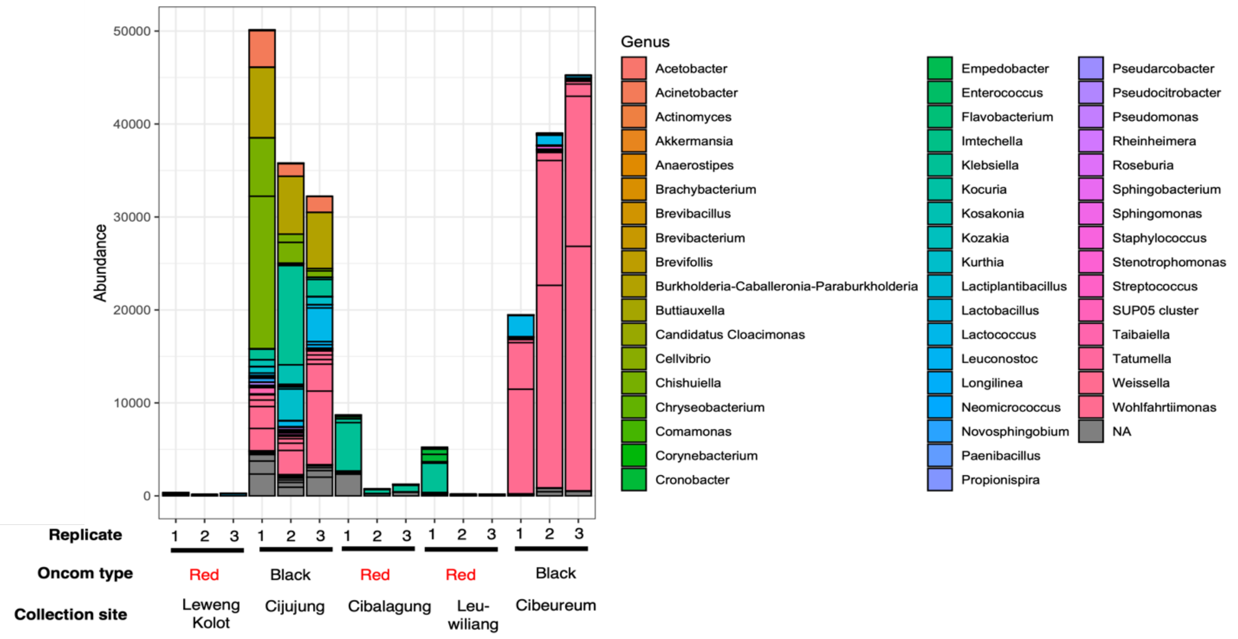

Supplementary Figure 1. Bacterial abundance based on 16s amplicon sequencing of a subset of red and black oncom samples collected from traditional producers in Java, Indonesia. The graph displays the bacterial abundance at the genus level. DNA concentrations were normalized prior to sequencing. Analysis was conducted using phyloseq. Bars indicate the number of reads assigned to a specific genus. Three replicates from each of the five oncom products collected from different producers were sequenced. While the black oncom sample from Cijujung had a diverse microbial community, including *Weisella*, *Brevibacterium*, *Klebsiella*, and *Acetobacter,* the black oncom sample from Cibeureum was mostly dominated by the lactic acid bacterium *Weissella,* which is consistent with the substrates being soaked and subjected to a brief lactic acid fermentation prior to inoculation. In contrast, red oncom samples did not harbor a diverse microbial community. Some red oncom samples harbored enterobacteria such as *Klebsiella.*

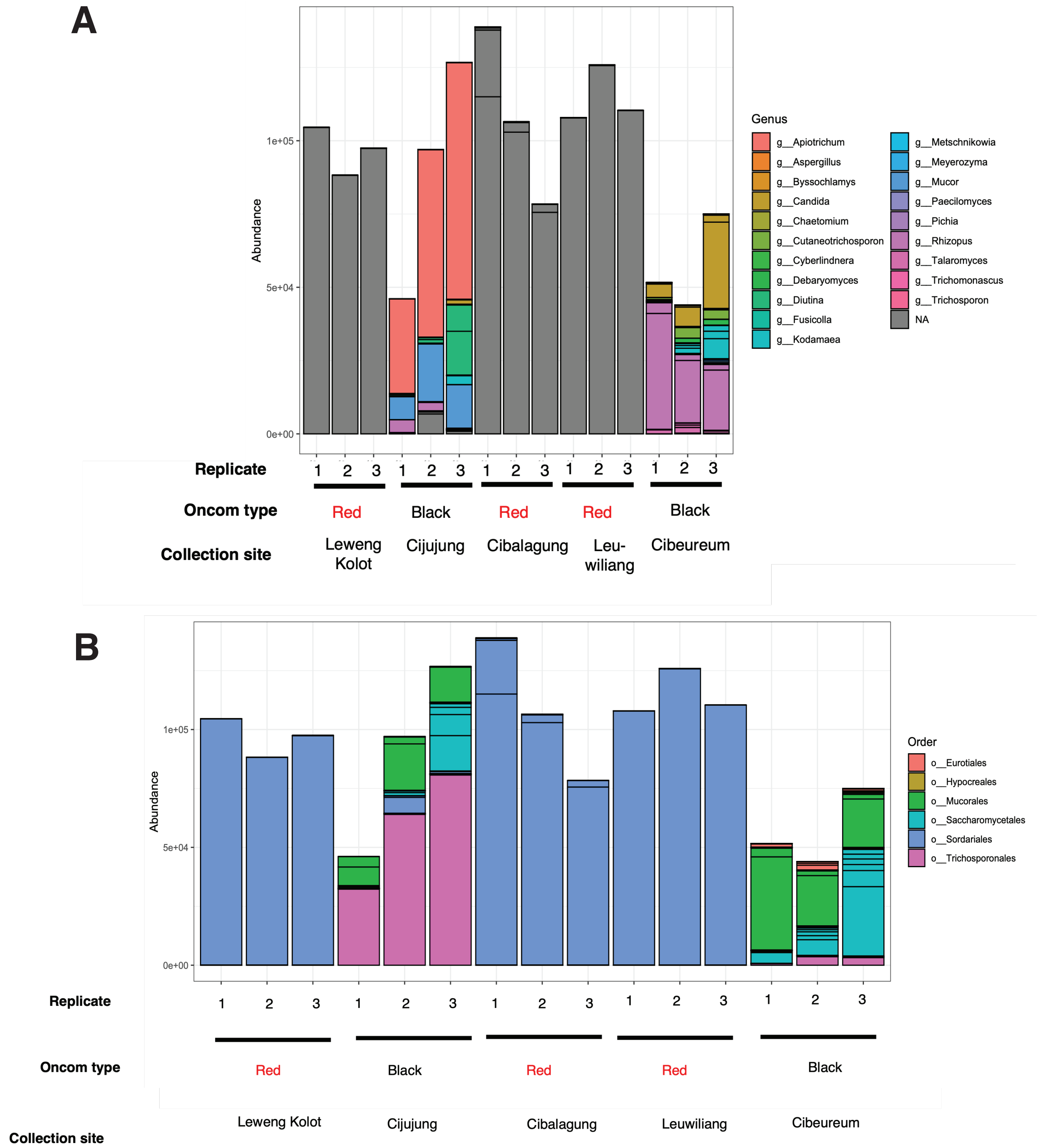

Supplementary Figure 2. Fungal abundance based on ITS amplicon sequencing of a subset of red and black oncom samples collected from traditional producers in Java, Indonesia. The graph displays the fungal abundance at the genus (A) and order (B) levels. DNA concentrations were normalized prior to sequencing. Analysis was conducted using phyloseq. Bars indicate the number of reads assigned to a specific group. Three replicates from each of the five oncom "cakes"

collected from different producers were sequenced. A) At the genus level, black and red oncom samples harbored different fungal communities, and while red oncom appeared to be dominated by a single genus, the two black oncom samles harbored different fungal communities. However, the specific genus identity in the red oncom samples could not be assigned using the analysis pipeline and database. Black oncom from Cijujung was dominated by *Apotrichium* but also harbored *Mucor* and *Debaromyces* members. The other black oncom sample was dominated by *Rhizopus*. B) Order-level assignment revealed that the unassigned major fungus at the genus level belonged to the *Sordariales* order. Comparison of these results with the metagenome sequencing (see Figure 1 in main text) suggests that the major fungus is *Neurospora*. There was also a small number of a fungus belonging to the *Sordiaria* genus detected based on metagenomic sequencing (Figure 1 in main text), which may explain the presence of two distinct *Sordariales* fungi (based on ITS amplicon sequencing data) at the order level in the Cibalagung sample.

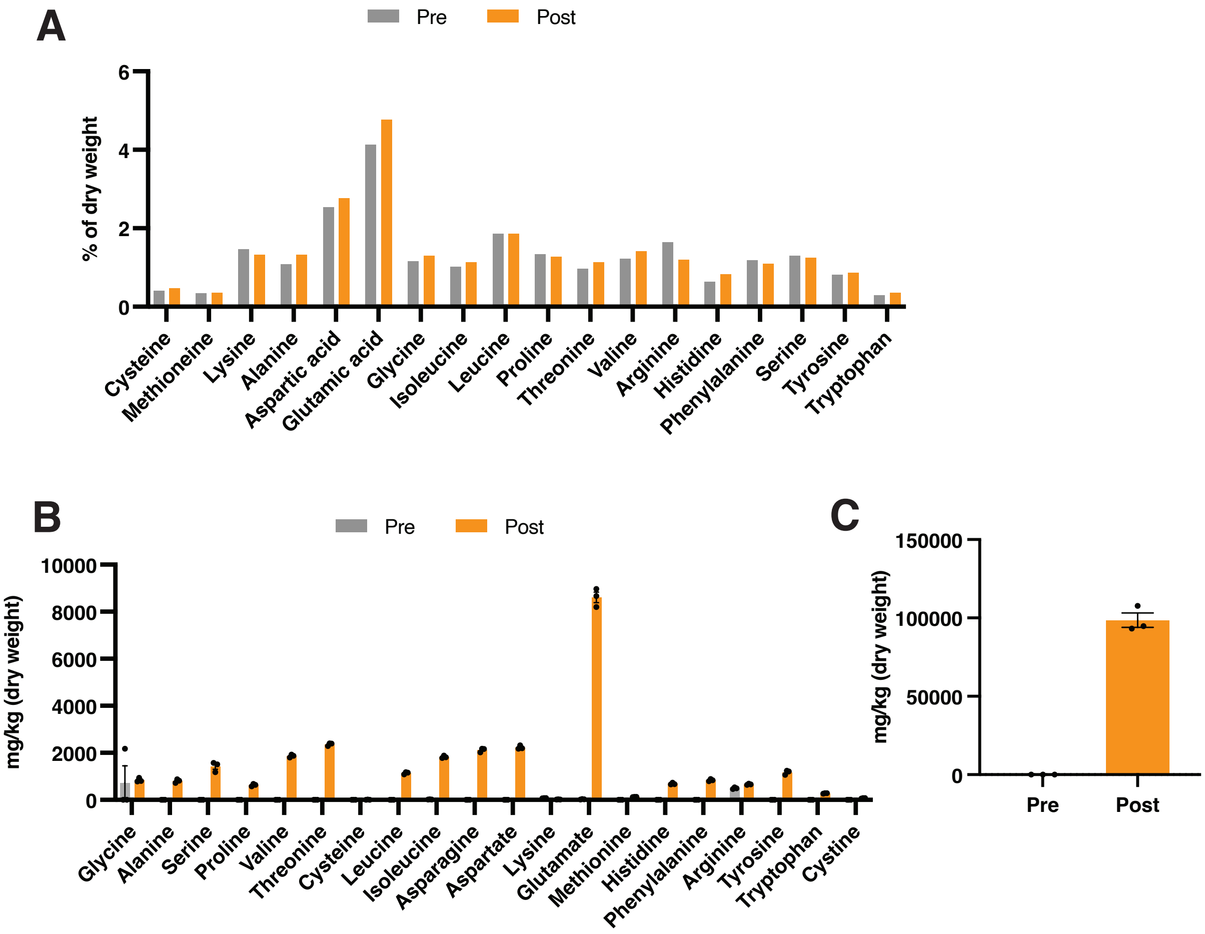

Supplementary Figure 3. Amino acid changes in okara fermented by *N. intermedia.* A) Amino acid composition of raw and fermented okara. Okara samples were fermented with *N. intermedia* for 48 hours in solid-state. Lyophilized, hydrolyzed samples were subjected to amino acid profiling. The graph demonstrates that there were no major changes in the global amino acid composition before and after fermentation. However, like raw okara, fermented okara contained all the essential amino acids (histidine, isoleucine, leucine, lysine, methionine, phenylalanine, threonine, tryptophan, and valine). One lyophilized sample of each of okara substrate and fermented okara was analyzed. B) and C) Free amino acids in raw and fermented okara**.** B) Okara samples were fermented with *N. intermedia* in solid-state. Lyophilized samples were extracted and

subjected to amino acid analysis by LC-MS. Results are mean and SEM of three biological replicates and are normalized to the dry weight. A) shows all analyzed amino acids except for glutamine, which was produced at such high levels that it obscured changes in the other amino acids. Aside from glutamine, glutamate (8610 mg/kg) was by far the most produced amino acids. Only arginine was present at similar amounts pre and post fermentation. C) Levels of glutamine pre and post fermentation. This was produced at the highest levels of all amino acids (98571 mg/kg).

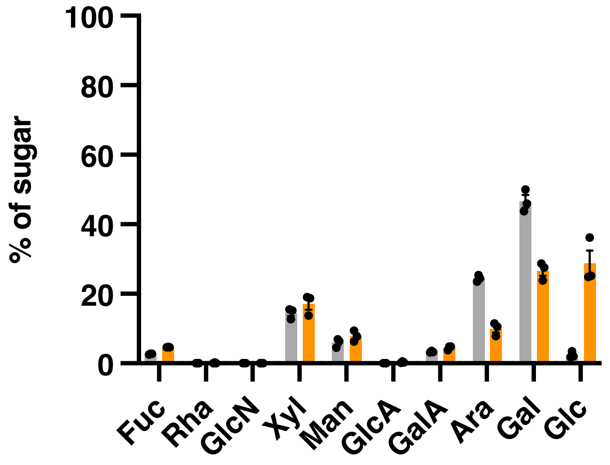

Supplementary Figure 4. Sugar composition in the hydrolyzed pectin fraction of raw and fermented okara. Okara samples were fermented with *N. intermedia* for 48 hours in solid-state. Lyophilized samples were extracted and subjected to sugar analysis by HPAEC after processing and hydrolysis to obtain the pectin-containing fraction. Results are mean and SEM of three biological replicates and are expressed as the mol % of all sugars analyzed. Fuc = fucose, Rha = rhamnose, GlcN = N-acetylglucosamine, Xyl = xylose, Man = mannose, GlcA = glucuronic acid, GalA = galacturonic acid, Ara = arabinose, Gal = galactose, Glc = glucose. There was a decrease in both arabinose and galactose as a result of the fermentation. Other sugars did not decrease significantly. The increase in glucose is likely attributed to glucans, which make up the majority of the fungal cell wall and can be found in the pectin fraction after fermentation. Arabinose and galactose are abundant in rhamnogalacturonan, the main pectic fiber in soy, and the position of these sugars within the side chain of the polymer makes them accessible for hydrolytic release and microbial degradation, which is consistent with the data. There was not a significant decrease of galacturonic acid, which is found in more inaccessible portions of the pectin polymer. Grey = okara substrate; Orange=post fermentation.

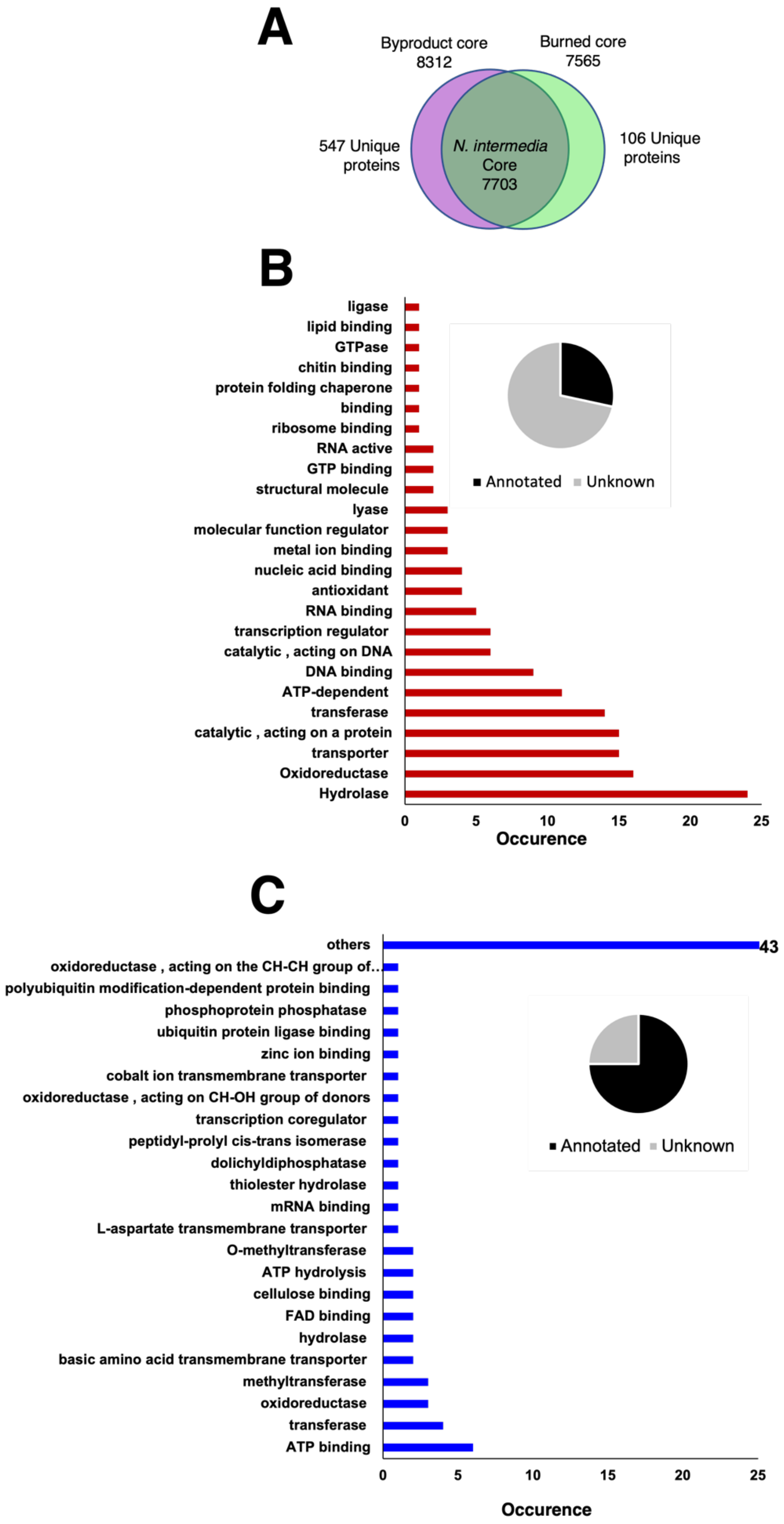
A
Byproduct core
8312
Burned core
7565
547 Unique proteins
N. intermedia Core 7703
106 Unique proteins
B
ligase
lipid binding
GTPase
chitin binding
protein folding chaperone
binding
ribosome binding
RNA active
GTP binding
structural molecule
lyase
molecular function regulator
metal ion binding
nucleic acid binding
antioxidant
RNA binding
transcription regulator
catalytic , acting on DNA
DNA binding
ATP-dependent
transferase
catalytic , acting on a protein
transporter
Oxidoreductase
Hydrolase
0
5
10
15
20
25
Occurence
Annotated
Unknown
C
others
43
oxidoreductase , acting on the CH-CH group of..
polyubiquitin modification-dependent protein binding
phosphoprotein phosphatase
ubiquitin protein ligase binding
zinc ion binding
cobalt ion transmembrane transporter
oxidoreductase , acting on CH-OH group of donors
transcription coregulator
peptidyl-prolyl cis-trans isomerase
dolichyldiphosphatase
thiolester hydrolase
mRNA binding
L-aspartate transmembrane transporter
O-methyltransferase
ATP hydrolysis
cellulose binding
FAD binding
hydrolase
basic amino acid transmembrane transporter
methyltransferase
oxidoreductase
transferase
ATP binding
0
5
10
15
20
25
Occurence
Annotated
Unknown

Supplementary Figure 5. Pan-genome analysis of two genetically distinct subpopulations of *Neurospora intermedia* associated with byproducts and burned vegetation. Phylogenetic analysis (Figure 3 in main text) revealed the existence of two closely related, genetically distinct subpopulations of *N. intermedia* associated with burned vegetation and byproducts, respectively. A) The core *N. intermedia* genome consists of 7703 shared protein-coding genes. The pan-genome of the byproduct-associated strains is several-fold larger compared to the burn-associated strains, suggesting potential genetic expansion among the byproduct clade. Functional enrichment analysis of the byproduct associated (B) and burn-associated (C) pan-genomes. BLASTGO was used to identify the predicted metabolic functions among the genes that are unique to each clade. Hydrolytic activity was the most enriched clade among the byproduct-associated strains (B). However, less than half of all genes could be functionally annotated, suggesting many genes with unknown functions. C) In contrast to the byproduct-associated pan-genome, the burn-associated pangenome was enriched in diverse functions and was more disperse in terms of the number of genes for each functionally assigned category. The burn-associated pan-genome also had a higher amount of annotated functions.

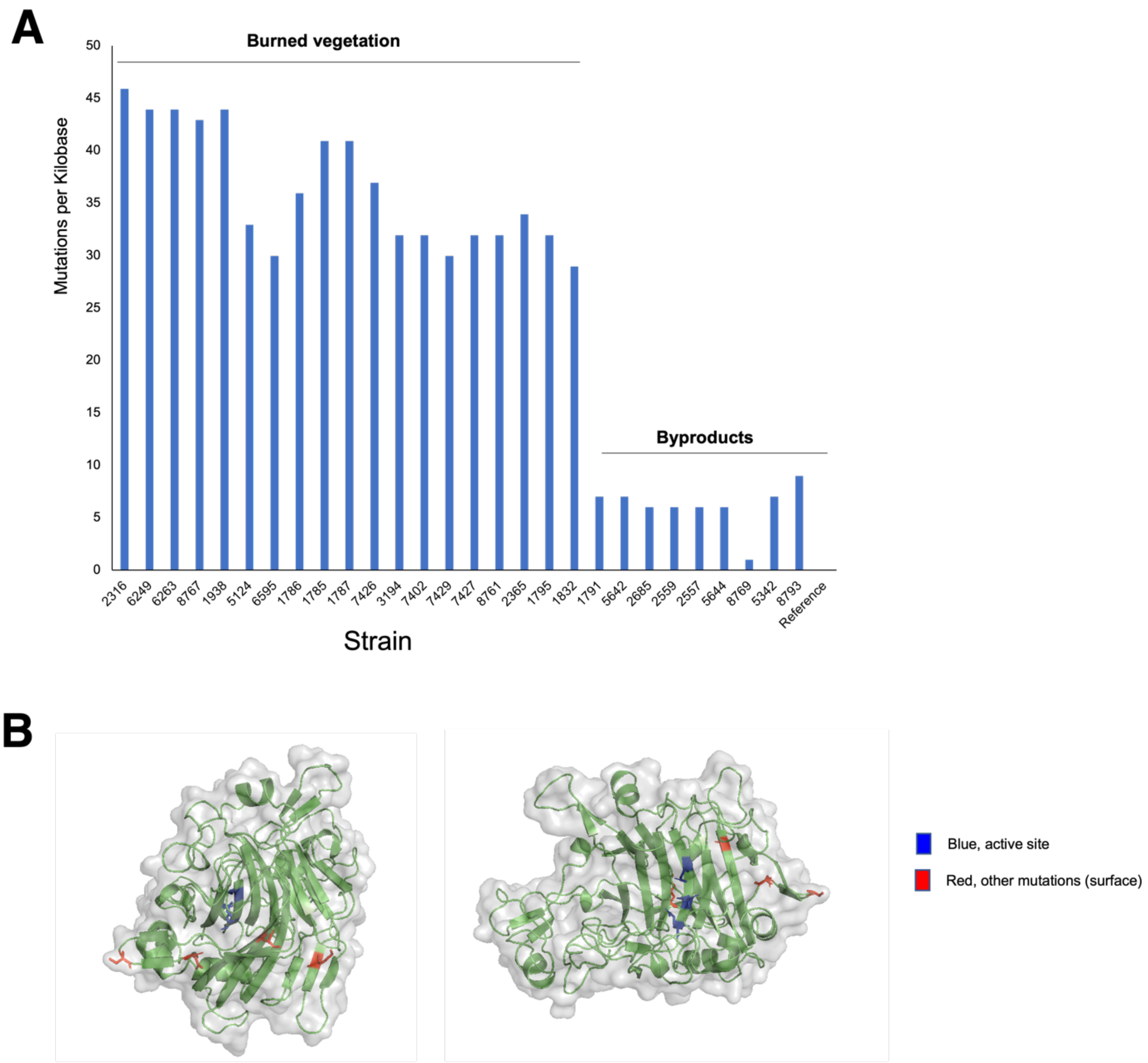

Supplementary Figure 6. Mutational profile and structural modeling in the predicted cellulase (#480919) belonging to the glycosyl hydrolase 7 (GH 7) family. A) SNPs in the enzyme among all *N. intermedia* strains analyzed in this study. Data are normalized per kb. There was a clear difference in the mutational profile between the two groups. B) Structural modeling of predicted mutations in the GH 7 enzyme, based on *T. reesei Cel7B.* Alphafold was used to generate the model. The predicted mutations were located away from the active site and are located on the surface.

Supplementary table 1. Physicochemical and geographical data for oncom samples collected from traditional producers in Java, Indonesia. Samples were collected from regional producers throughout Western Java. The site where the sample was collected is indicated in the table, in both name and coordinates of the geographical location. pH was measured using a pH meter, and hardness was measured using a penetrometer. Compactness was determined visually (see methods for details). Results are mean and SD of two replicates for pH, five replicates for hardness. + (poor mold growth, non-compact texture); ++ (decent growth of mold, texture is quite compact and dense); +++ (good mold growth, compact and dense texture); ++++ (very good mold growth, very compact and dense texture). See methods for description of compactness measurements.

Supplementary table 2. Genome statistics for oncom-derived *Neurospora intermedia* FGSC #2613. *Neurospora intermedia* FGSC #2613, which was isolated from oncom samples, was subjected to whole genome sequencing using PacBio and was annotated using transcriptomics, generating a high-quality draft genome that is publicly available through the Joint Genome Institute (JGI). The completeness of the genome outperforms the previous best *N. intermedia* genome, which was sequenced in an unrelated study[12]. *N. crassa,* the model ascomycete and the first *Neurospora* to ever be sequenced, is included for comparison.

Supplementary table 3. GC-MS analysis of volatile aroma compounds in raw okara as well as raw and cooked okara fermented with *N. intermedia*. Values are expressed in mean and standard deviation (SD). Values are expressed as 2-methyl-3-heptanone equivalent (µg/L). Compounds were identified by comparison of the MS spectra with the NIST library as well as by comparison of RI (Kovat indices). Retention index is based on Thermo TG-5SILMS column using C7-C27 as external references. A total of 30 volatile compounds were found in all the samples, including one acid compound, three aldehydes, one ester, two ethers, four ketones, 17 hydrocarbons and one phenol. The flavor of the original okara is described as having grassy, green and beany aromas, all undesirable notes normally found in bean products[13]. These attributes can be traced to hexanal, a product of enzymatic oxidation due to the presence of lipoxygenase during processing of the raw materials[14]. After the fermentation, the abundance was reduced 40-times from 86.86 µg/L in okara to 2.47 µg/L in oncom. Additionally, the acid compound 2-methyl butyric acid was found in okara but disappeared during the fermentation. It is produced during anaerobic fermentation of short-chain fatty acids[15] and is responsible for cheesy and rancid aromas[16]. In addition, the cooked fermented okara developed hydrocarbon compounds related to the cooking method, such as 2-ethyl-1,4-dimethyl-benzene (the most abundant), dodecane, and others[17]. Moreover, ethylcyclohexane developed during cooking; it is responsible for pineapple and pear aromas, and is directly related to roasted attributes developing via the Maillard reaction[18].

Supplementary table 4. Transcriptomics results for *N. intermedia* grown on okara. Genes differentially expressed above a cutoff of log2foldchange>1 are shown and a statistical cutoff of FDR<0.05. Results are from three biological replicates compared to a no carbon control, in liquid cultures. CAZyme, BLASTgo, and degradPlantBio annotations are displayed for functional predictions. degradPlantBio is based on homologous genes involved in plant biomass degradation in the related fungus and model ascomycete *Neurospora crassa*[19]. The JGI protein ID comes from the genome assembly and annotation of Neurospora intermedia FGSC #2613, which is available through the MycoCosm portal at https://mycocosm.jgi.doe.gov/Neuin1.

Supplementary table 5. Genes induced by *N. intermedia* in response to okara above cutoff threshold. Genes induced above a cutoff of log2foldchange>4 are shown and a statistical cutoff of FDR<0.05. Results are from three biological replicates with okara as the sole carbon source in (1% w/v), compared to a no carbon control, in liquid cultures. CAZyme, BLASTgo, and degradPlantBio annotations are displayed for functional predictions. degradPlantBio is based on homologous genes involved in plant biomass degradation in the related fungus and model ascomycete *Neurospora crassa.* Okara induced a number of predicted CAZymes. The JGI protein ID comes from the genome assembly and annotation of Neurospora intermedia FGSC #2613, which is available through the MycoCosm portal at https://mycocosm.jgi.doe.gov/Neuin1.

Supplementary Table 6. *Neurospora* strains analyzed in this study. Strains of *N. intermedia* experimentally evaluated in this study (see Fig. 3 in main text for exact strains that were evaluated in this study) were obtained from the Fungal Genetics Stock Center (FGSC)[20] and their corresponding FGSC numbers are shown. Clade refers to the two clades identified in Figure 3 in the main text. The ecological association data for clades A and B was obtained from FGSC. Svedberg et al (2018) refers to the following paper[12].

Supplementary Table 7. Analysis of SNPs between byproduct and burn-associated strains of *N. intermedia.* The table contains information about SNP calling and analysis among the *N. intermedia* strains shown in clades A and B in Figure 3 of the main text. SNPs were mapped relative to the reference strain #2613. To identify the most highly mutated genes (n=209), we identified genes that harbored mutations at 2x standard deviations above the average mutation rate across the genome, in at least 10 of the burn-associated strains.

Supplementary Table 8. Protein content in a subset of byproducts before and after SSF by *N. intermedia*. The growth of *N. intermedia* #2613 across byproducts is shown in Figure 4 in the main text. We analyzed the protein content of a subset of byproducts on which *N. intermedia* displayed high growth after 72 hours in SSF. Results shown are % protein by dry weight pre and post fermentation for lyophilized samples (one per each substrate).

Supplementary Table 9. Predicted natural products gene clusters in edible ascomycete fungi. Predicted natural products production capacity of *N. intermedia* and other sequenced Ascomycete molds used for food production. The results are graphically represented in Figure 4 in the main text. RiPP = ribosomally synthesized and post-translationally modified peptide; NRPS = nonribosomal peptide synthase; PKS = polyketide synthase. Predictions were generated using ANTISMASH.

Supplementary Table 10. Check all that apply (CATA) sensory analysis of cooked okara fermented with *N. intermedia.* N=61 Danish consumers who had never before consumed oncom were asked to label the sensory attributes of okara subjected to solid-state fermentation by *N. intermedia* and then cooked in oil in a pan. The sensory attributes are shown alongside their frequency. Attributes with the same frequency are shown in the same cell. Overall, the fermented product was assigned positive attributes such as nutty, mushroom, fermented, fried, earthy, brown, floury, sweet and bready. Together with the liking score in Figure 1 in the main text, those positive attributes suggest that okara fermented with *N. intermedia* can be accepted outside the context in which oncom is traditionally produced and consumed. The sensory attributes were derived from similar trials with Tempeh[21].

Supplementary Table 11. Data availability for *N. intermedia* FGSC #2613 transcriptomics data in the public Sequence Read Archive. Data can be accessed through https://www.ncbi.nlm.nih.gov/bioproject.

Supplementary Table 12. Genome information for additional *N. intermedia* strains sequenced as part of this study and deposited to the Sequence Read Archive. FGSC[20] numbers are shown next to the species name.

References

1 Kamilari, E., Stanton, C., Reen, F. J. & Ross, R. P. Uncovering the Biotechnological Importance of Geotrichum candidum. *Foods* **12**, 1124 (2023).

2 Hesseltine, C. W. in *Frontiers in Industrial Mycology* (Ed. Gary F. Leatham) 24-39 (Springer US, 1992).

3 Ghasemi, R. *et al.* Meyerozyma guilliermondii species complex: review of current epidemiology, antifungal resistance, and mechanisms. *Braz J Microbiol* **53**, 1761-1779 (2022). https://doi.org:10.1007/s42770-022-00813-2

4 Chakrabarti, A. *et al.* Apophysomyces elegans: epidemiology, amplified fragment length polymorphism typing, and in vitro antifungal susceptibility pattern. *J Clin Microbiol* **48**, 4580-4585 (2010). https://doi.org:10.1128/jcm.01420-10

5 Lackner, G., Moebius, N., Partida-Martinez, L. P., Boland, S. & Hertweck, C. Evolution of an endofungal Lifestyle: Deductions from the Burkholderia rhizoxinica Genome. *BMC Genomics* **12**, 210 (2011). https://doi.org:10.1186/1471-2164-12-210

6 Yang, S., Anikst, V. & Adamson, P. C. Endofungal Mycetohabitans rhizoxinica Bacteremia Associated with Rhizopus microsporus Respiratory Tract Infection. *Emerg Infect Dis* **28**, 2091-2095 (2022). https://doi.org:10.3201/eid2810.220507

7 Xie, M. *et al.* An integrated metagenomic/metaproteomic investigation of microbiota in dajiang-meju, a traditional fermented soybean product in Northeast China. *Food Research International* **115**, 414-424 (2019). https://doi.org:https://doi.org/10.1016/j.foodres.2018.10.076

8 Tamang, J. P. *et al.* Shotgun metagenomics of Cheonggukjang, a fermented soybean food of Korea: Community structure, predictive functionalities and amino acids profile. *Food*

Research International **151**, 110904 (2022). https://doi.org:https://doi.org/10.1016/j.foodres.2021.110904

9 Caffrey, E. B., Olm, M. R., Kothe, C. I., Evans, J. & Sonnenburg, J. L. MiFoDB, a workflow for microbial food metagenomic characterization, enables high-resolution analysis of fermented food microbial dynamics. *bioRxiv*, 2024.2003.2029.587370 (2024). https://doi.org:10.1101/2024.03.29.587370

10 Chen, C. *et al.* Metagenomic and metaproteomic analyses of microbial amino acid metabolism during Cantonese soy sauce fermentation. *Front Nutr* **10**, 1271648 (2023). https://doi.org:10.3389/fnut.2023.1271648

11 He, W. & Chung, H. Y. Exploring core functional microbiota related with flavor compounds involved in the fermentation of a natural fermented plain sufu (Chinese fermented soybean curd). *Food Microbiol* **90**, 103408 (2020). https://doi.org:10.1016/j.fm.2019.103408

12 Svedberg, J. *et al.* Convergent evolution of complex genomic rearrangements in two fungal meiotic drive elements. *Nat Commun* **9**, 4242 (2018). https://doi.org:10.1038/s41467-018-06562-x

13 Zhaojun Wang, T. G., Zhiyong He, Maomao Zeng, Fang Qin, Jie Chen. Reduction of off-flavor volatile compounds in okara by fermentation with four edible fungi. *LWT* **55** (2022). https://doi.org:https://doi.org/10.1016/j.lwt.2021.112941

14 Lan, Y., Xu, M., Ohm, J. B., Chen, B. & Rao, J. Solid dispersion-based spray-drying improves solubility and mitigates beany flavour of pea protein isolate. *Food Chem* **278**, 665-673 (2019). https://doi.org:10.1016/j.foodchem.2018.11.074

15 Cavinato, C., Da Ros, C., Pavan, P. & Bolzonella, D. Influence of temperature and hydraulic retention on the production of volatile fatty acids during anaerobic fermentation of cow manure and maize silage. *Bioresour Technol* **223**, 59-64 (2017). https://doi.org:10.1016/j.biortech.2016.10.041

16 Lin, J. *et al.* Qualitative and quantitative analysis of volatile constituents from latrines. *Environ Sci Technol* **47**, 7876-7882 (2013). https://doi.org:10.1021/es401677q

17 Siddique, R., Zahoor, A. F., Ahmad, H., Zahid, F. M. & Karrar, E. Impact of different cooking methods on polycyclic aromatic hydrocarbons in rabbit meat. *Food Sci Nutr* **9**, 3219-3227 (2021). https://doi.org:10.1002/fsn3.2284

18 Guo, S., Na Jom, K. & Ge, Y. Influence of Roasting Condition on Flavor Profile of Sunflower Seeds: A flavoromics approach. *Sci Rep* **9**, 11295 (2019). https://doi.org:10.1038/s41598-019-47811-3

19 Wu, V. W. *et al.* The regulatory and transcriptional landscape associated with carbon utilization in a filamentous fungus. *Proc Natl Acad Sci U S A* **117**, 6003-6013 (2020). https://doi.org:10.1073/pnas.1915611117

20 McCluskey, K., Wiest, A. & Plamann, M. The Fungal Genetics Stock Center: a repository for 50 years of fungal genetics research. *J Biosci* **35**, 119-126 (2010). https://doi.org:10.1007/s12038-010-0014-6

21 Rahmawati, D., Astawan, M., Putri, S. P. & Fukusaki, E. Gas chromatography-mass spectrometry-based metabolite profiling and sensory profile of Indonesian fermented food (tempe) from various legumes. *J Biosci Bioeng* **132**, 487-495 (2021). https://doi.org:10.1016/j.jbiosc.2021.07.001
