# Supplementary material for: Neurospora intermedia from a traditional fermented food enables waste-to-food conversion
Source: Nat Microbiol. 2024 Aug 29;9(10):2666–83. doi: 10.1038/s41564-024-01799-3 (PMC11445060; doi:10.1038/s41564-024-01799-3)
Supplement: Supplementary file 2 — Reporting Summary [file 41564_2024_1799_MOESM2_ESM.pdf]

nature portfolio

Corresponding author(s): Jay Keasling

Last updated by author(s): 06-29-24

Reporting Summary

Nature Portfolio wishes to improve the reproducibility of the work that we publish. This form provides structure for consistency and transparency in reporting. For further information on Nature Portfolio policies, see our Editorial Policies and the Editorial Policy Checklist.

Statistics

For all statistical analyses, confirm that the following items are present in the figure legend, table legend, main text, or Methods section.

n/a	Confirmed	
☐	☒	The exact sample size (*n*) for each experimental group/condition, given as a discrete number and unit of measurement
☐	☒	A statement on whether measurements were taken from distinct samples or whether the same sample was measured repeatedly
☐	☒	The statistical test(s) used AND whether they are one- or two-sided<br>*Only common tests should be described solely by name; describe more complex techniques in the Methods section.*
☒	☐	A description of all covariates tested
☒	☐	A description of any assumptions or corrections, such as tests of normality and adjustment for multiple comparisons
☐	☒	A full description of the statistical parameters including central tendency (e.g. means) or other basic estimates (e.g. regression coefficient) AND variation (e.g. standard deviation) or associated estimates of uncertainty (e.g. confidence intervals)
☐	☒	For null hypothesis testing, the test statistic (e.g. *F*, *t*, *r*) with confidence intervals, effect sizes, degrees of freedom and *P* value noted<br>*Give P values as exact values whenever suitable.*
☒	☐	For Bayesian analysis, information on the choice of priors and Markov chain Monte Carlo settings
☒	☐	For hierarchical and complex designs, identification of the appropriate level for tests and full reporting of outcomes
☐	☒	Estimates of effect sizes (e.g. Cohen's *d*, Pearson's *r*), indicating how they were calculated

Our web collection on statistics for biologists contains articles on many of the points above.

Software and code

Policy information about availability of computer code

Data collection	No custom code was used in the collection of data
Data analysis	Scripts used for phylogenetic and SNP analysis are available through Github (https://github.com/WeMakeMolecules)<br>Other software used for analysis:<br>DADA2 v 1.26 (https://benjjneb.github.io/dada2/index.html)<br>Phyloseq v 1.48 (https://www.bioconductor.org/packages/release/bioc/html/phyloseq.html)<br>FastQC v 0.11.5 (http://www.bioinformatics.babraham.ac.uk/projects/fastqc/)<br>cutadapt v2.6<br>Trimmomatic v 0.36<br>Komplexity v0.3.6<br>kaiju v1.9.0<br>NCBI BLAST<br>R v 4.3.3<br>HiSAT2 (https://daehwankimlab.github.io/hisat2/)<br>Thermo TraceFinder 4.1<br>MassHunter Workstation Qualitative Analysis v B.06.00<br>MassHunter Quantitative Analysis v 10.00<br>Flye v 2.8.1-b1676 (https://github.com/fenderglass/Flye)<br>RACON v 1.4.13 racon [-u -t 36] (https://github.com/lbcb-sci/racon)<br>BBduk (https://jgi.doe.gov/data-and-tools/software-tools/bbtools/bb-tools-user-guide/bbduk-guide/)<br>deepTools v3.1

blast2go (in omicsBox v3.029)
IQtree2 v 2.0.7
fastq-dump v 2.113 (https://github.com/ncbi/sra-tools)
samtools v 1.13
bcftools package v1.13
GNPS (https://gnps.ucsd.edu/ProteoSAFe/static/gnps-splash.jsp)
TrimmomaticPE v 0.39
SPAdes genome assembler v 3.13.1-1
AUGUSTUS v3.4.0

featureCounts
edgeR package v 3.19
Orthofinder (https://github.com/davidemms/OrthoFinder)
WGCNA Gene Correlation Network Analysis
DESeq2
Cytoscape v 3.9.1

For manuscripts utilizing custom algorithms or software that are central to the research but not yet described in published literature, software must be made available to editors and reviewers. We strongly encourage code deposition in a community repository (e.g. GitHub). See the Nature Portfolio guidelines for submitting code & software for further information.

Data

Policy information about availability of data

All manuscripts must include a data availability statement. This statement should provide the following information, where applicable:

- Accession codes, unique identifiers, or web links for publicly available datasets
- A description of any restrictions on data availability
- For clinical datasets or third party data, please ensure that the statement adheres to our policy

The genome assembly and annotation of Neurospora intermedia FGSC #2613 is available through the MycoCosm portal at https://mycocosm.jgi.doe.gov/Neuin1 and GenBank through Bioproject PRJNA982925. The transcriptomics data from Neurospora intermedia FGSC #2613 grown across carbon source has been deposited to the Sequence Read Archive (SRA); the specific access information is specified in Supplementary table 11 in the supplemental materials. The genomes and reads for N. intermedia strains #1791, #2557, #2559, #2685, #5342, #5642, and #5644 are deposited at the Genbank under Bioproject PRJNA996151; the sequencing information can be found in Supplementary table 12 in the supplemental materials. The 16s and ITS amplicon sequencing, as well as the metagenome sequencing, has also been deposited at the Genbank under Bioproject PRJNA996151. All other data are available in the supplementary material and the source data files.

Research involving human participants, their data, or biological material

Policy information about studies with human participants or human data. See also policy information about sex, gender (identity/presentation), and sexual orientation and race, ethnicity and racism.

Reporting on sex and gender	The study did not collect any information about sex or gender.
Reporting on race, ethnicity, or other socially relevant groupings	The study did not collect any information about race, ethnicity, etc.
Population characteristics	The study did not collect any information about population characteristics.
Recruitment	The recruitment was done by email. This could have introduced self-selection bias among participants, for example attracting persons particularly interested in foods. The large sample size (n=61) was chosen to as one way to address this possibility and better represent consumer attitudes among the tested population.
Ethics oversight	The study protocol was reviewed by the Ethics Committee at Mondragon Unibertsitatea

Note that full information on the approval of the study protocol must also be provided in the manuscript.

Field-specific reporting

Please select the one below that is the best fit for your research. If you are not sure, read the appropriate sections before making your selection.

☒ Life sciences ☐ Behavioural & social sciences ☐ Ecological, evolutionary & environmental sciences

For a reference copy of the document with all sections, see nature.com/documents/nr-reporting-summary-flat.pdf

Life sciences study design

All studies must disclose on these points even when the disclosure is negative.

Sample size	No statistical method was used to predetermine sample size. n=3 was chosen as the minimal number of replicates for experimental characterization. We determined this to be sufficient based on internal controls (with N. intermedia grown on okara) to capture biological variability between replicates.
Data exclusions	No data were excluded from the study
Replication	All attempts at replication were successful. Experiments were repeated at least twice.
Randomization	Randomization was not relevant due to the nature of the study of assessing microbial/biochemical under controlled conditions
Blinding	Blinding was not relevant to the study as experimental conditions were evident from the experimental procedures (for example, fermented, unfermented).

Reporting for specific materials, systems and methods

We require information from authors about some types of materials, experimental systems and methods used in many studies. Here, indicate whether each material, system or method listed is relevant to your study. If you are not sure if a list item applies to your research, read the appropriate section before selecting a response.

Materials & experimental systems

n/a	Involved in the study	
☒	☐	Antibodies
☒	☐	Eukaryotic cell lines
☒	☐	Palaeontology and archaeology
☒	☐	Animals and other organisms
☒	☐	Clinical data
☒	☐	Dual use research of concern
☒	☐	Plants

Methods

n/a	Involved in the study	
☒	☐	ChIP-seq
☒	☐	Flow cytometry
☒	☐	MRI-based neuroimaging

Plants

Seed stocks	N/A
Novel plant genotypes	N/A
Authentication	N/A
