# Supplementary material for: Neurospora intermedia from a traditional fermented food enables waste-to-food conversion
Source: Nat Microbiol. 2024 Aug 29;9(10):2666–83. doi: 10.1038/s41564-024-01799-3 (PMC11445060; doi:10.1038/s41564-024-01799-3)
Supplement: Supplementary file 6 — Source data. [file 41564_2024_1799_MOESM6_ESM.zip › Fig3-sourcedata/Fig3-sourcedata-Fig3Btree/readme.txt]

Readme.txtSUPER_MATRIX.zip=Matrix file=the sequences used for the treeSUPERMATRIX.partitions=Partition file=partition file for tree sequences These two can be used to reconstruct treeSUPERMATRIX.partitions.contree=Consensus tree = is the actual phylogeny.Core_pan_Genome_plot=Pangenome data = used for the plot in the figure
