# Supplementary figures and images for: Neurospora intermedia from a traditional fermented food enables waste-to-food conversion
Source: Nat Microbiol. 2024 Aug 29;9(10):2666–83. doi: 10.1038/s41564-024-01799-3 (PMC11445060; doi:10.1038/s41564-024-01799-3)

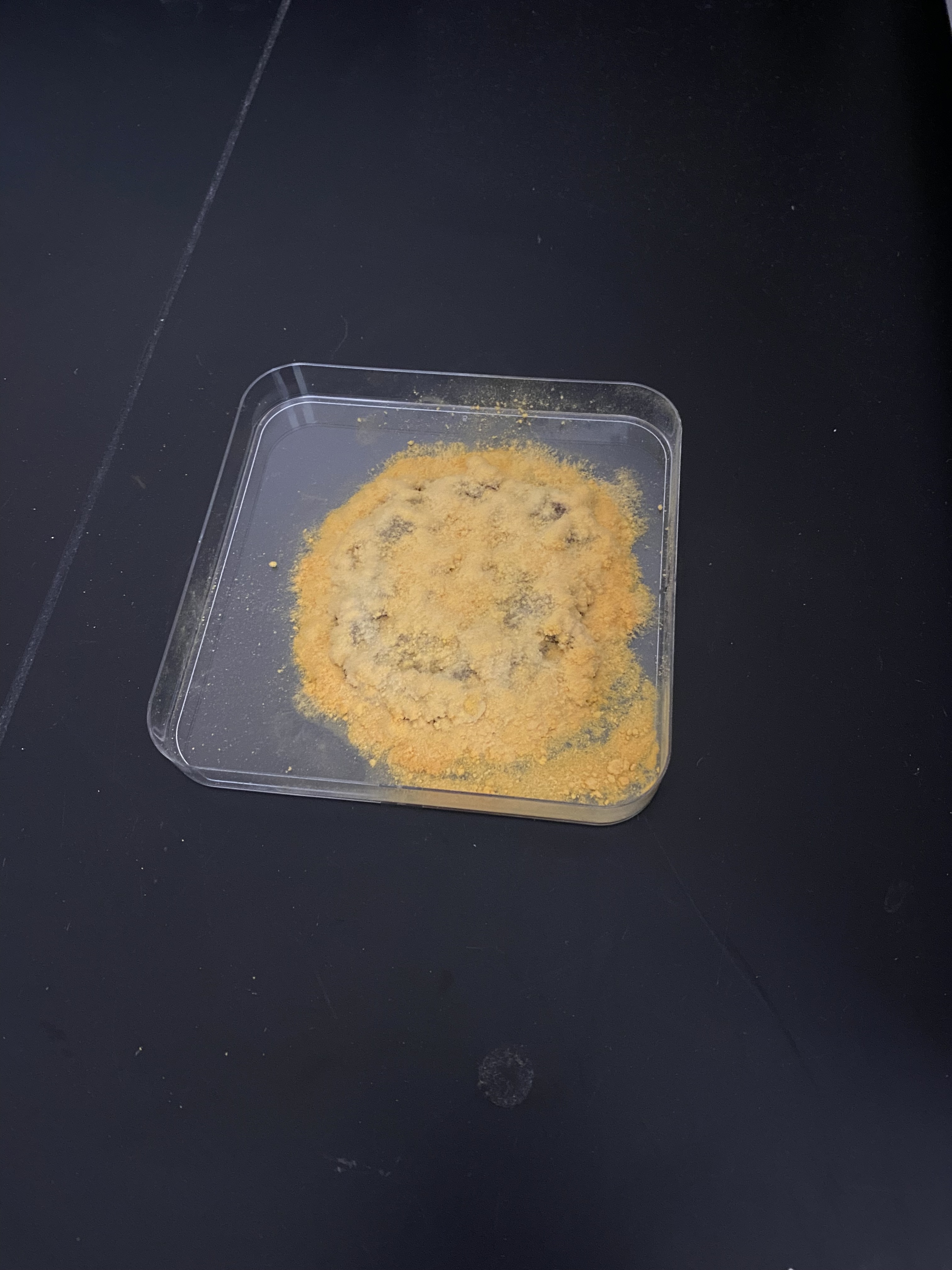

Supplement: Supplementary file 7 — Source data. [file 41564_2024_1799_MOESM7_ESM.zip › Fig4-sourcedata/2023-11-06_tomatopomace-NI copy.jpg]

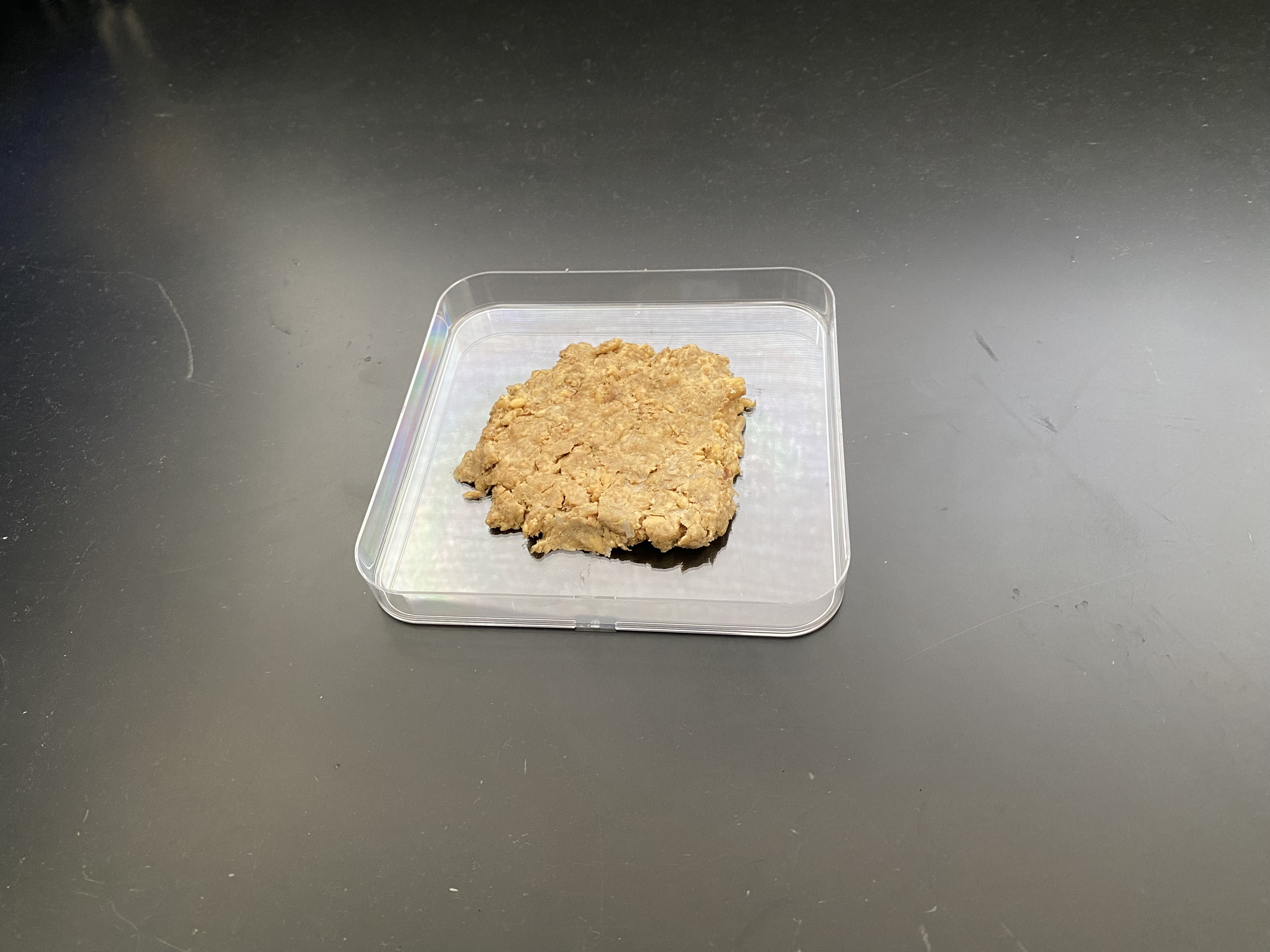

Supplement: Supplementary file 7 — Source data. [file 41564_2024_1799_MOESM7_ESM.zip › Fig4-sourcedata/2_oatmilkwaste copy.jpg]

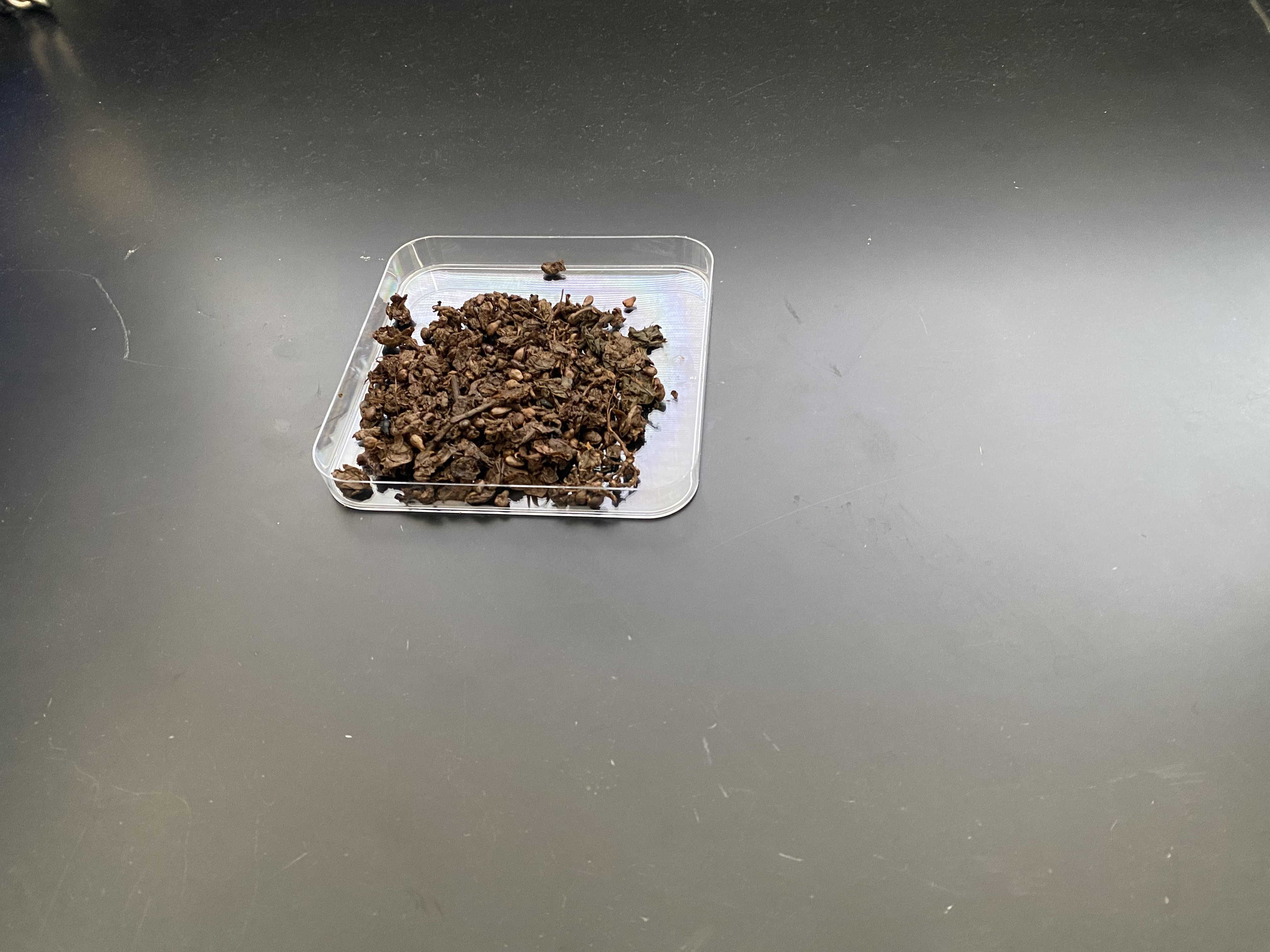

Supplement: Supplementary file 7 — Source data. [file 41564_2024_1799_MOESM7_ESM.zip › Fig4-sourcedata/10_grapepomace-NI copy.jpg]

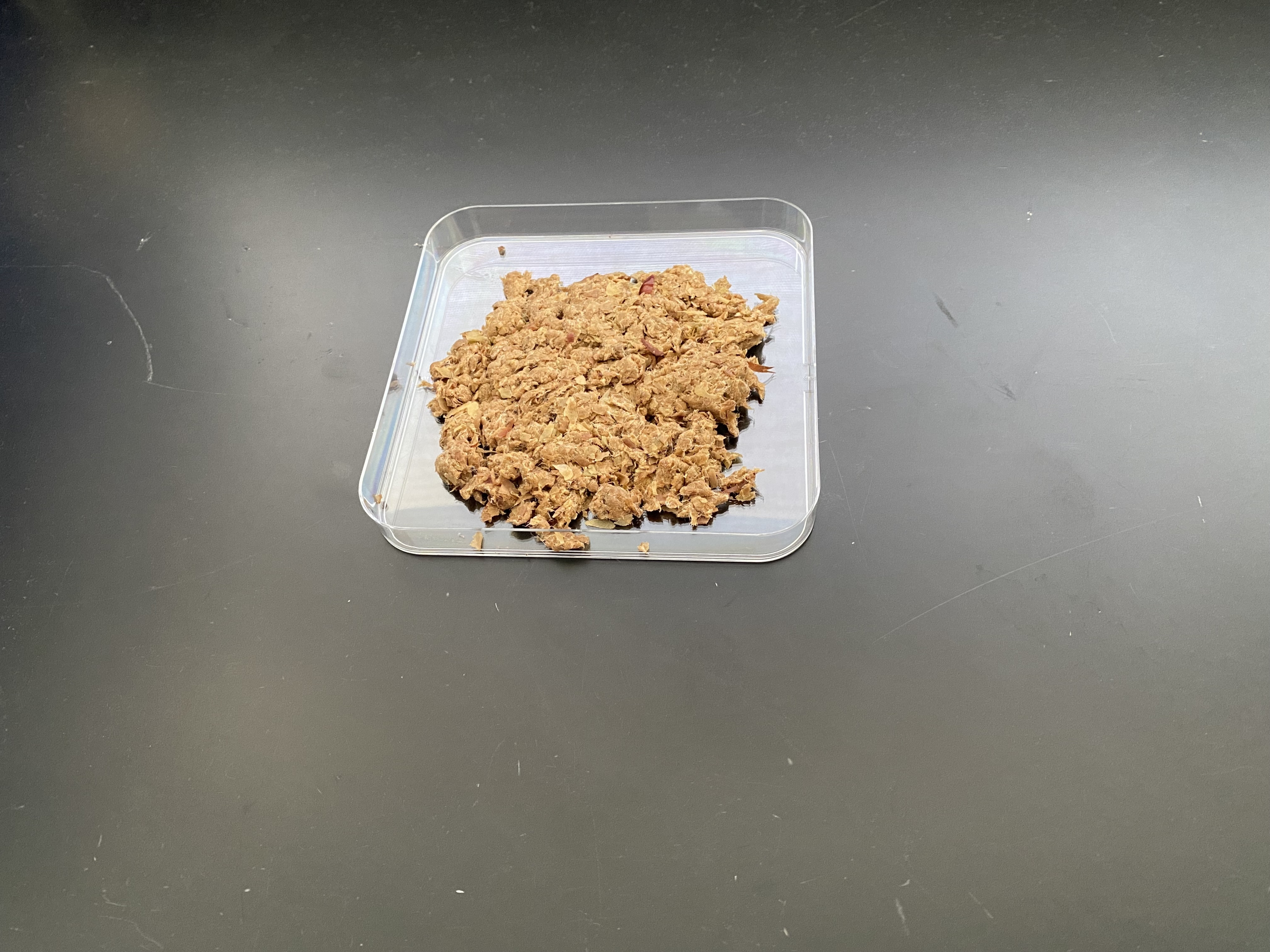

Supplement: Supplementary file 7 — Source data. [file 41564_2024_1799_MOESM7_ESM.zip › Fig4-sourcedata/8_applepomace copy.jpg]

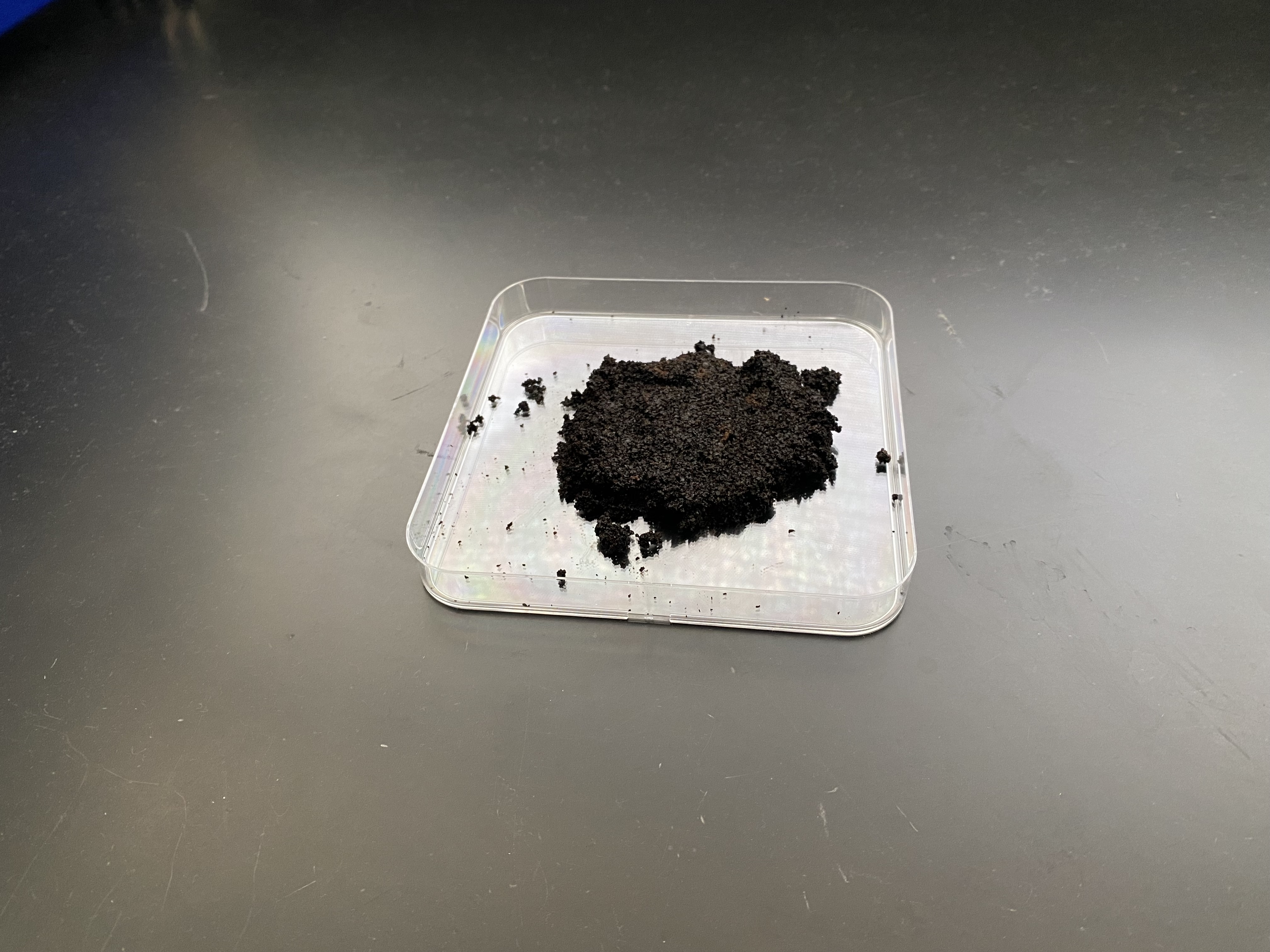

Supplement: Supplementary file 7 — Source data. [file 41564_2024_1799_MOESM7_ESM.zip › Fig4-sourcedata/1_coffegrounds copy.jpg]

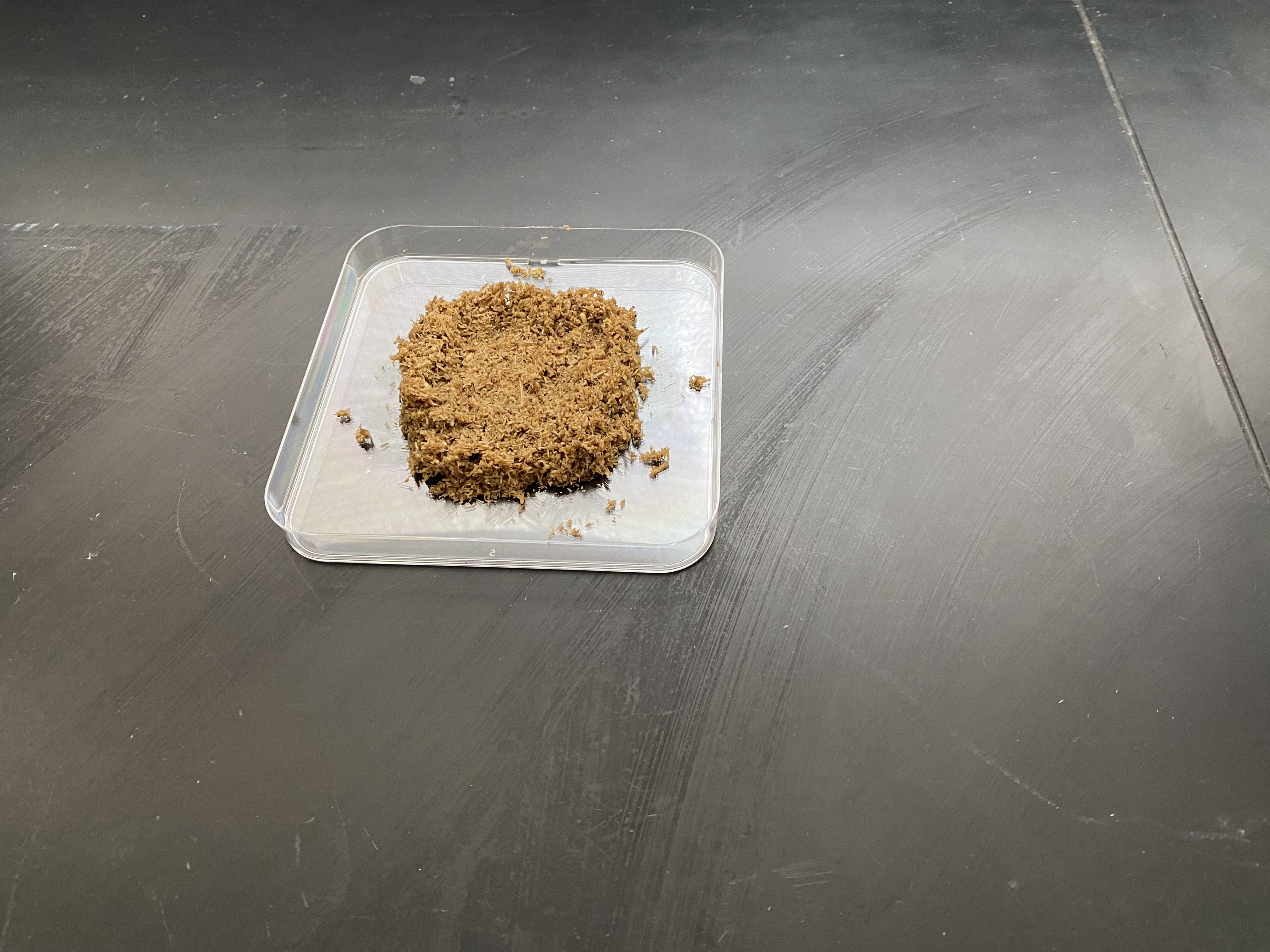

Supplement: Supplementary file 7 — Source data. [file 41564_2024_1799_MOESM7_ESM.zip › Fig4-sourcedata/11_maltrootlets copy.jpg]

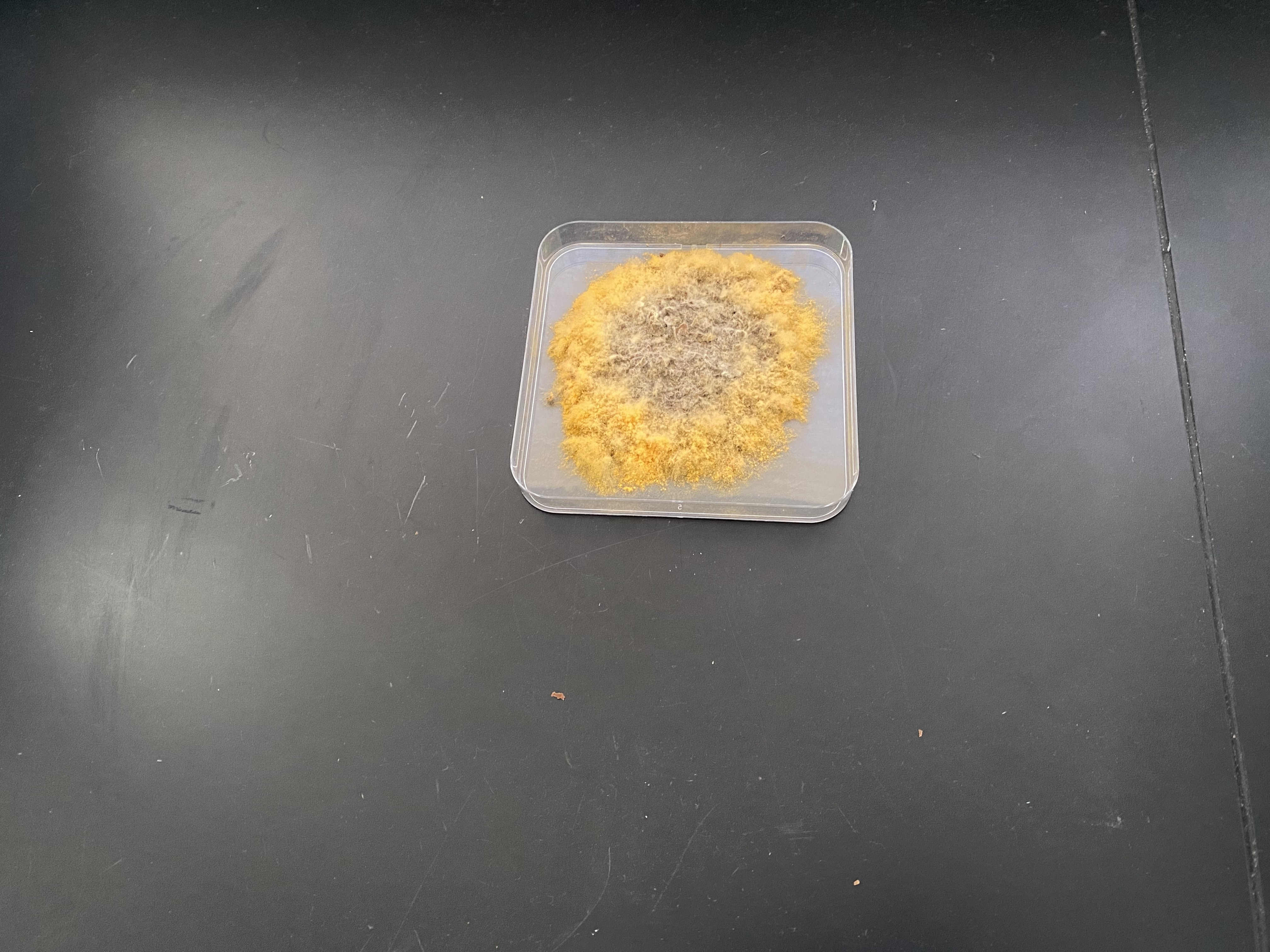

Supplement: Supplementary file 7 — Source data. [file 41564_2024_1799_MOESM7_ESM.zip › Fig4-sourcedata/2023-10-23_almondskins-NI copy.jpg]

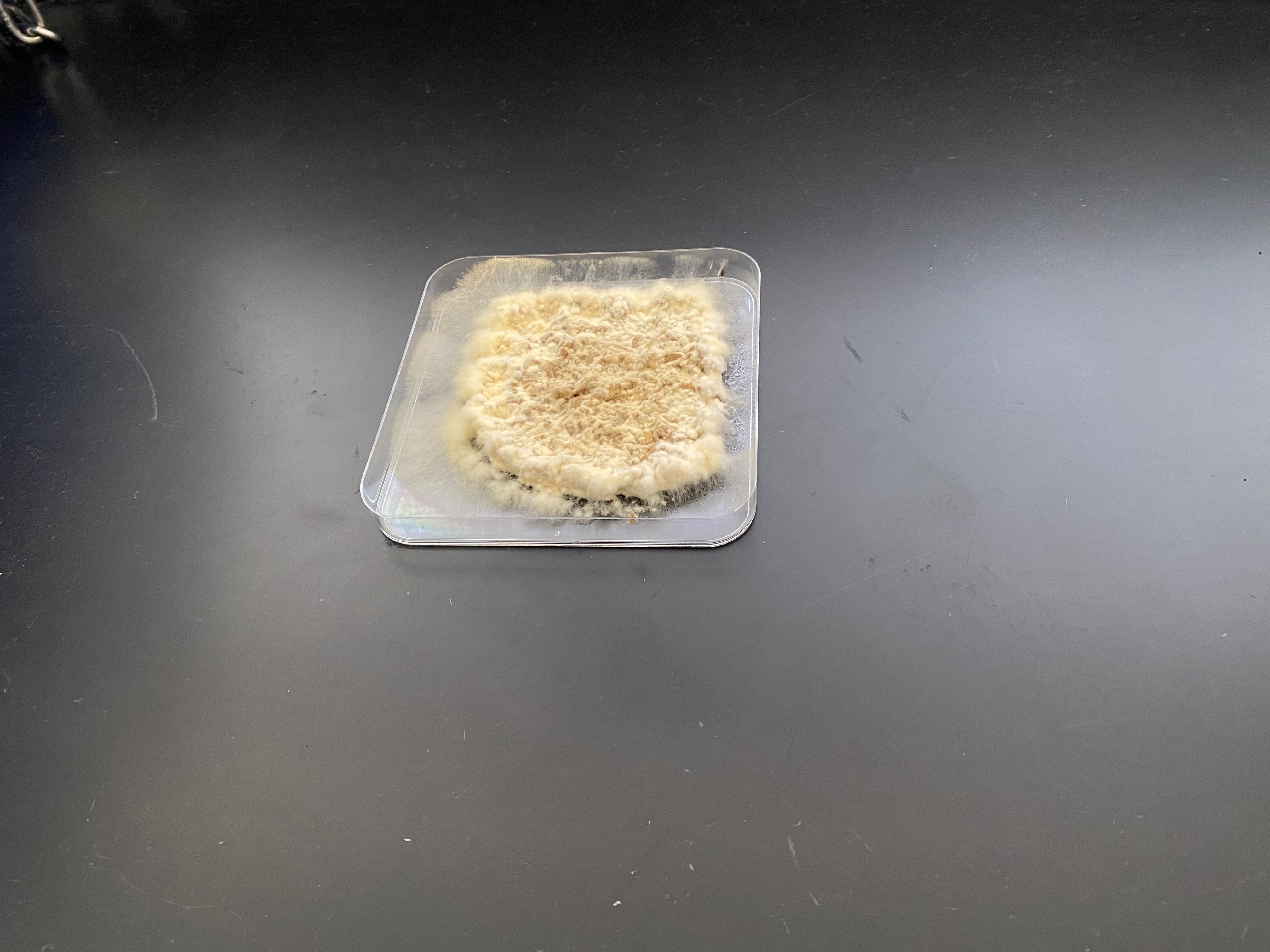

Supplement: Supplementary file 7 — Source data. [file 41564_2024_1799_MOESM7_ESM.zip › Fig4-sourcedata/2_oatmilkwaste-NI copy.jpg]

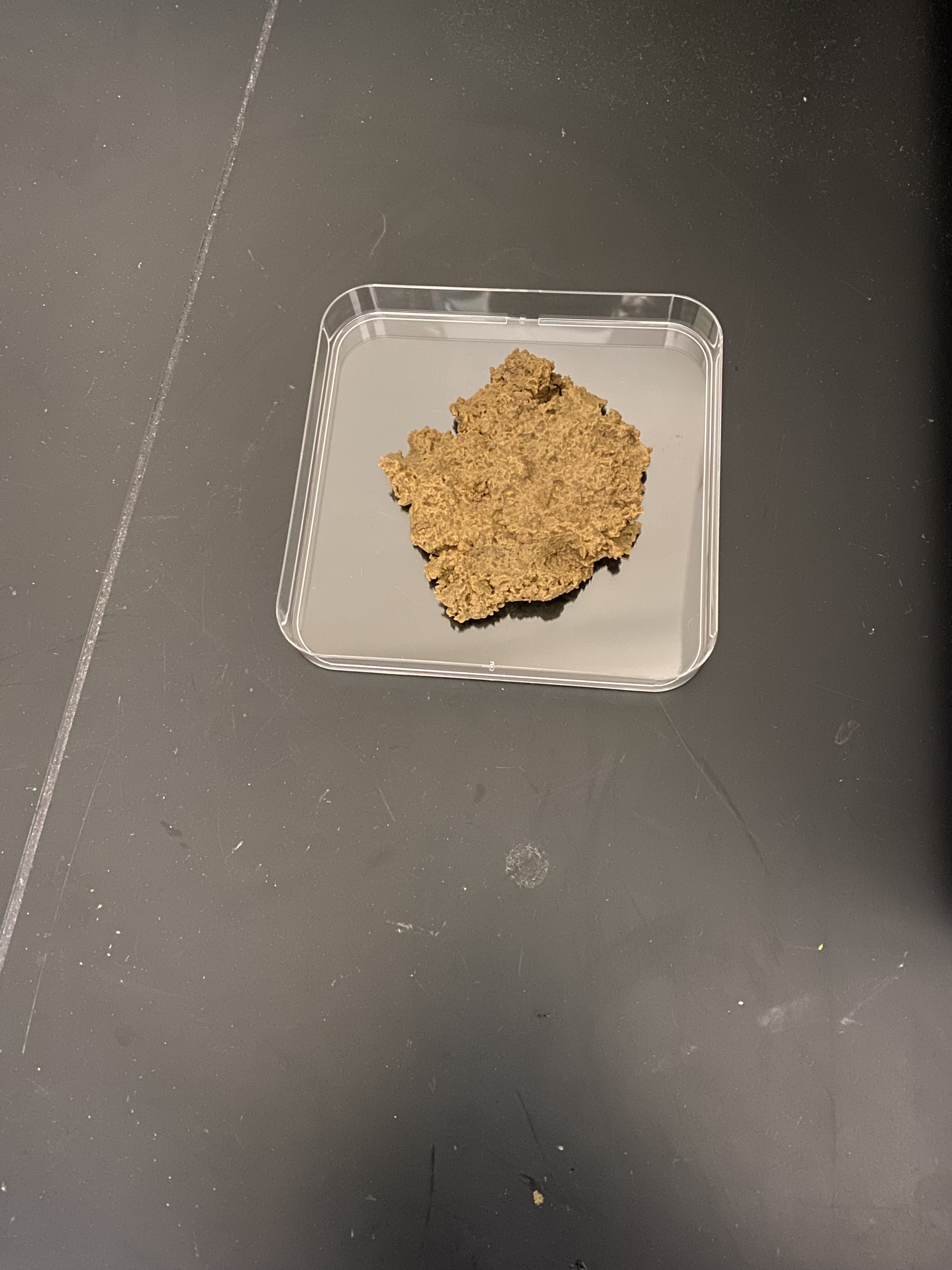

Supplement: Supplementary file 7 — Source data. [file 41564_2024_1799_MOESM7_ESM.zip › Fig4-sourcedata/Olivepomace copy.jpg]

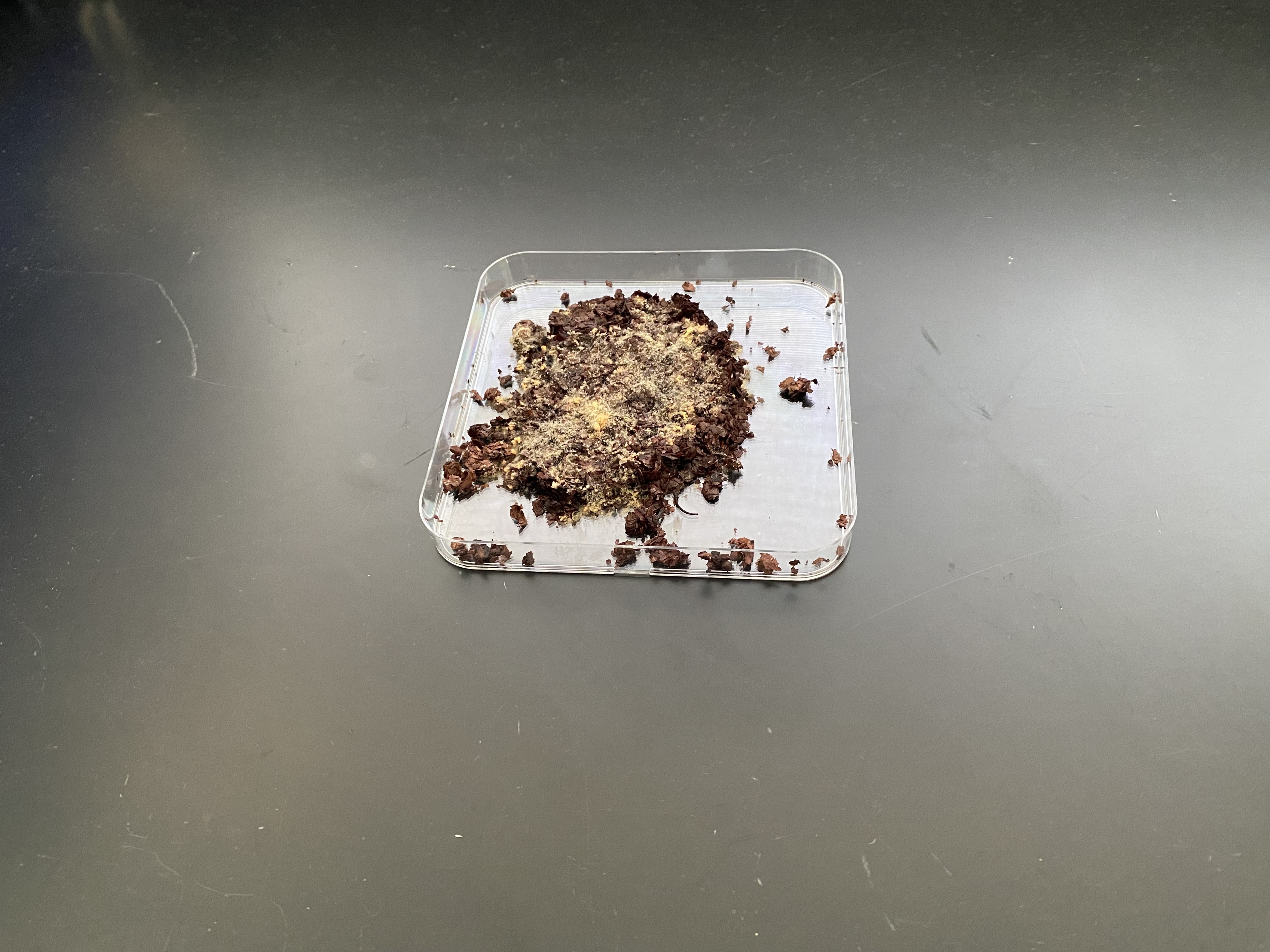

Supplement: Supplementary file 7 — Source data. [file 41564_2024_1799_MOESM7_ESM.zip › Fig4-sourcedata/7_hazelnutskin-NI copy.jpg]

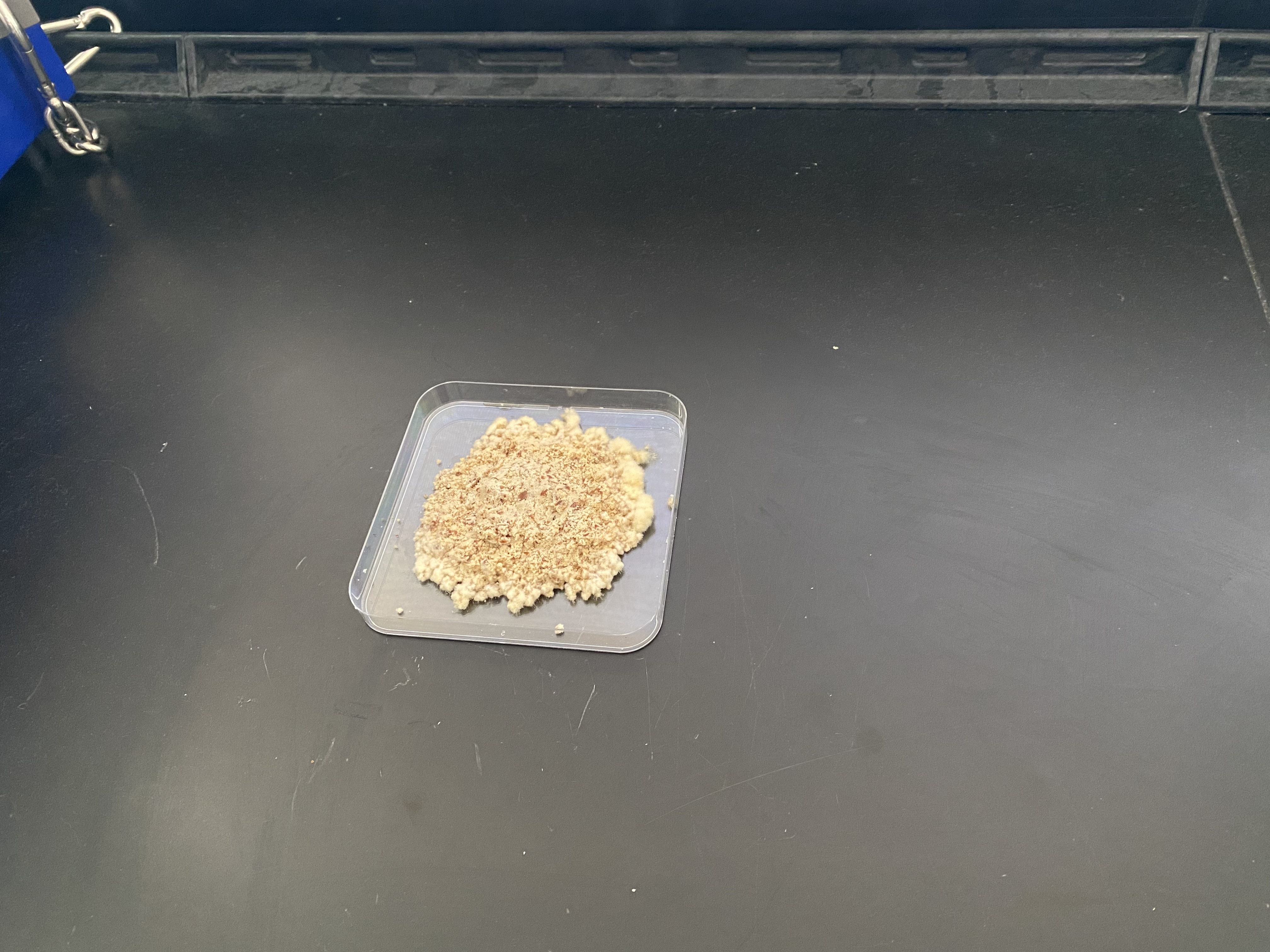

Supplement: Supplementary file 7 — Source data. [file 41564_2024_1799_MOESM7_ESM.zip › Fig4-sourcedata/2023-10-23_almondmilkwaste-NI copy.jpg]

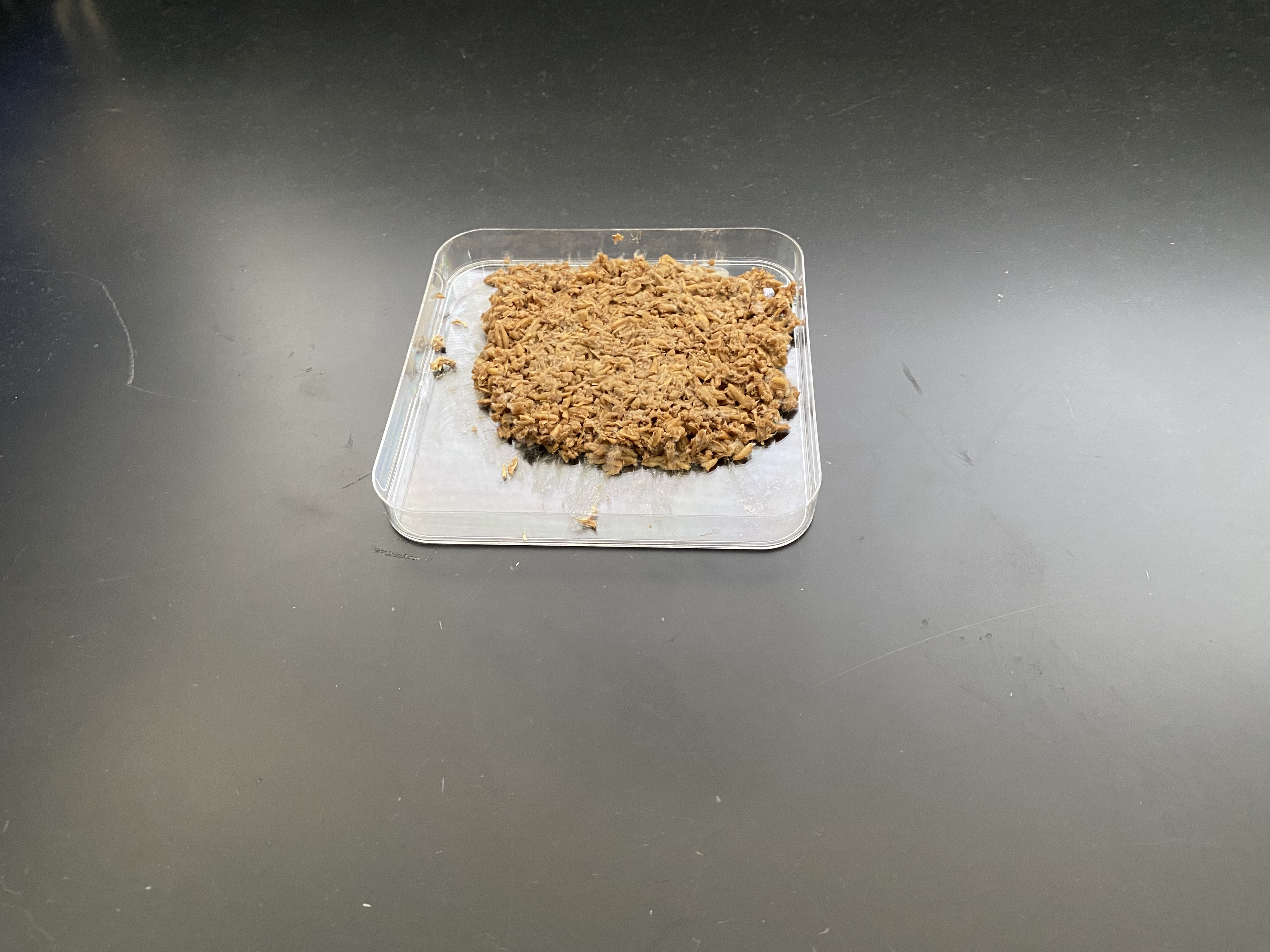

Supplement: Supplementary file 7 — Source data. [file 41564_2024_1799_MOESM7_ESM.zip › Fig4-sourcedata/4_spentgrain-NI copy.jpg]

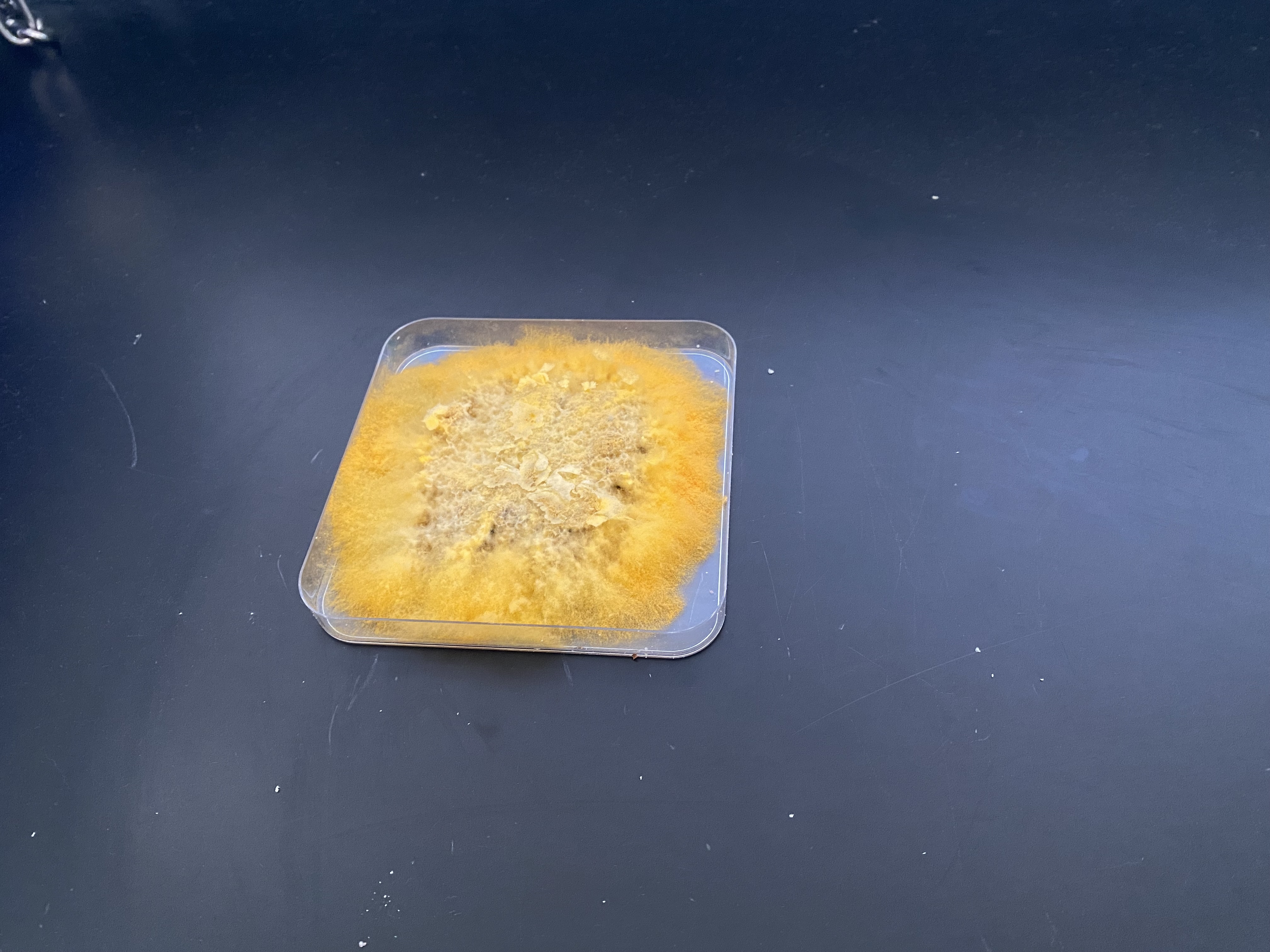

Supplement: Supplementary file 7 — Source data. [file 41564_2024_1799_MOESM7_ESM.zip › Fig4-sourcedata/2023-10-23_tigernutmilkwaste-NI copy.jpg]

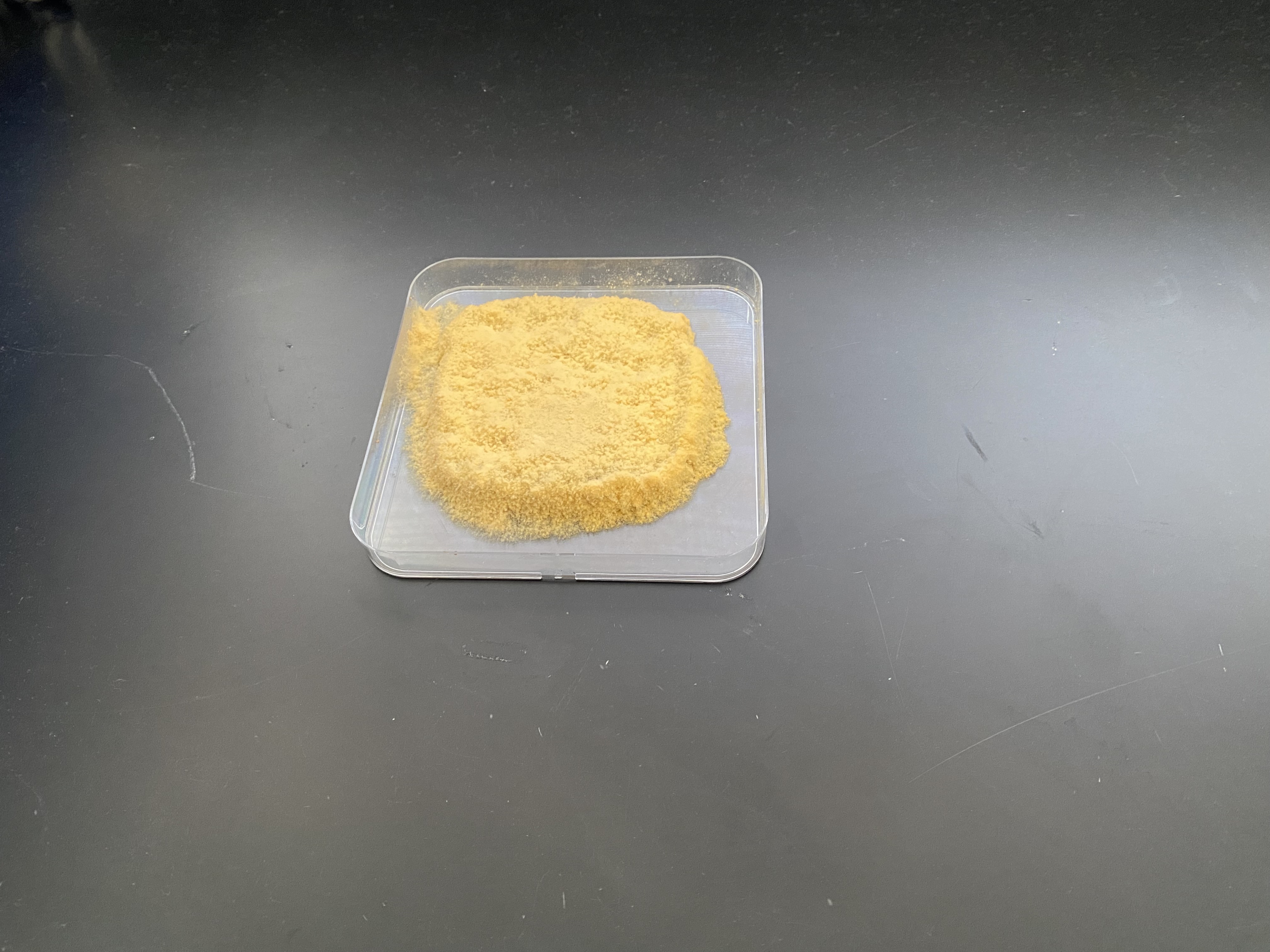

Supplement: Supplementary file 7 — Source data. [file 41564_2024_1799_MOESM7_ESM.zip › Fig4-sourcedata/5_pumpkinseedpresscake-NI copy.jpg]

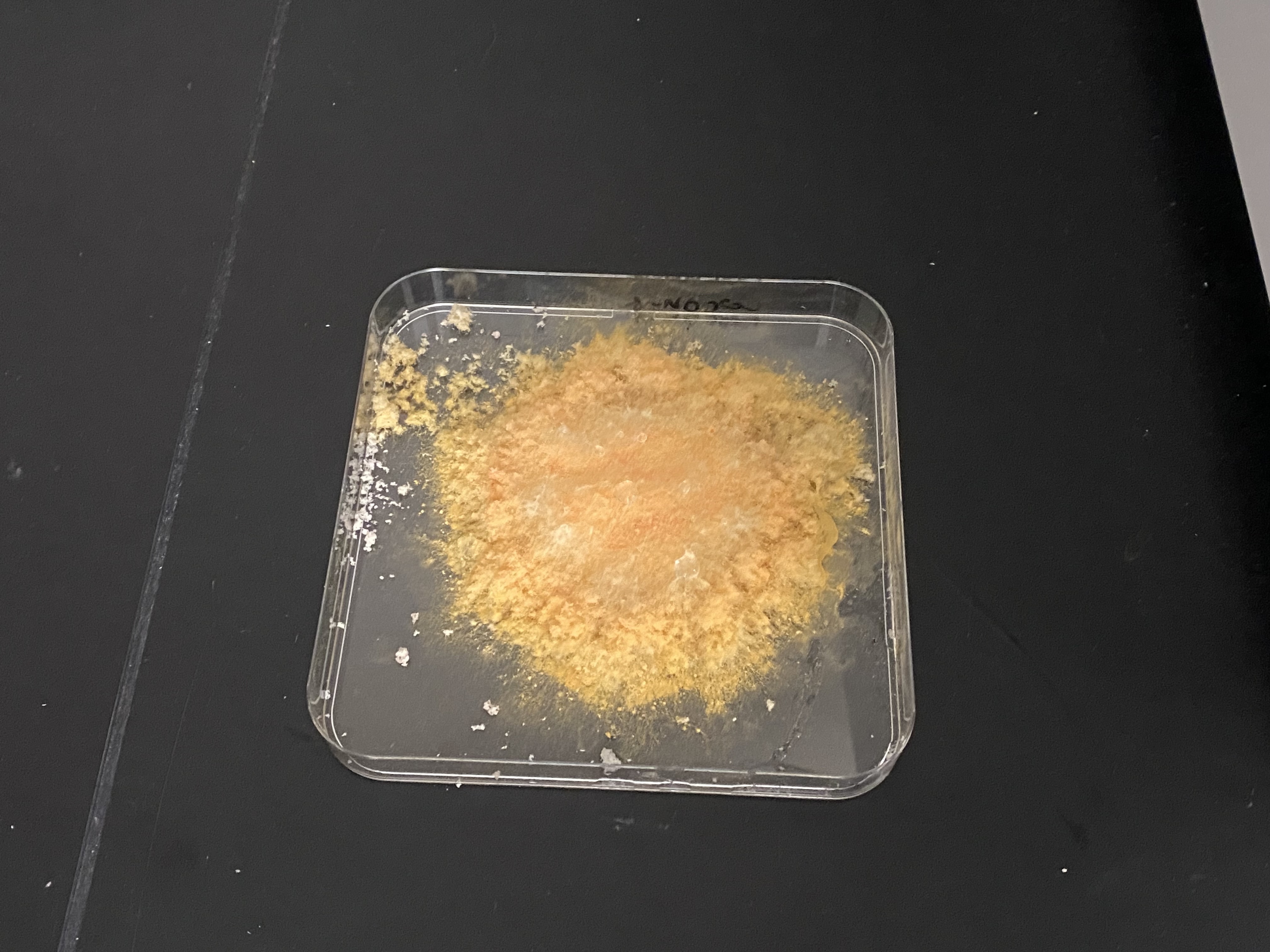

Supplement: Supplementary file 7 — Source data. [file 41564_2024_1799_MOESM7_ESM.zip › Fig4-sourcedata/2023-11-06_coconutmilkwaste-NI copy.jpg]

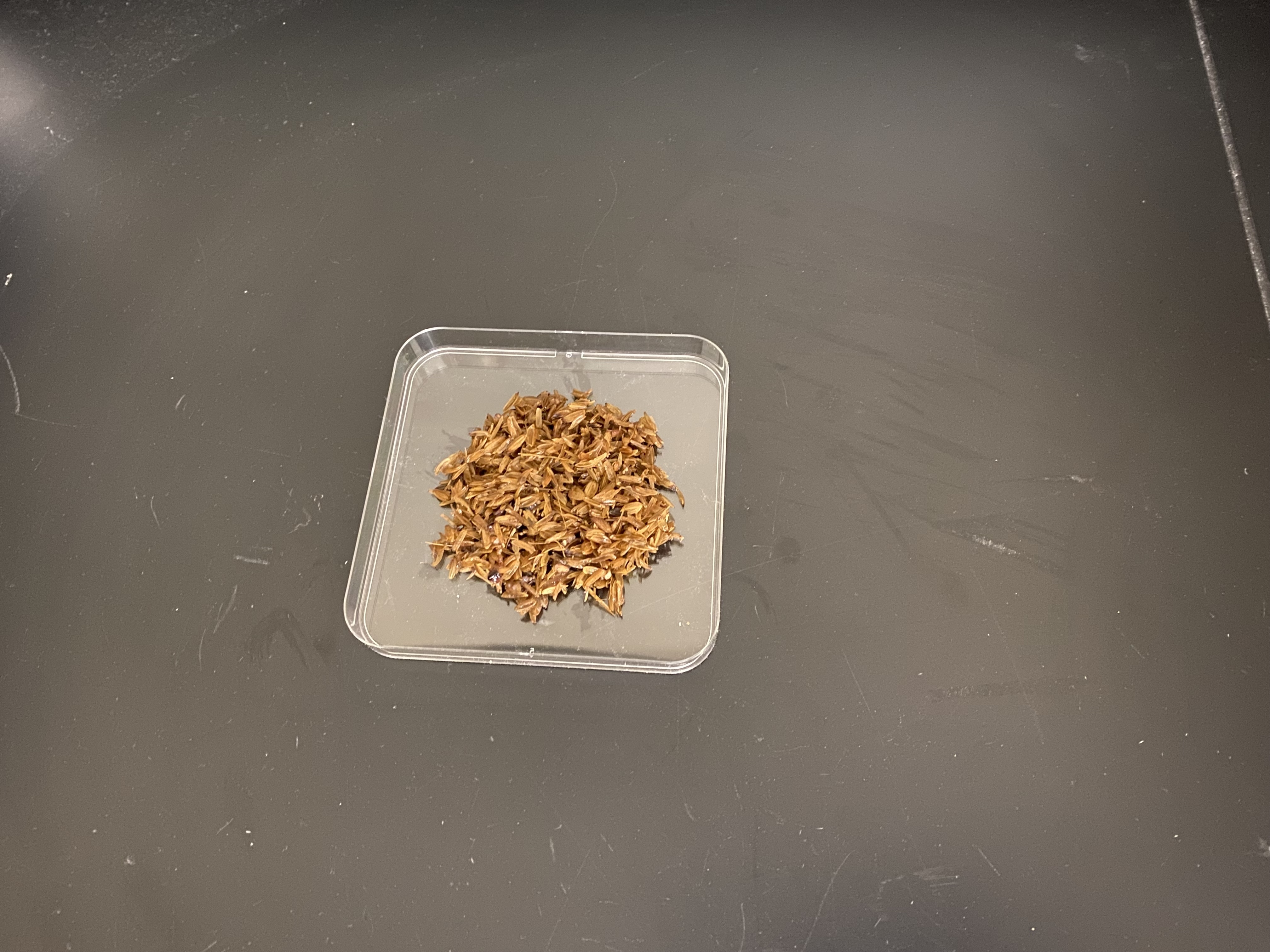

Supplement: Supplementary file 7 — Source data. [file 41564_2024_1799_MOESM7_ESM.zip › Fig4-sourcedata/2023-11-06_ricehulls copy.jpg]

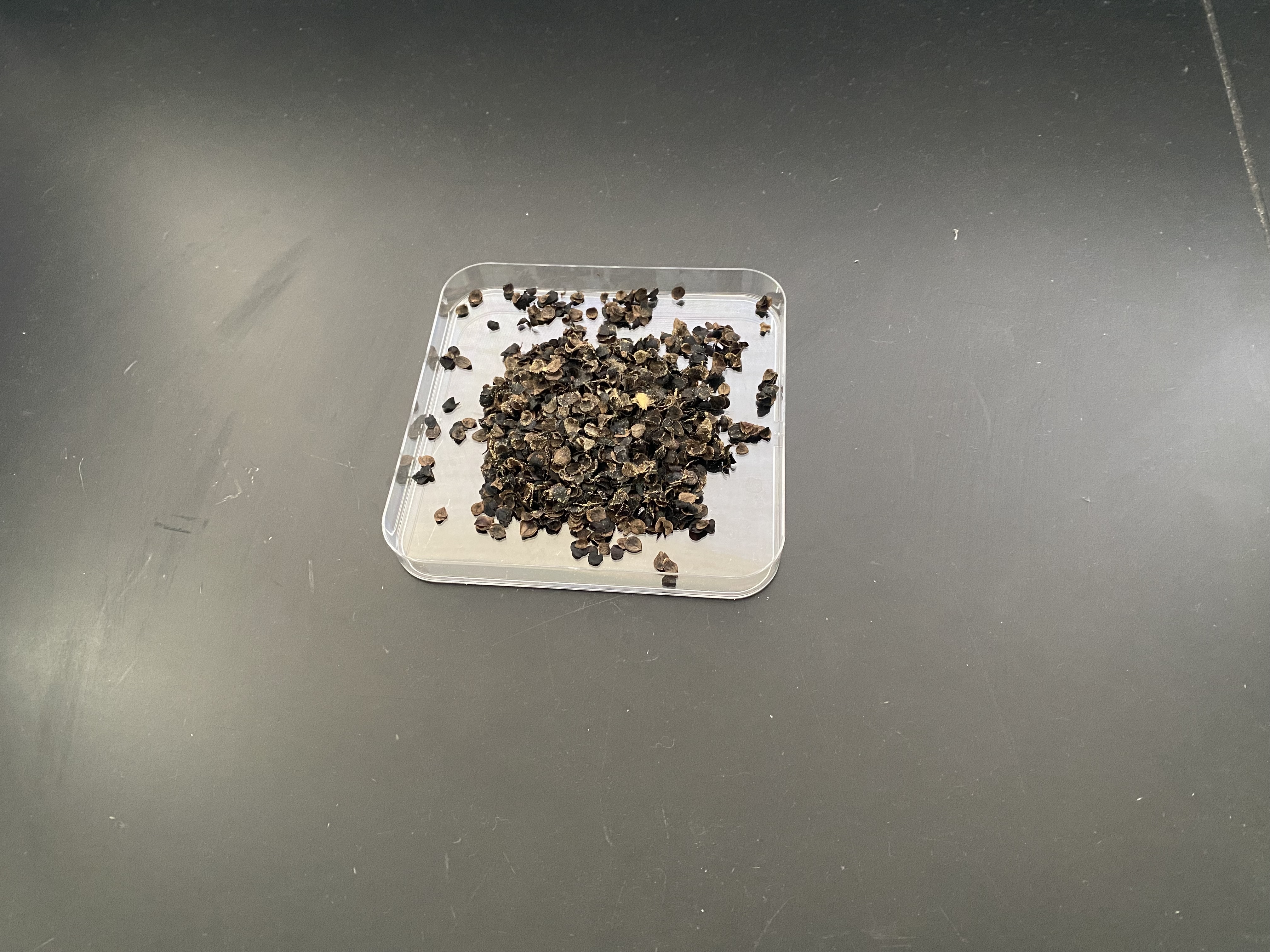

Supplement: Supplementary file 7 — Source data. [file 41564_2024_1799_MOESM7_ESM.zip › Fig4-sourcedata/2023-10-23_buckwheathulls_NI copy.jpg]

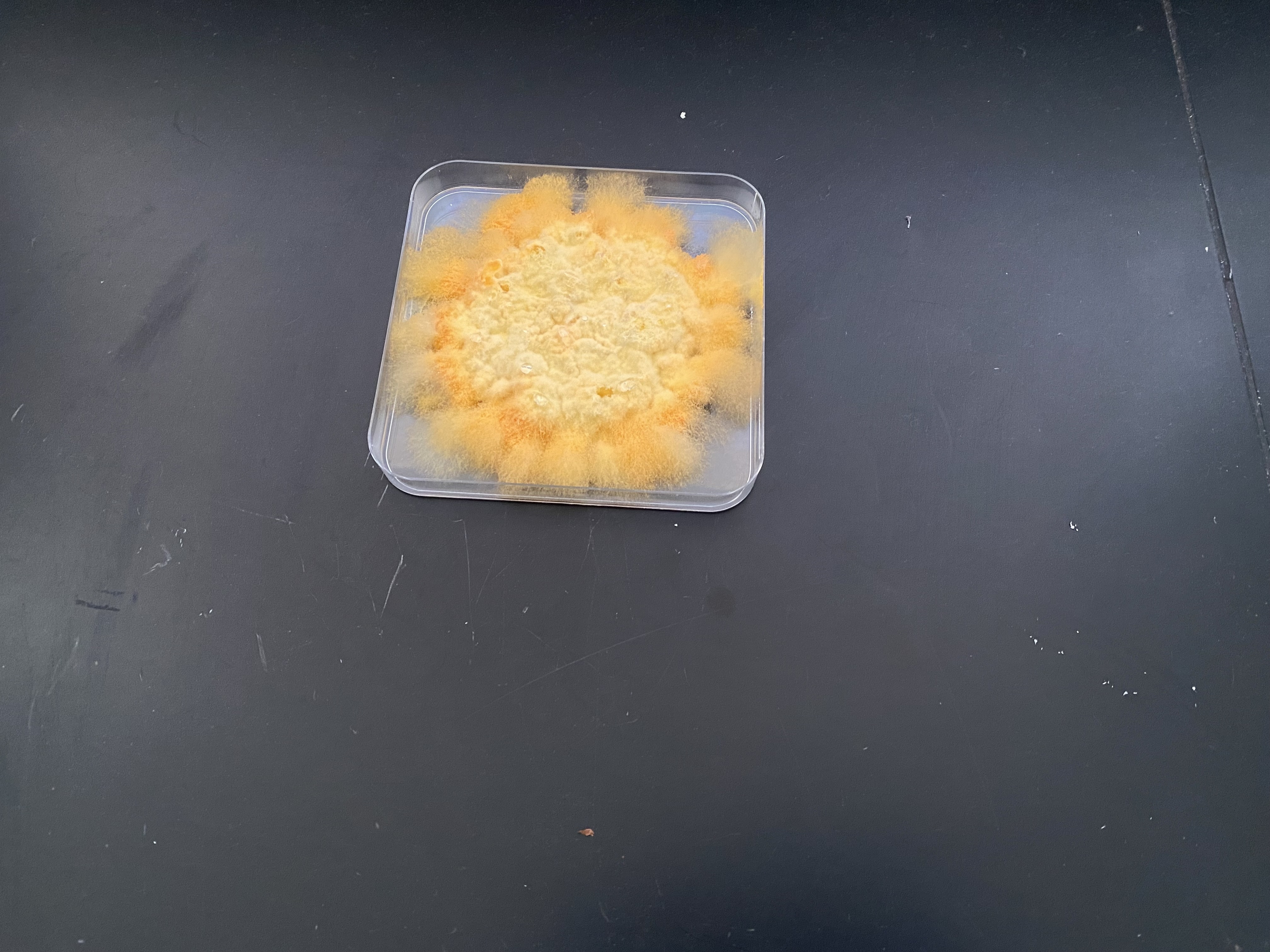

Supplement: Supplementary file 7 — Source data. [file 41564_2024_1799_MOESM7_ESM.zip › Fig4-sourcedata/2023-10-23_cashewmilkwaste-NI copy.jpg]

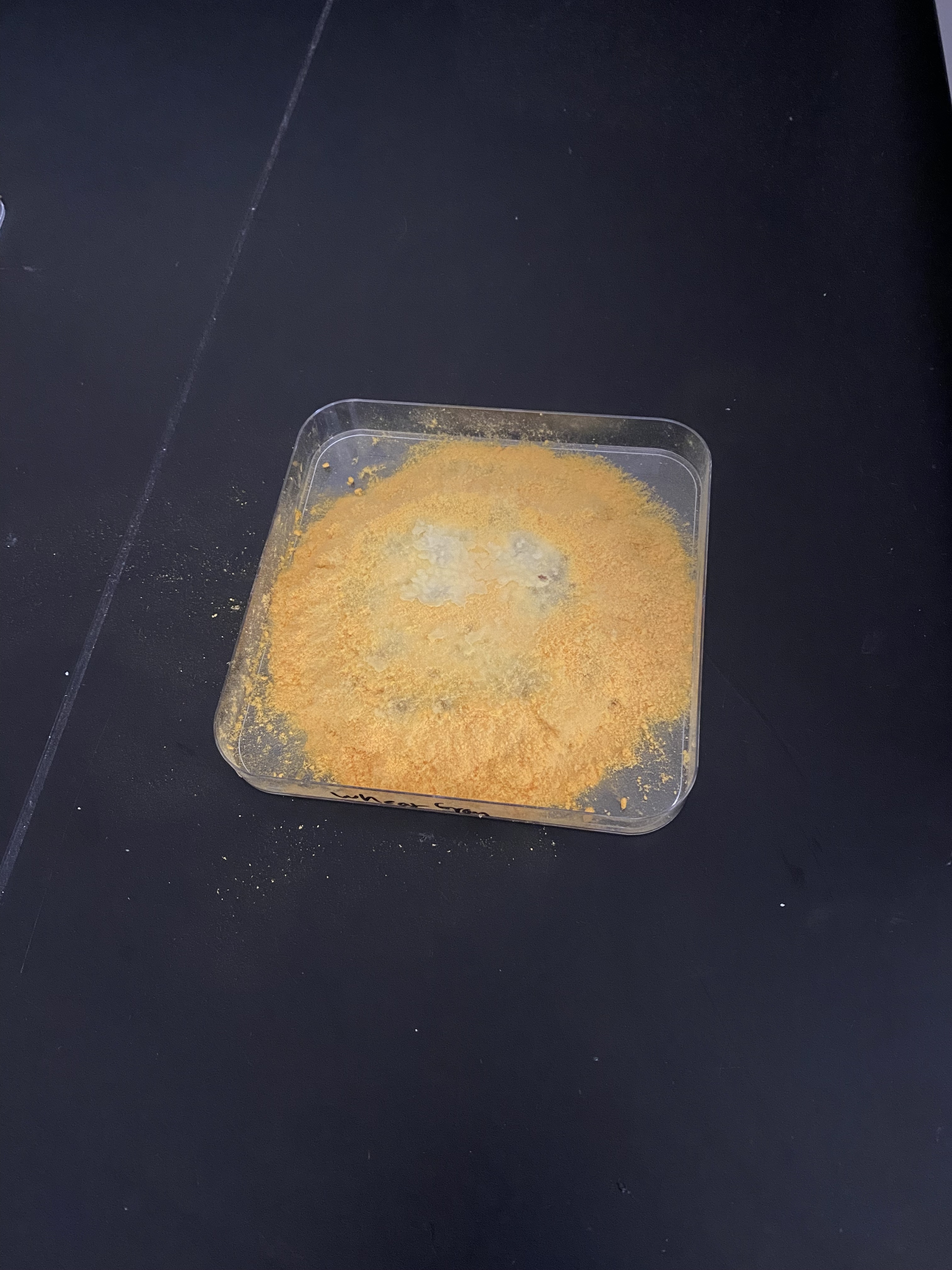

Supplement: Supplementary file 7 — Source data. [file 41564_2024_1799_MOESM7_ESM.zip › Fig4-sourcedata/2023-11-06_wheatbran-NI copy.jpg]

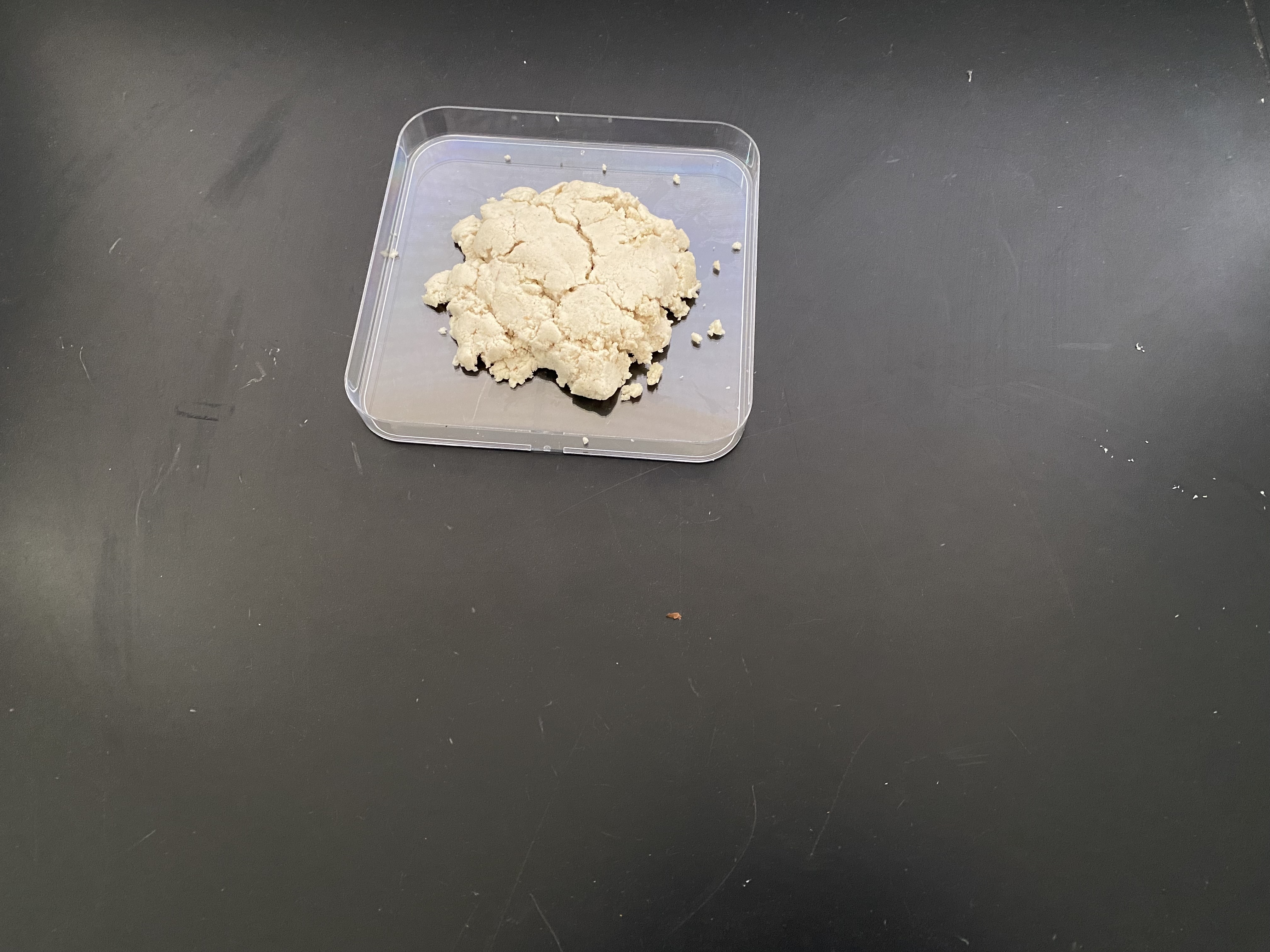

Supplement: Supplementary file 7 — Source data. [file 41564_2024_1799_MOESM7_ESM.zip › Fig4-sourcedata/2023-10-23_cashewmilkwaste copy.jpg]

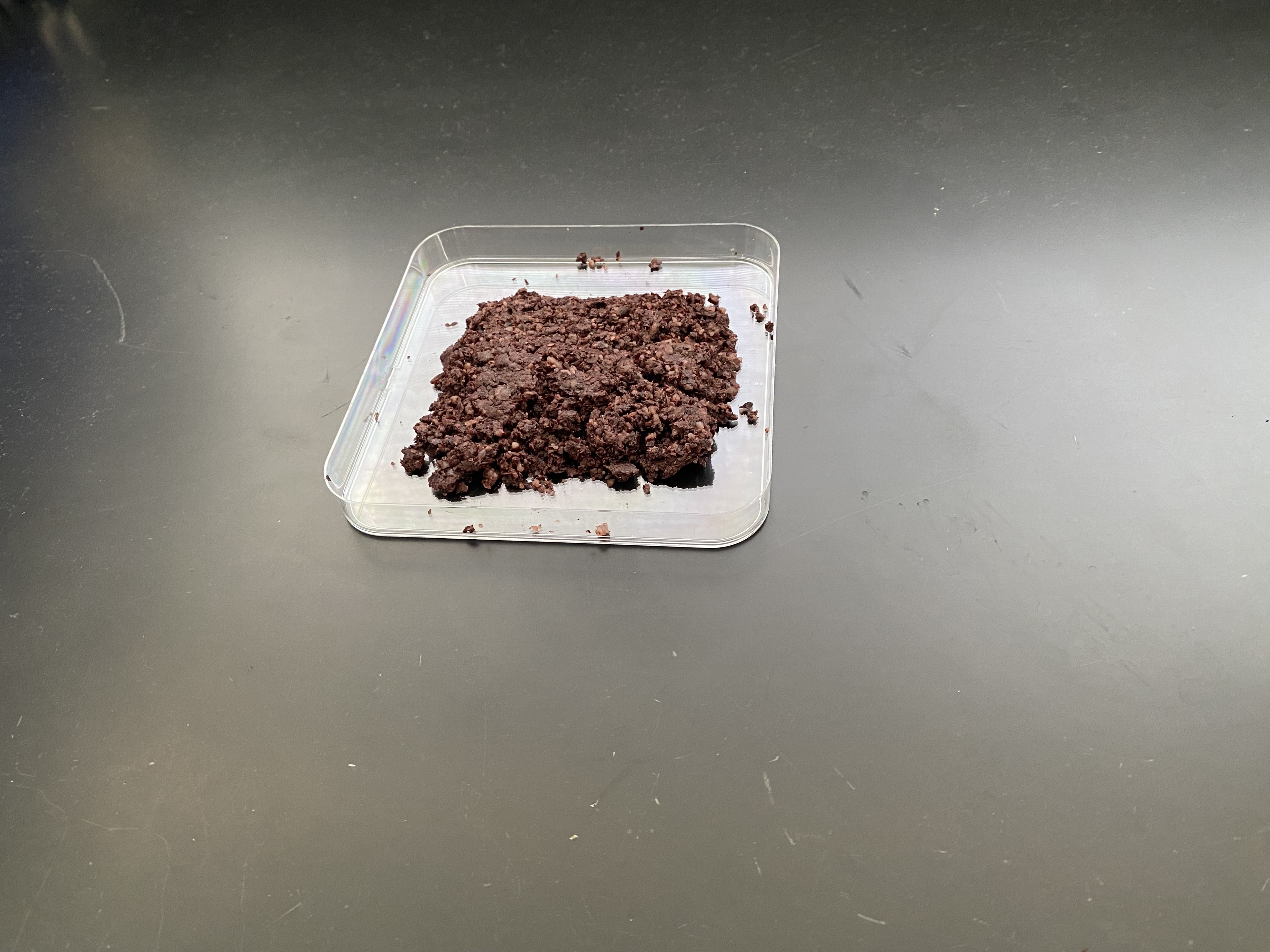

Supplement: Supplementary file 7 — Source data. [file 41564_2024_1799_MOESM7_ESM.zip › Fig4-sourcedata/3_ricemilkwaste copy.jpg]

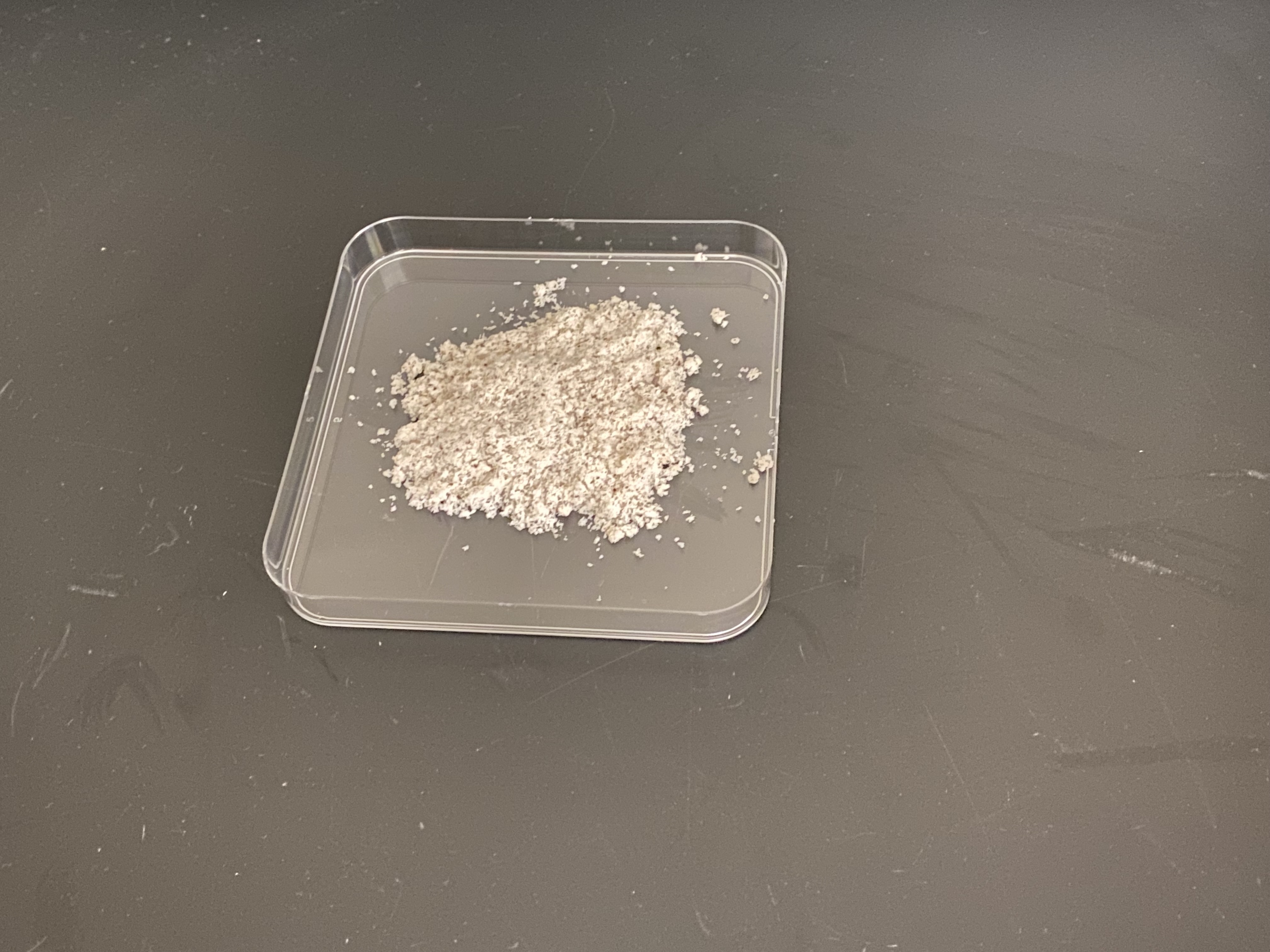

Supplement: Supplementary file 7 — Source data. [file 41564_2024_1799_MOESM7_ESM.zip › Fig4-sourcedata/2023-11-06_coconutmilkwaste copy.jpg]

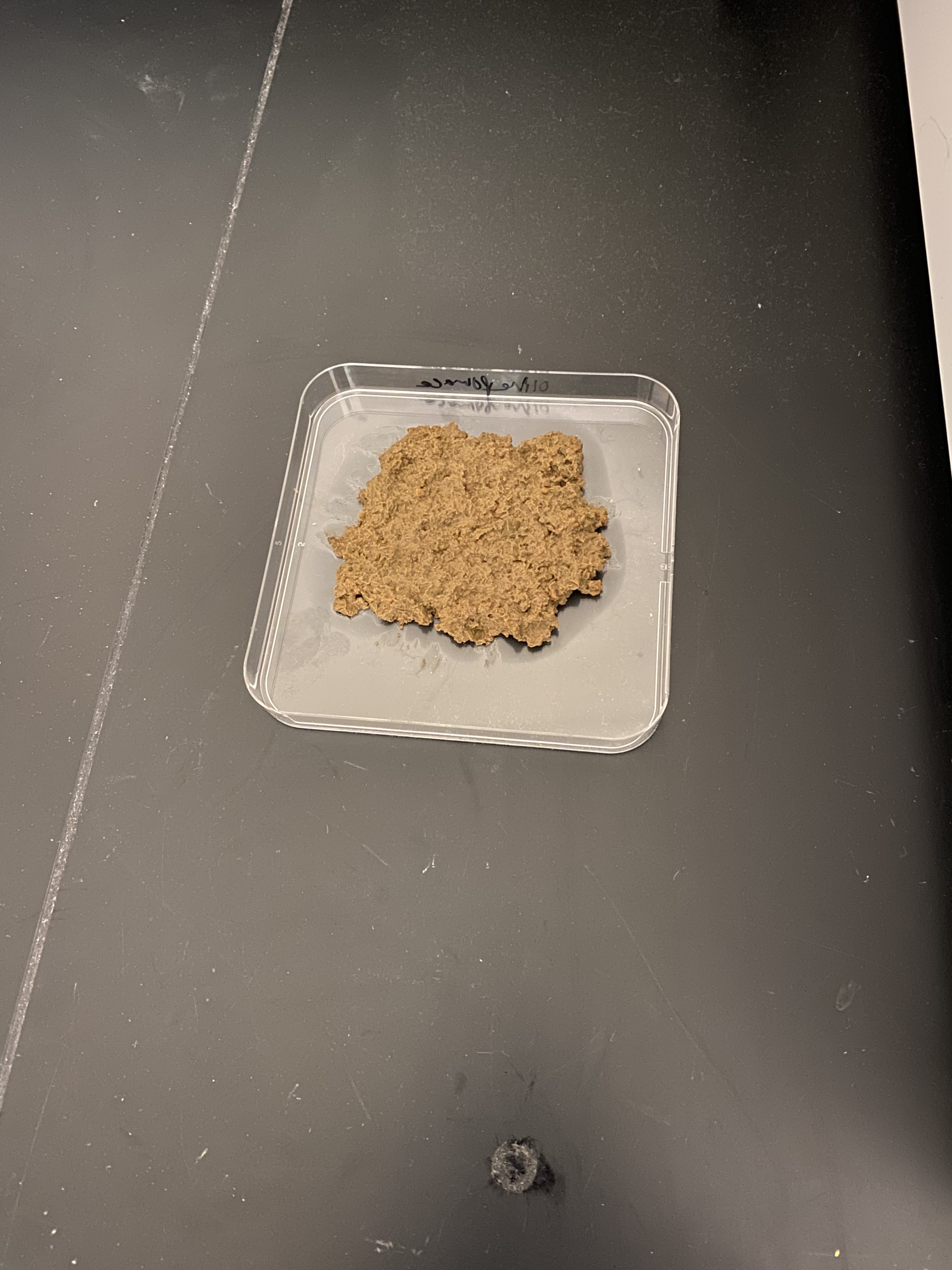

Supplement: Supplementary file 7 — Source data. [file 41564_2024_1799_MOESM7_ESM.zip › Fig4-sourcedata/Olivepomace-NI copy.jpg]

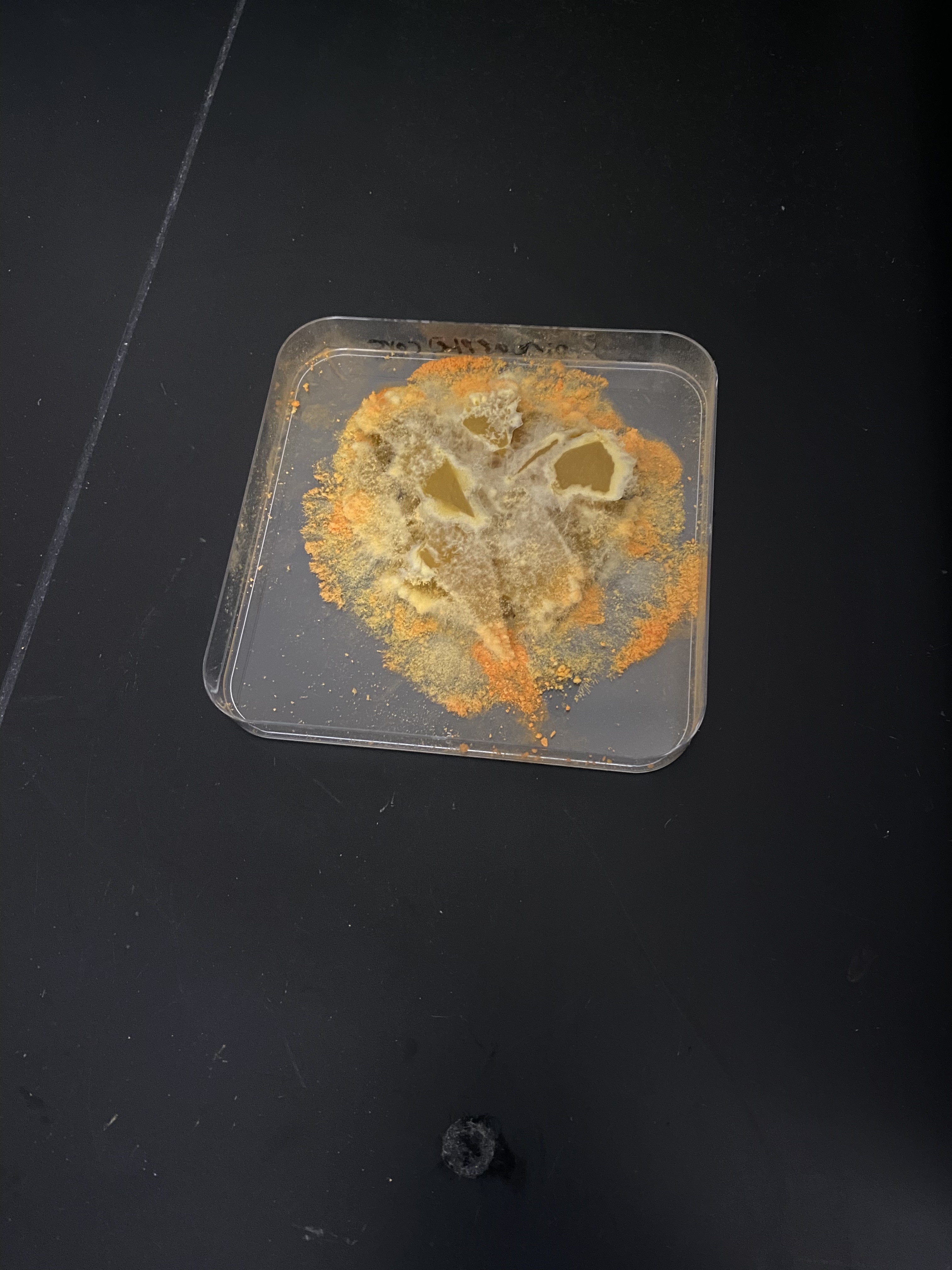

Supplement: Supplementary file 7 — Source data. [file 41564_2024_1799_MOESM7_ESM.zip › Fig4-sourcedata/2023-11-06_pineapplecore-NI copy.jpg]

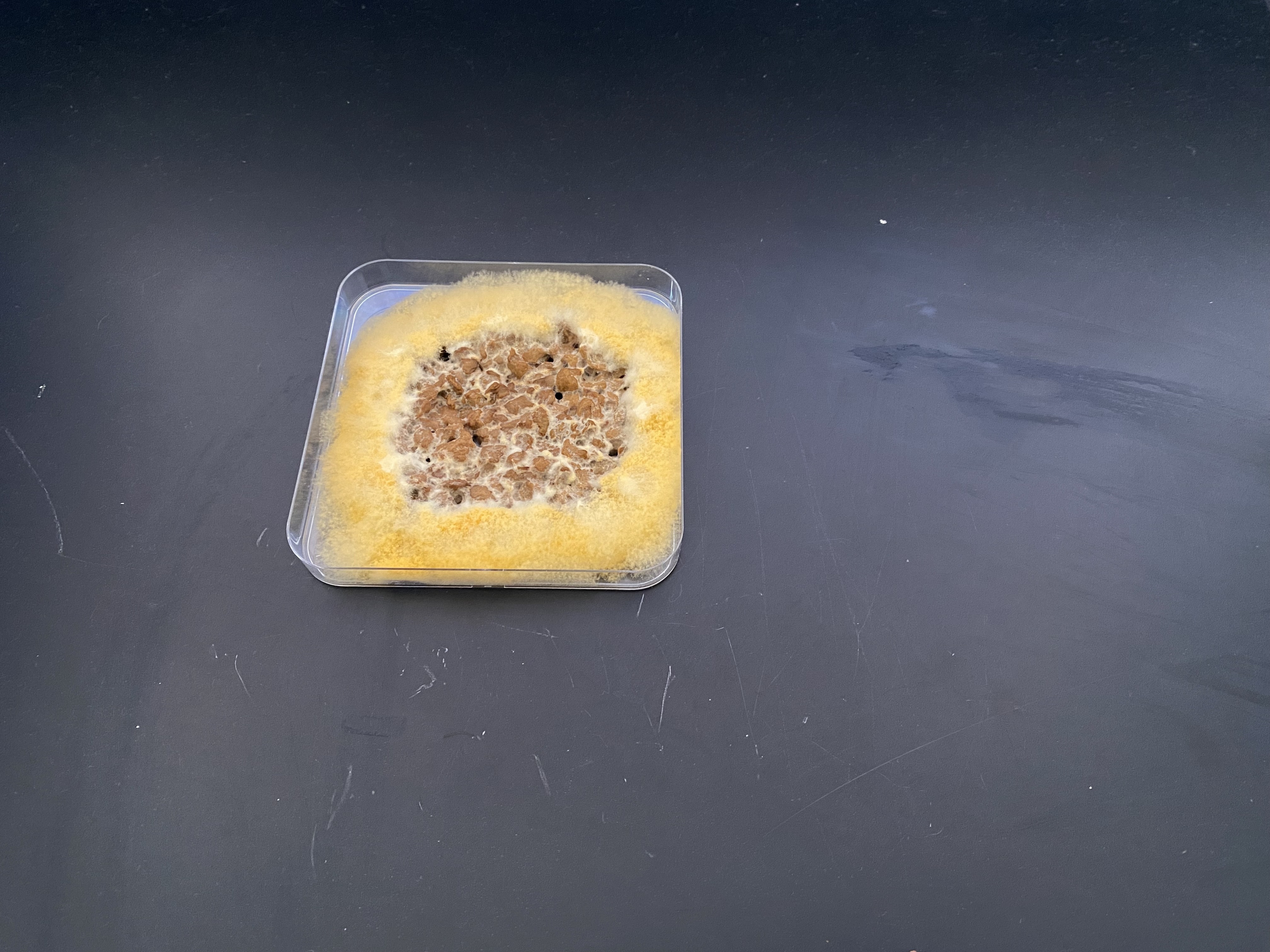

Supplement: Supplementary file 7 — Source data. [file 41564_2024_1799_MOESM7_ESM.zip › Fig4-sourcedata/2023-10-23_sunflowerseedTVP-NI copy.jpg]

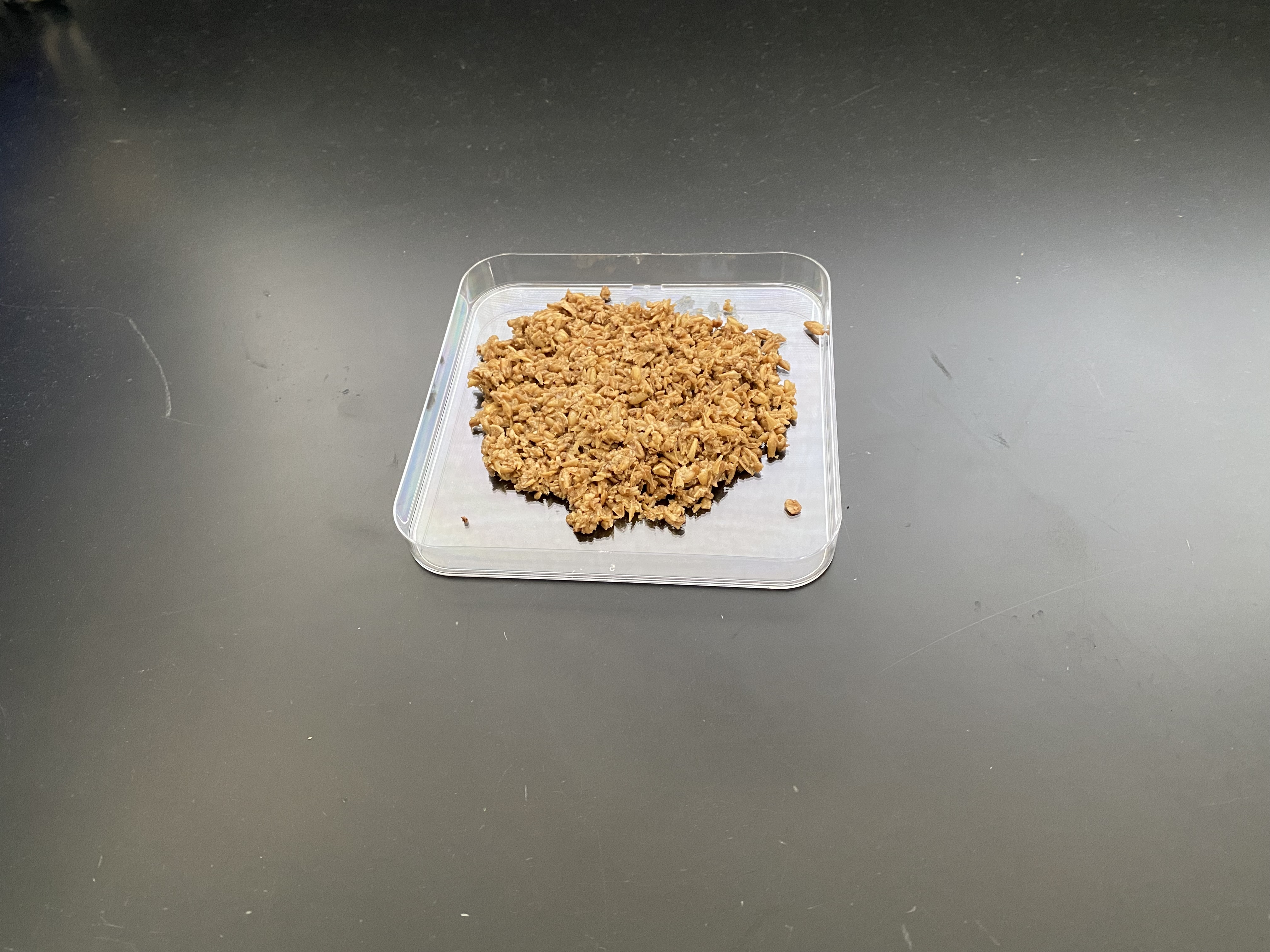

Supplement: Supplementary file 7 — Source data. [file 41564_2024_1799_MOESM7_ESM.zip › Fig4-sourcedata/4_spentgrain copy.jpg]

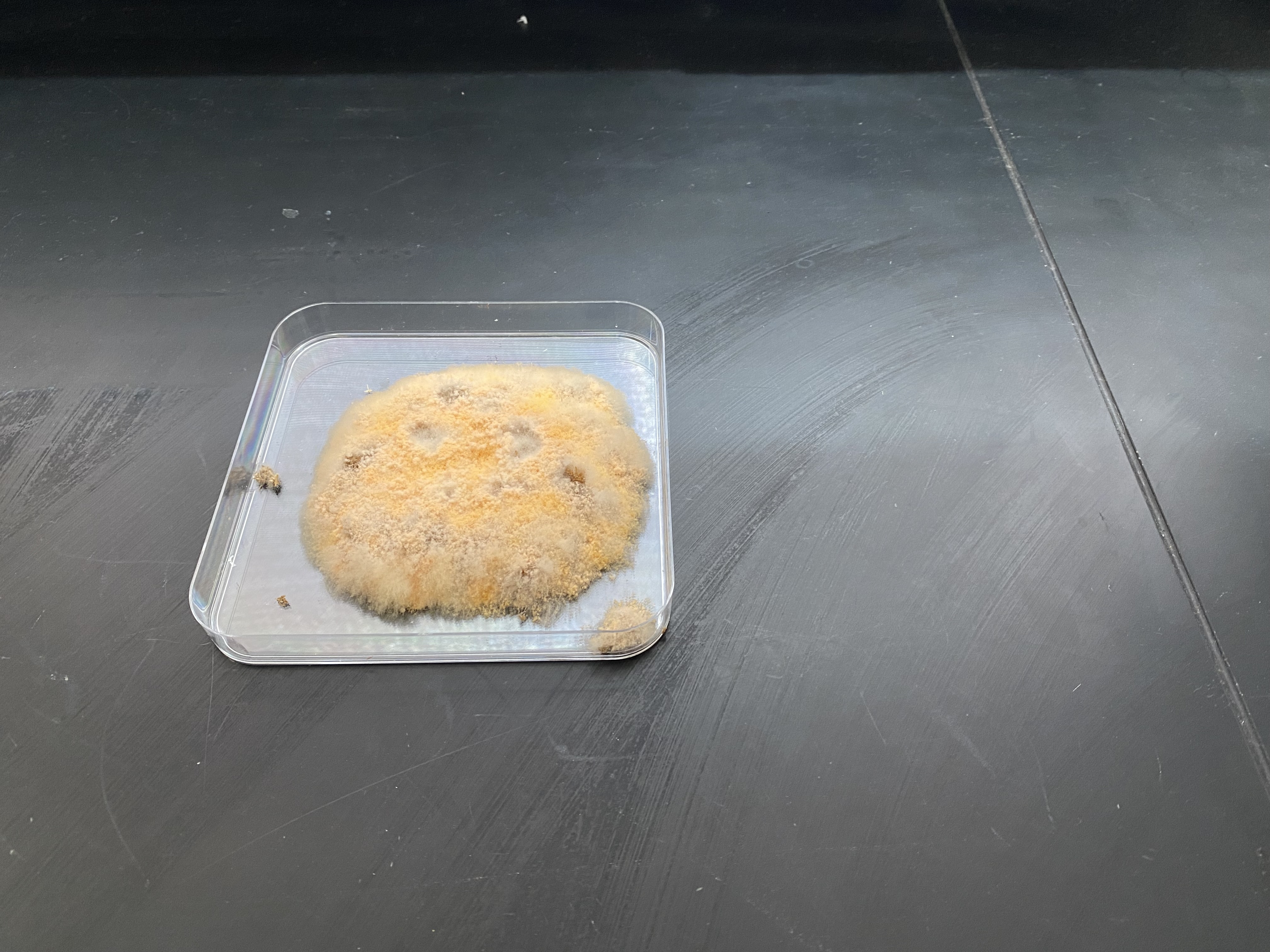

Supplement: Supplementary file 7 — Source data. [file 41564_2024_1799_MOESM7_ESM.zip › Fig4-sourcedata/11_maltrootlets-NI-1 copy.jpg]

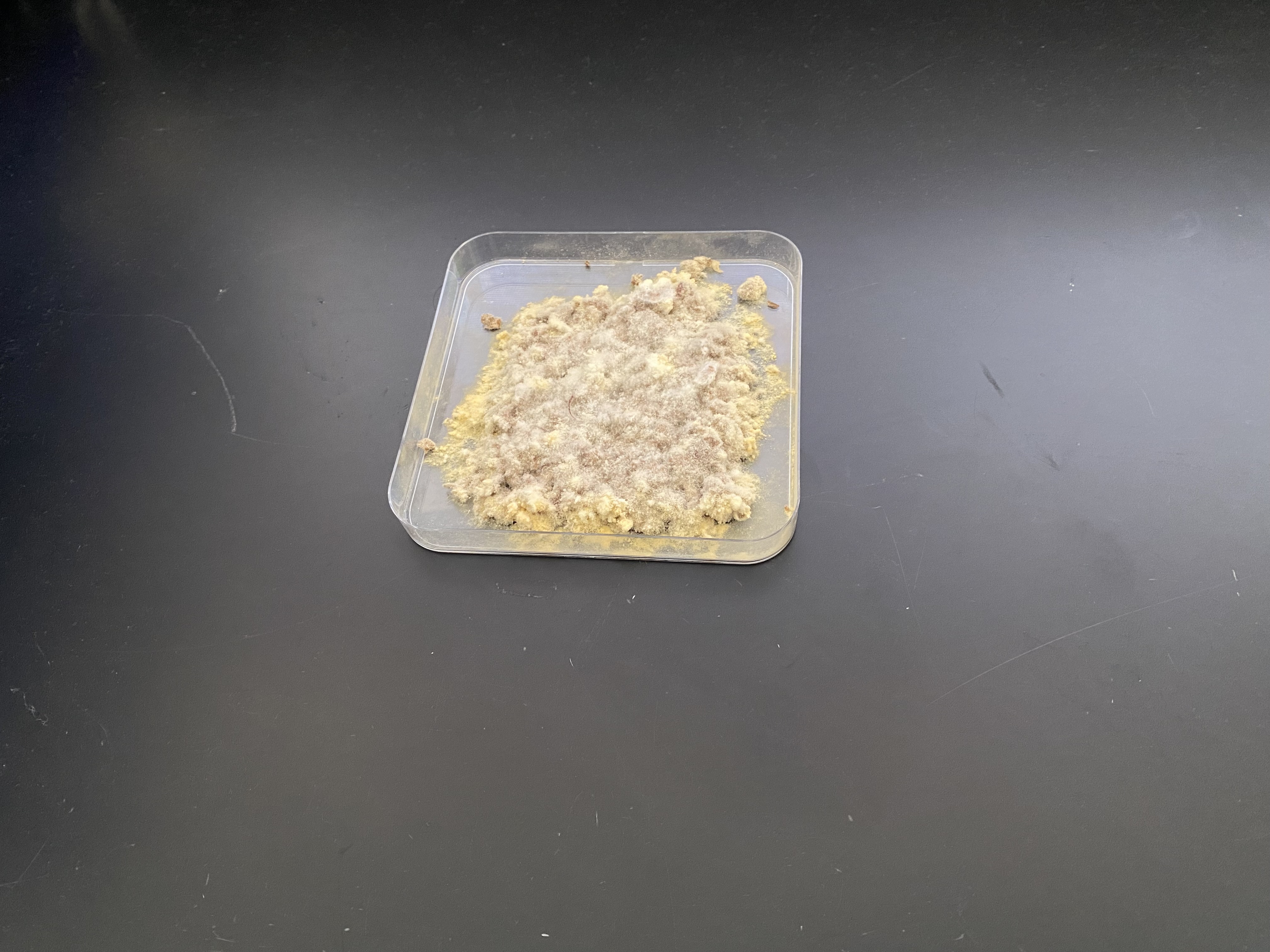

Supplement: Supplementary file 7 — Source data. [file 41564_2024_1799_MOESM7_ESM.zip › Fig4-sourcedata/8_applepomace-NI copy.jpg]

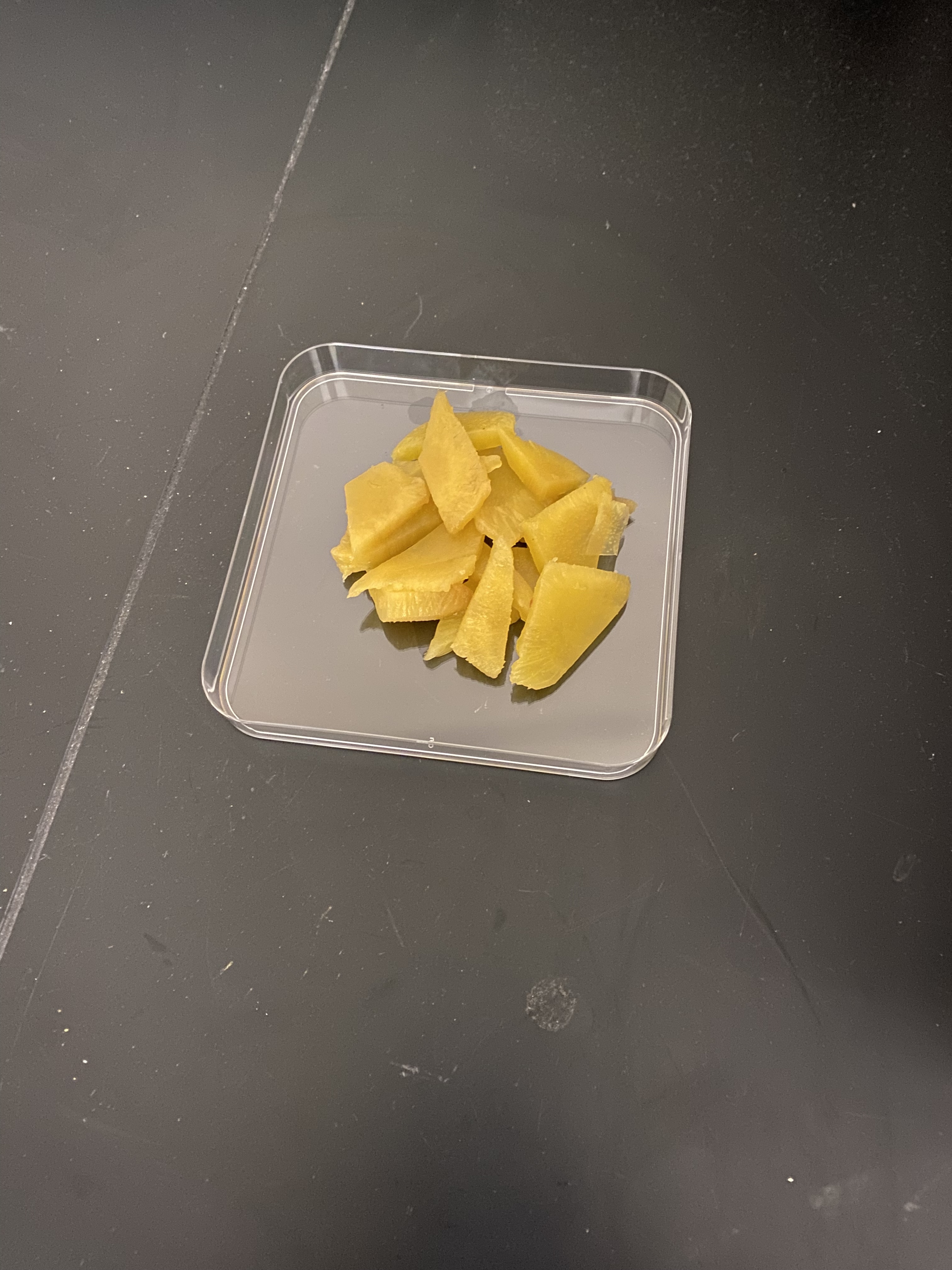

Supplement: Supplementary file 7 — Source data. [file 41564_2024_1799_MOESM7_ESM.zip › Fig4-sourcedata/2023-11-06_pineapplecore copy.jpg]

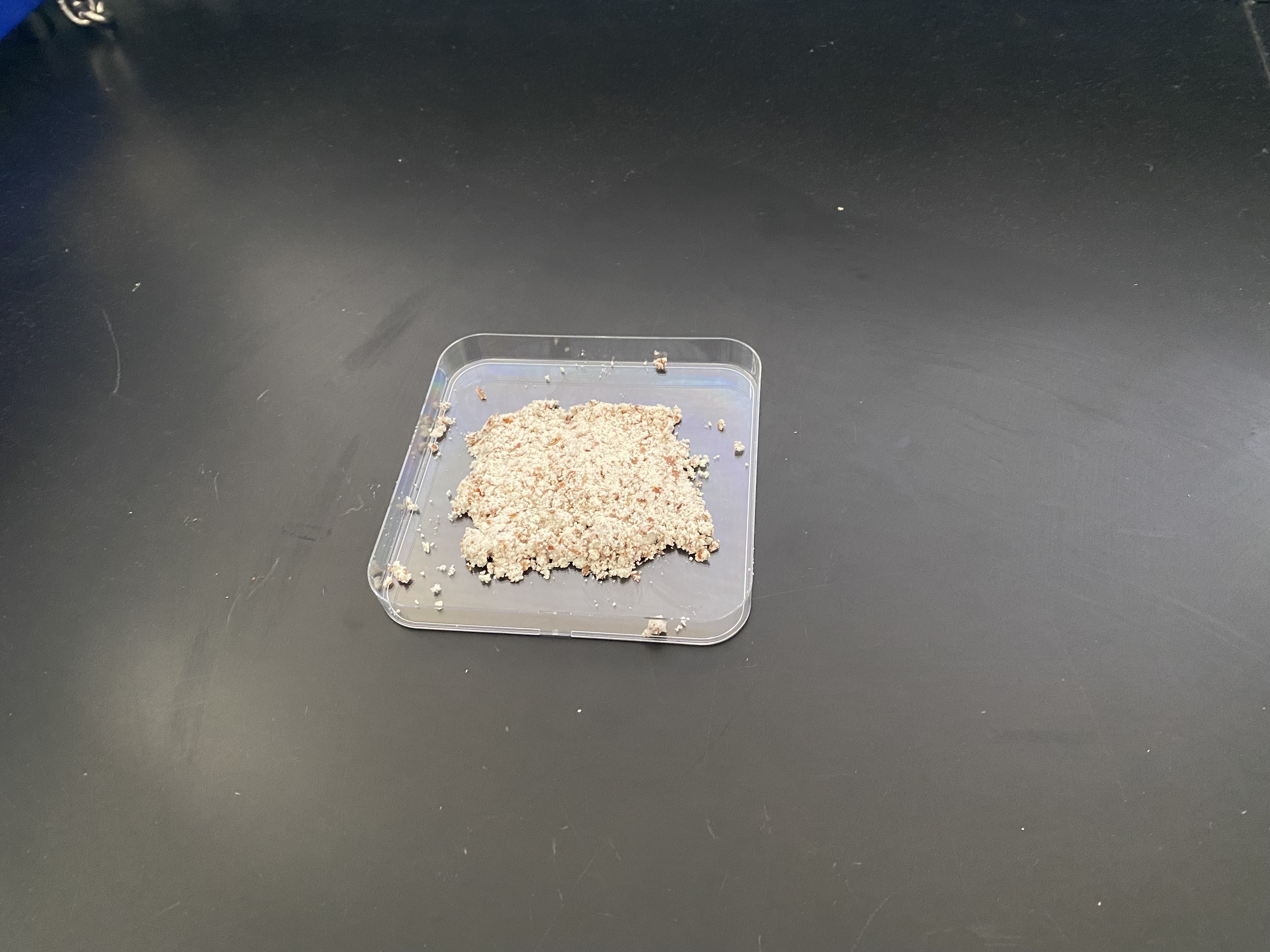

Supplement: Supplementary file 7 — Source data. [file 41564_2024_1799_MOESM7_ESM.zip › Fig4-sourcedata/2023-10-23_almondmilkwaste copy.jpg]

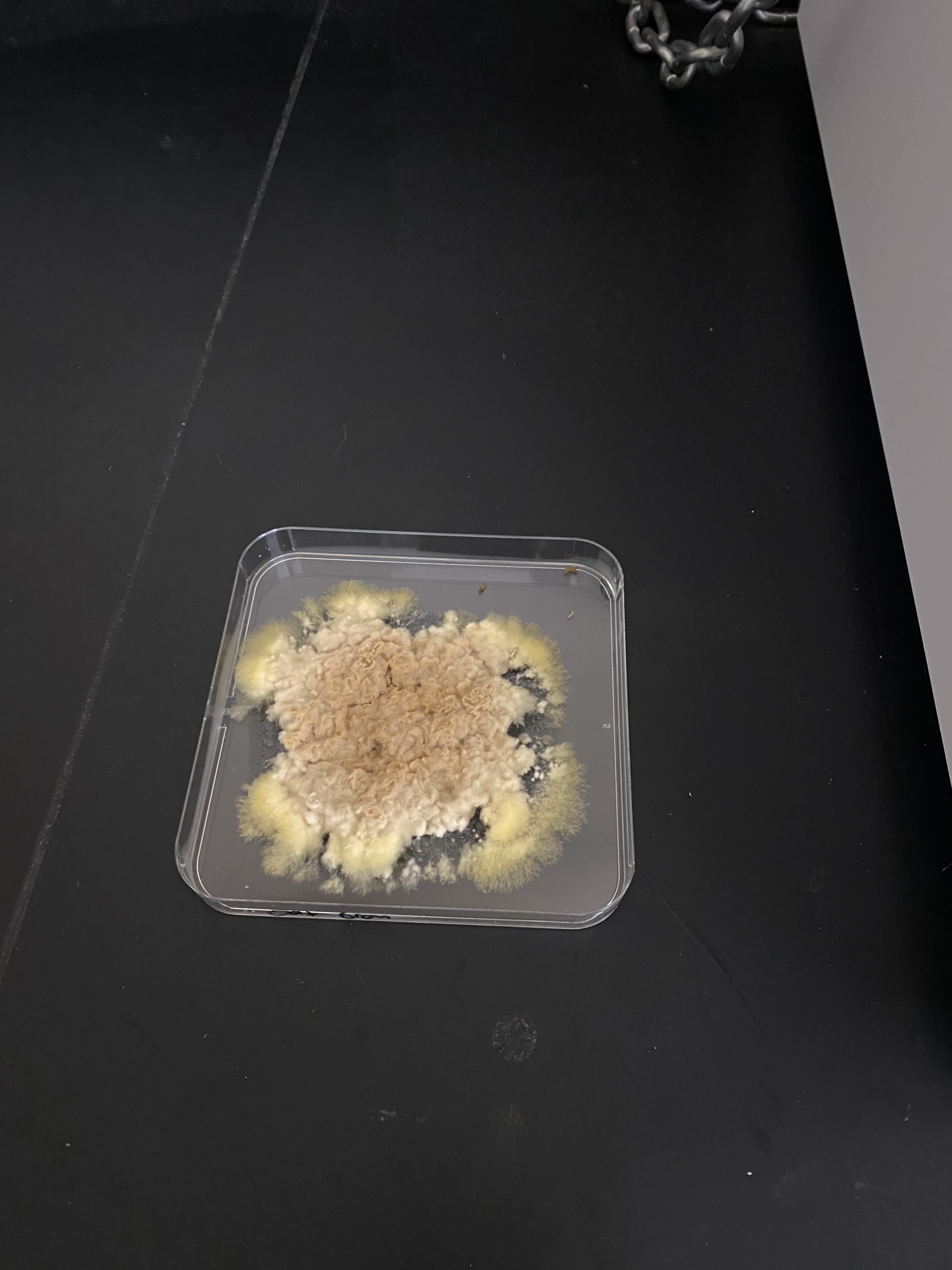

Supplement: Supplementary file 7 — Source data. [file 41564_2024_1799_MOESM7_ESM.zip › Fig4-sourcedata/2023-11-06_oatbran-NI copy.jpg]

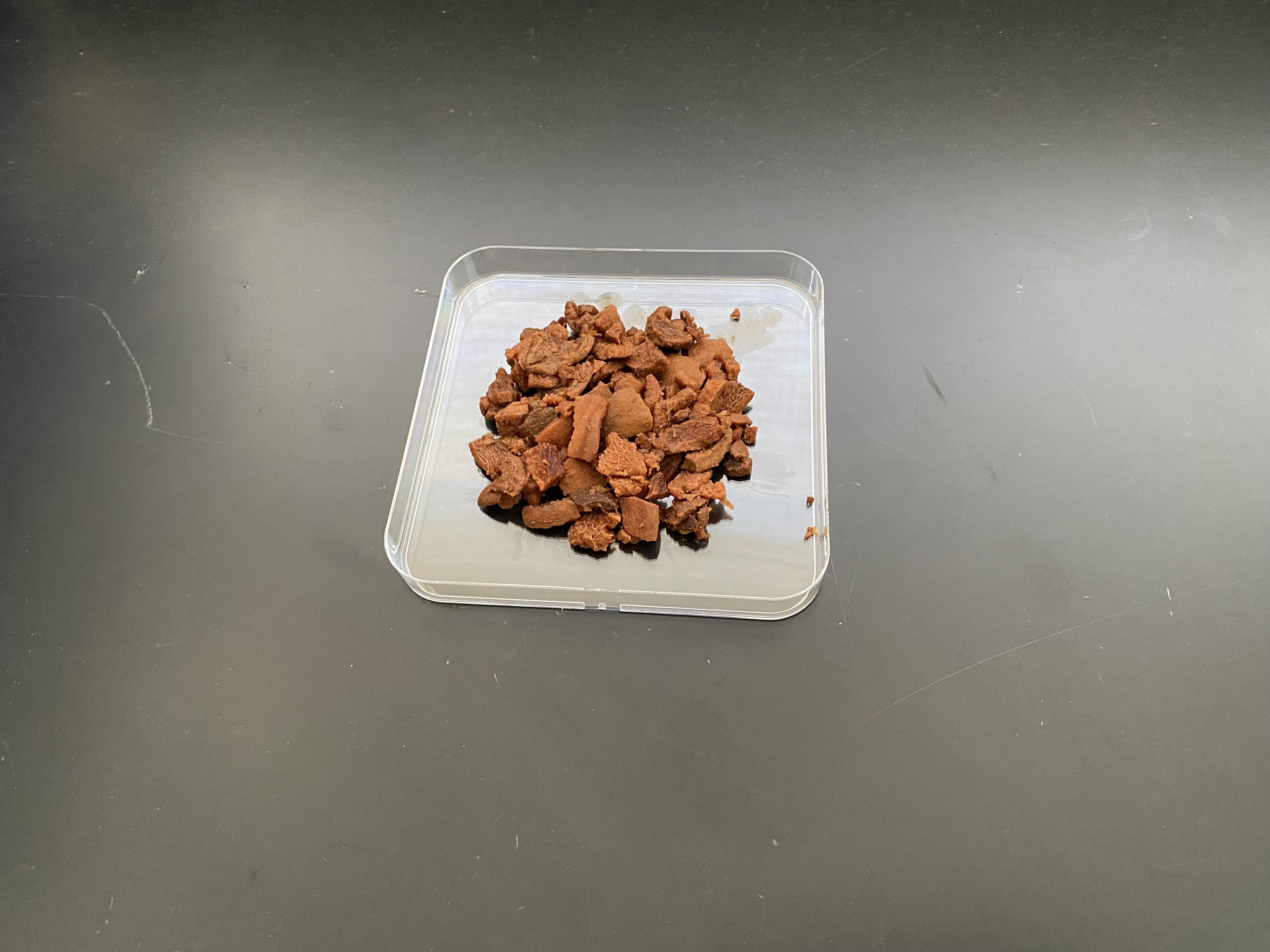

Supplement: Supplementary file 7 — Source data. [file 41564_2024_1799_MOESM7_ESM.zip › Fig4-sourcedata/9_almondhulls copy.jpg]

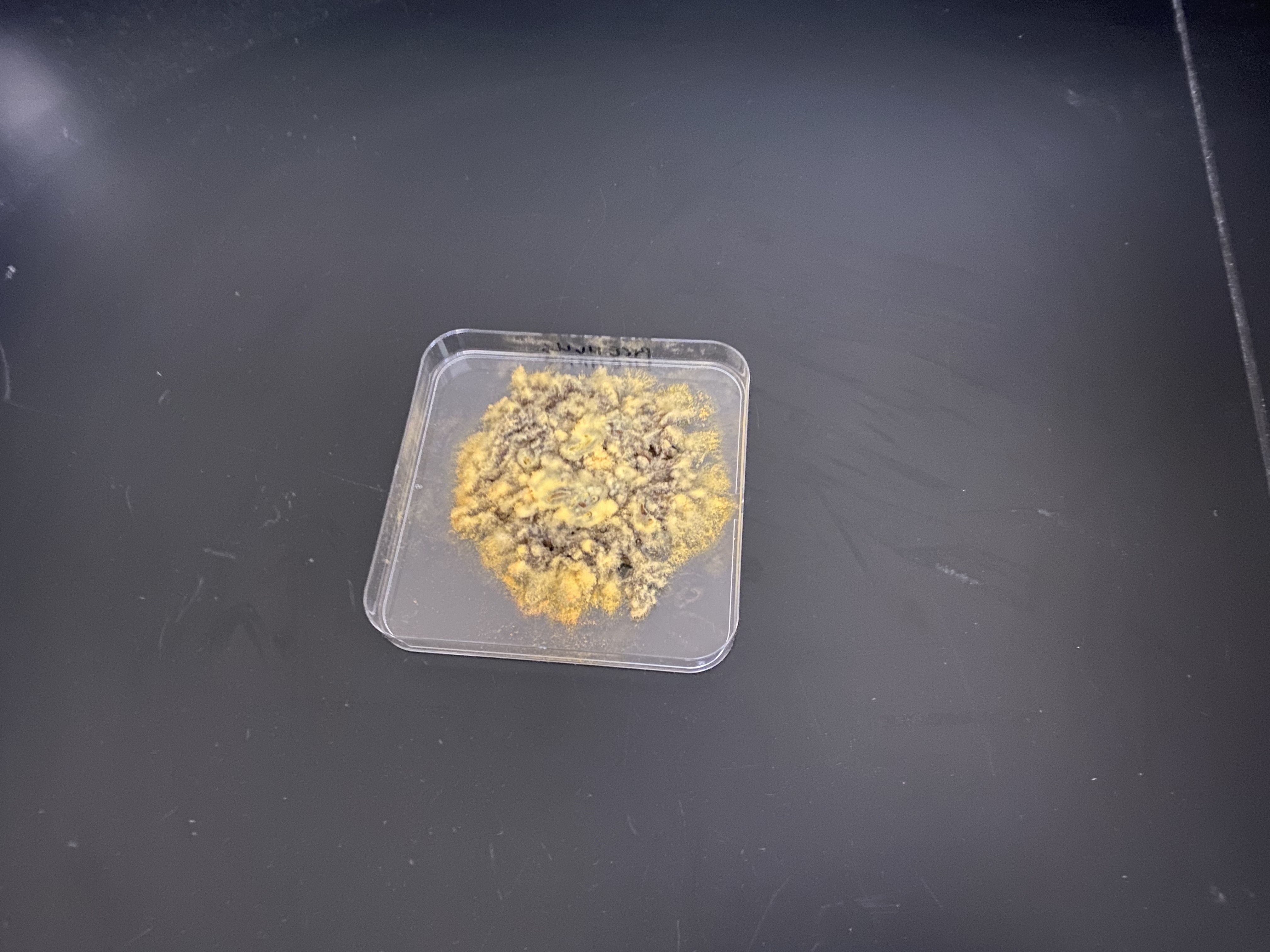

Supplement: Supplementary file 7 — Source data. [file 41564_2024_1799_MOESM7_ESM.zip › Fig4-sourcedata/2023-11-06_ricehulls-NI copy.jpg]

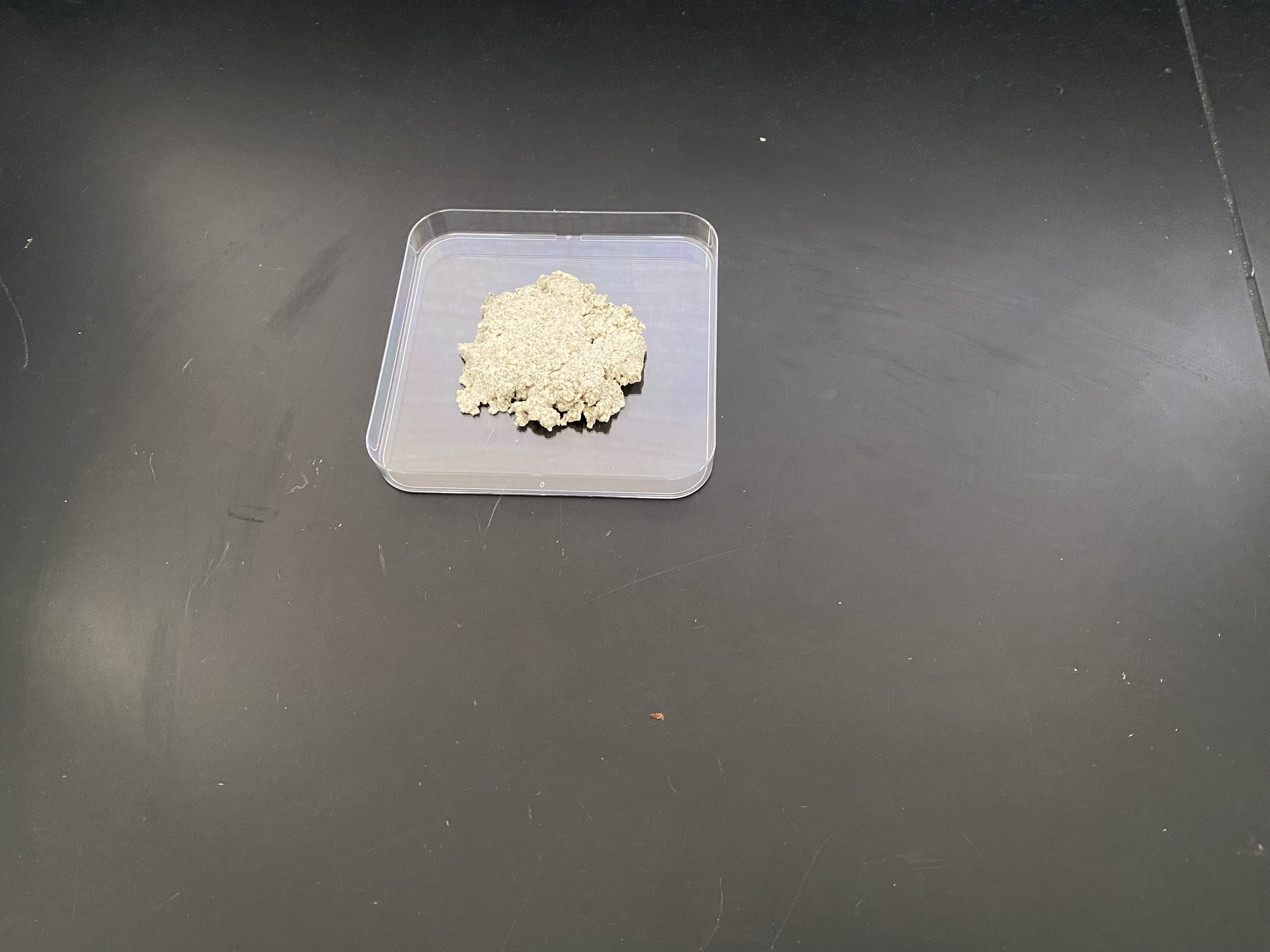

Supplement: Supplementary file 7 — Source data. [file 41564_2024_1799_MOESM7_ESM.zip › Fig4-sourcedata/2023-10-23_hempmilkwaste copy.jpg]

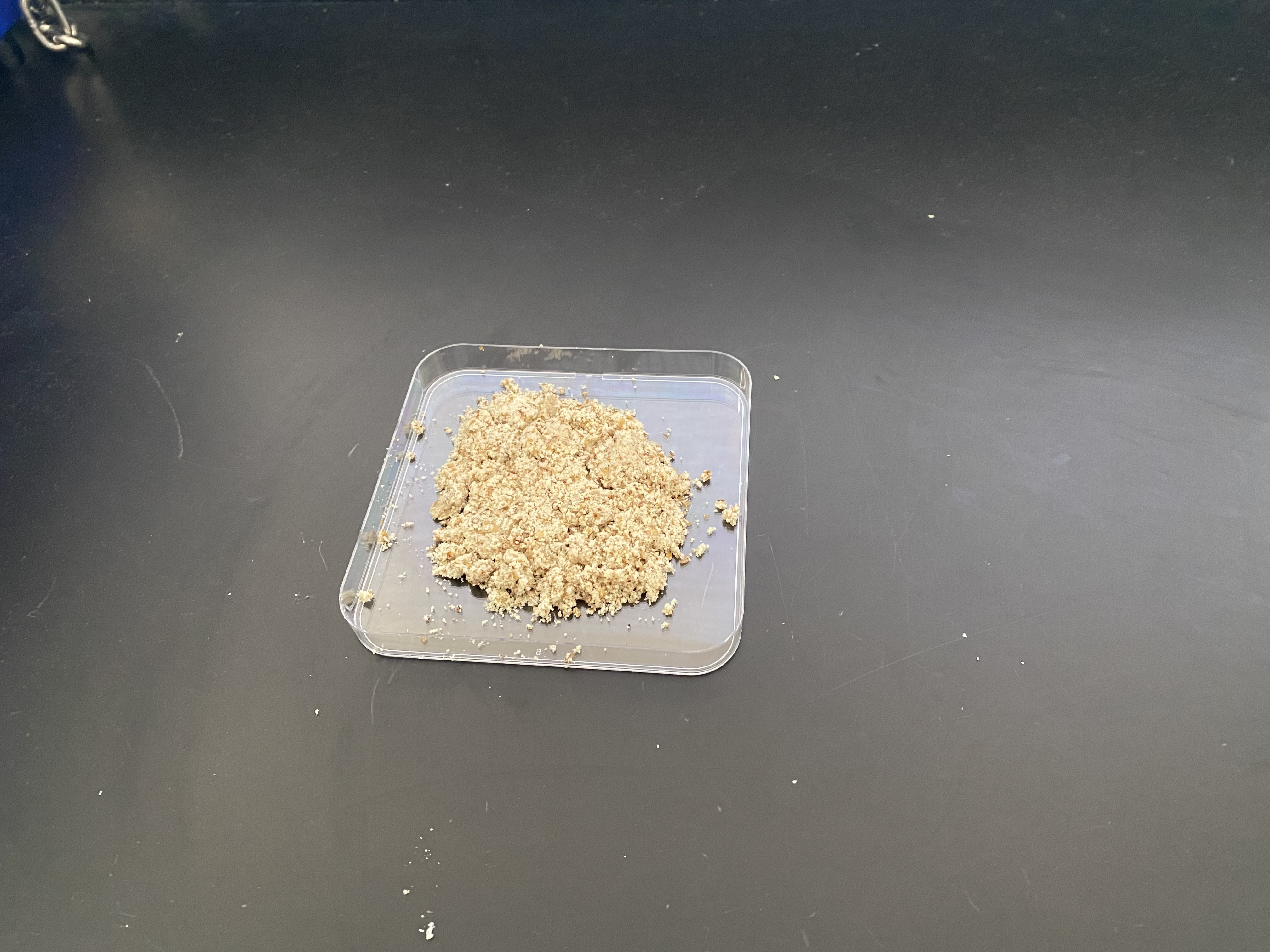

Supplement: Supplementary file 7 — Source data. [file 41564_2024_1799_MOESM7_ESM.zip › Fig4-sourcedata/2023-10-23_tigernutmilkwaste copy.jpg]

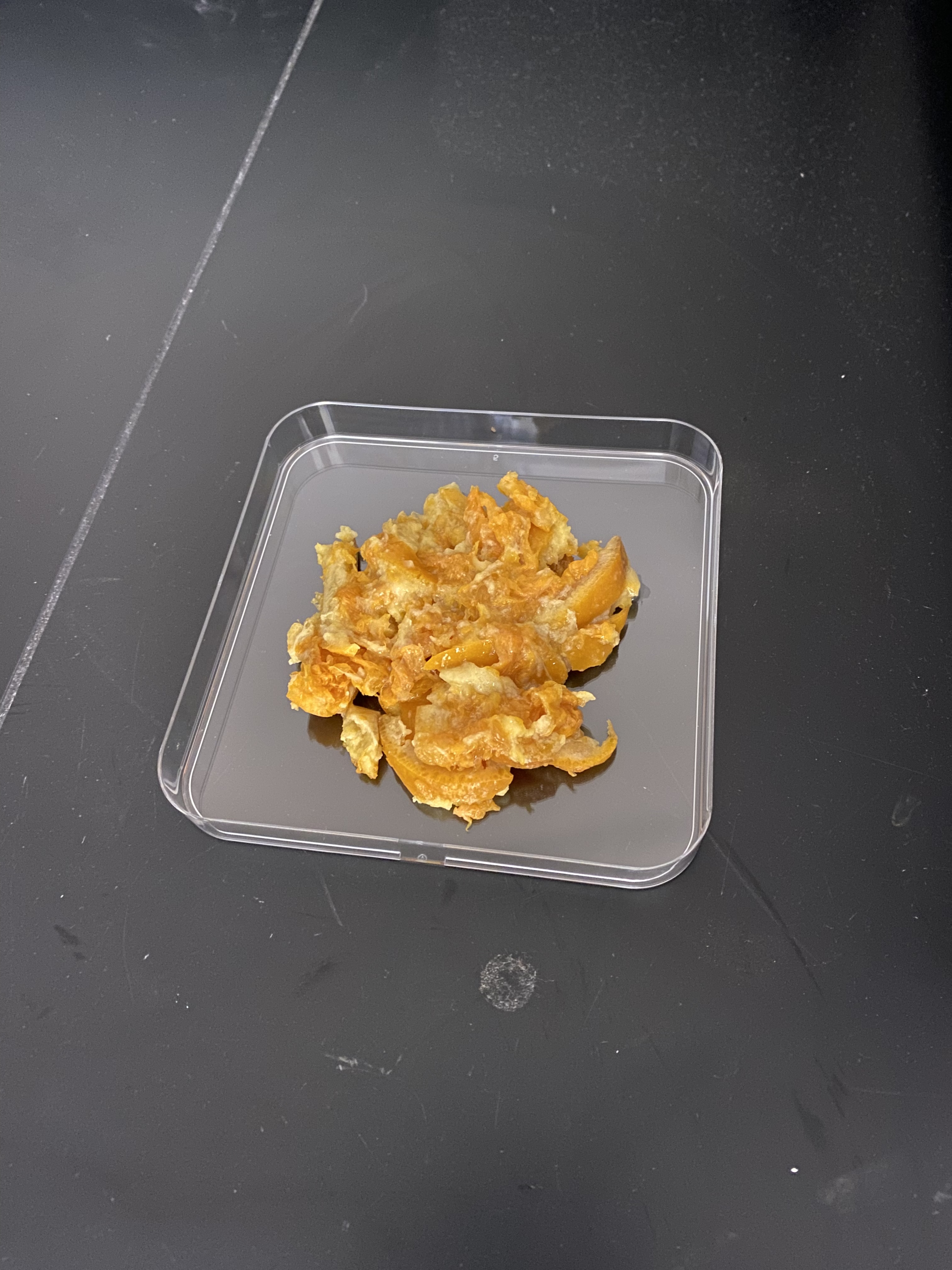

Supplement: Supplementary file 7 — Source data. [file 41564_2024_1799_MOESM7_ESM.zip › Fig4-sourcedata/2023-11-06_orangepeels copy.jpg]

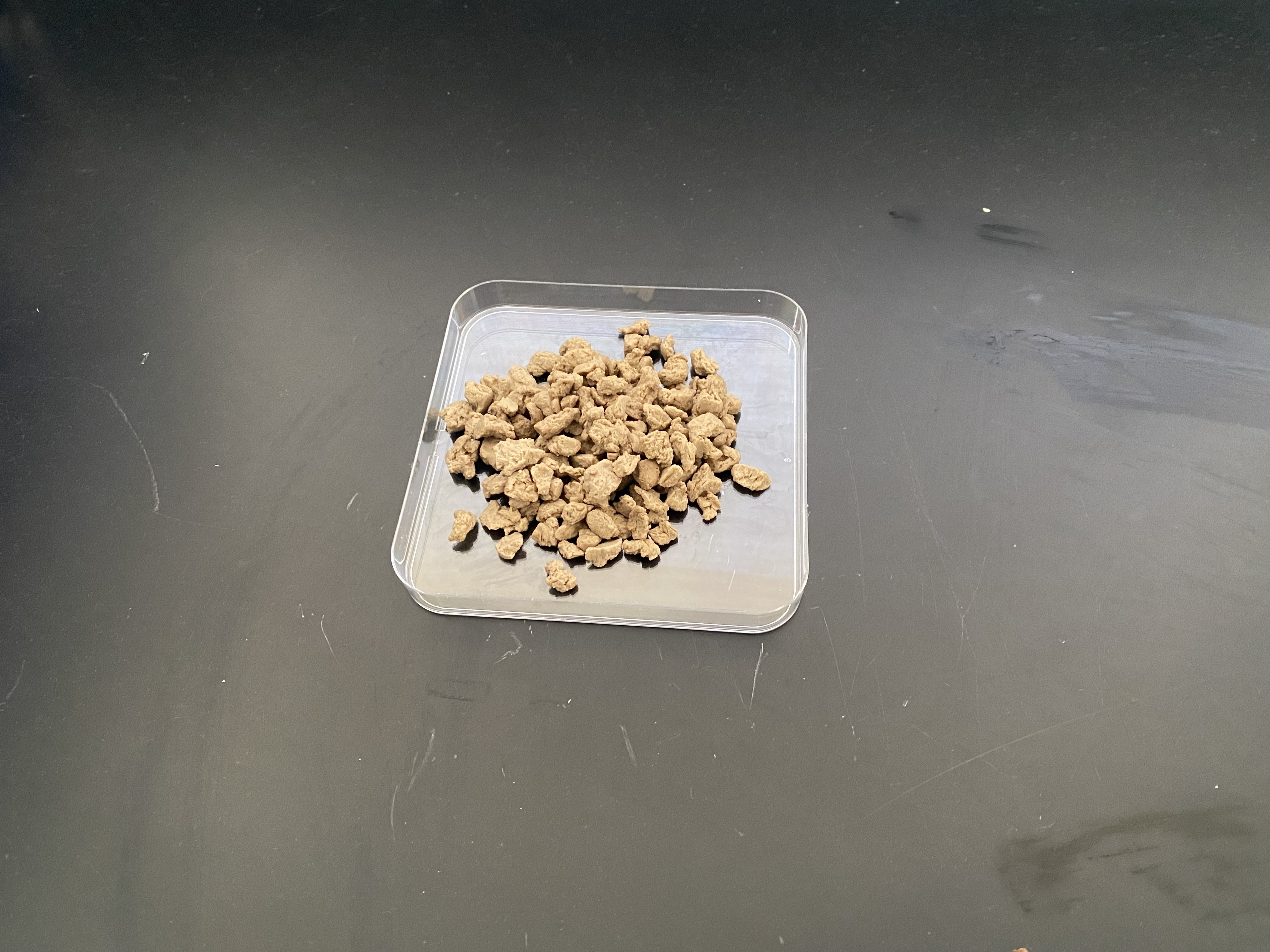

Supplement: Supplementary file 7 — Source data. [file 41564_2024_1799_MOESM7_ESM.zip › Fig4-sourcedata/2023-10-23_sunflowerseedTVP copy.jpg]

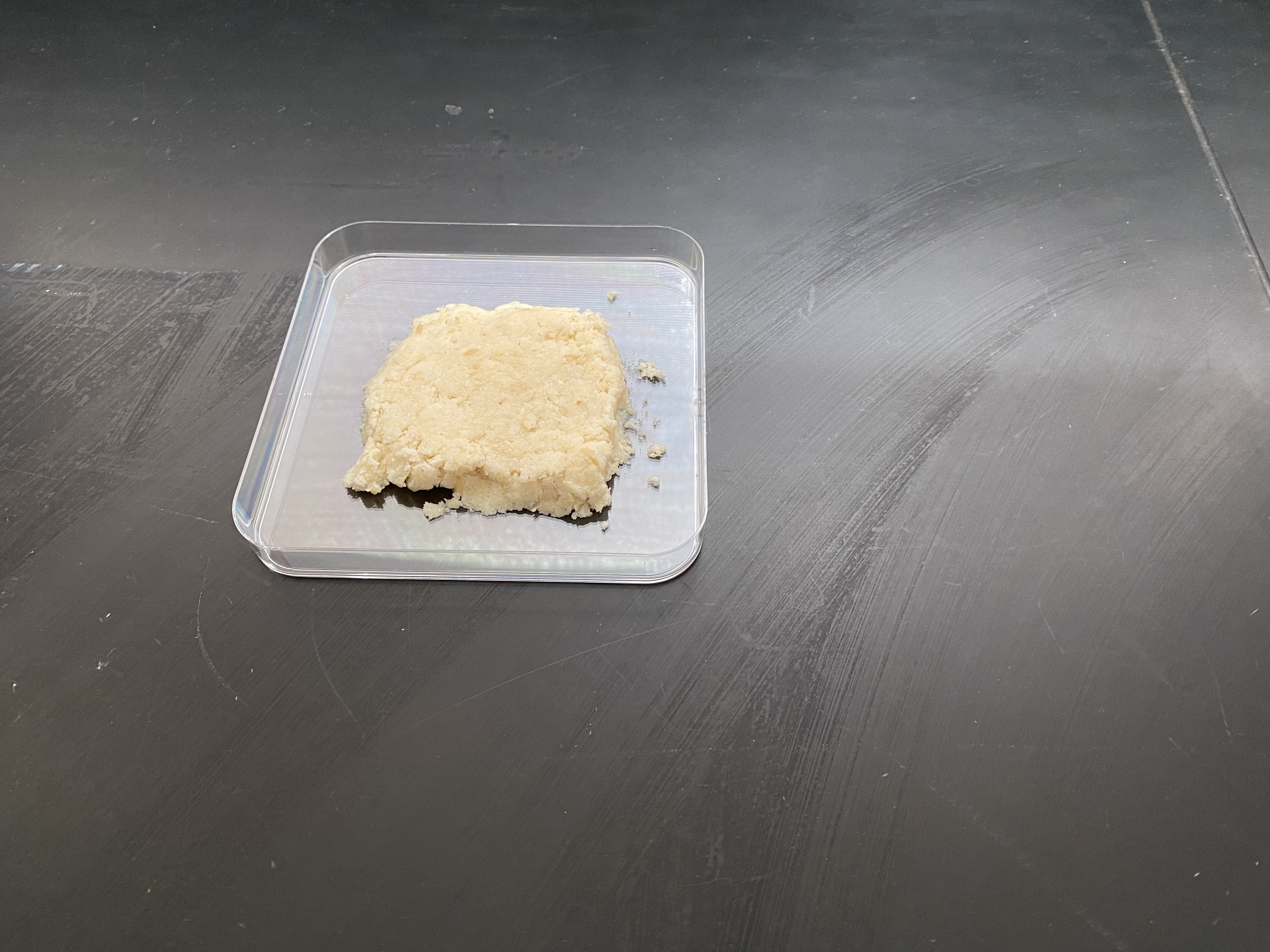

Supplement: Supplementary file 7 — Source data. [file 41564_2024_1799_MOESM7_ESM.zip › Fig4-sourcedata/Okara copy.jpg]

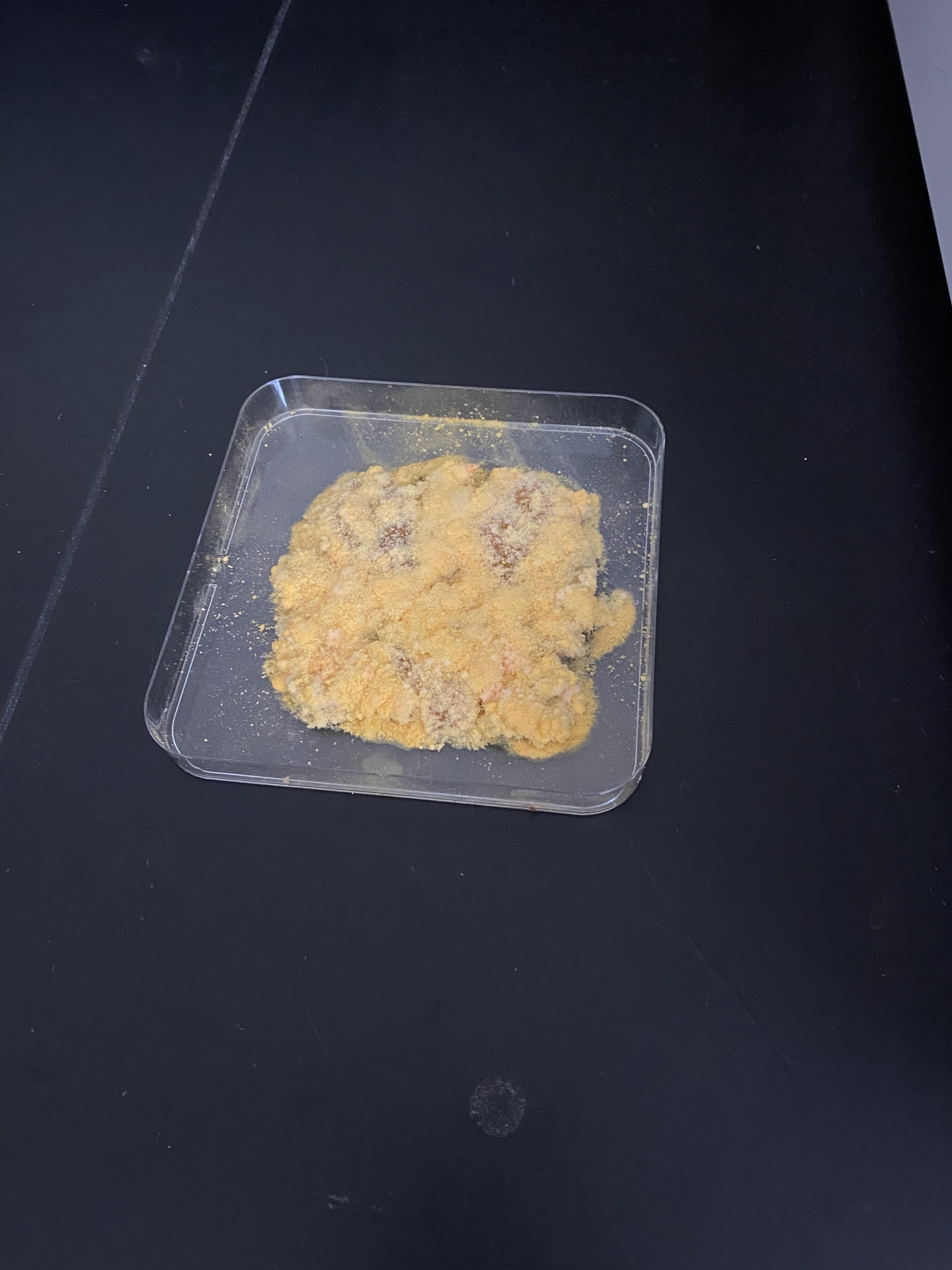

Supplement: Supplementary file 7 — Source data. [file 41564_2024_1799_MOESM7_ESM.zip › Fig4-sourcedata/2023-11-06_orangepeels-NI copy.jpg]

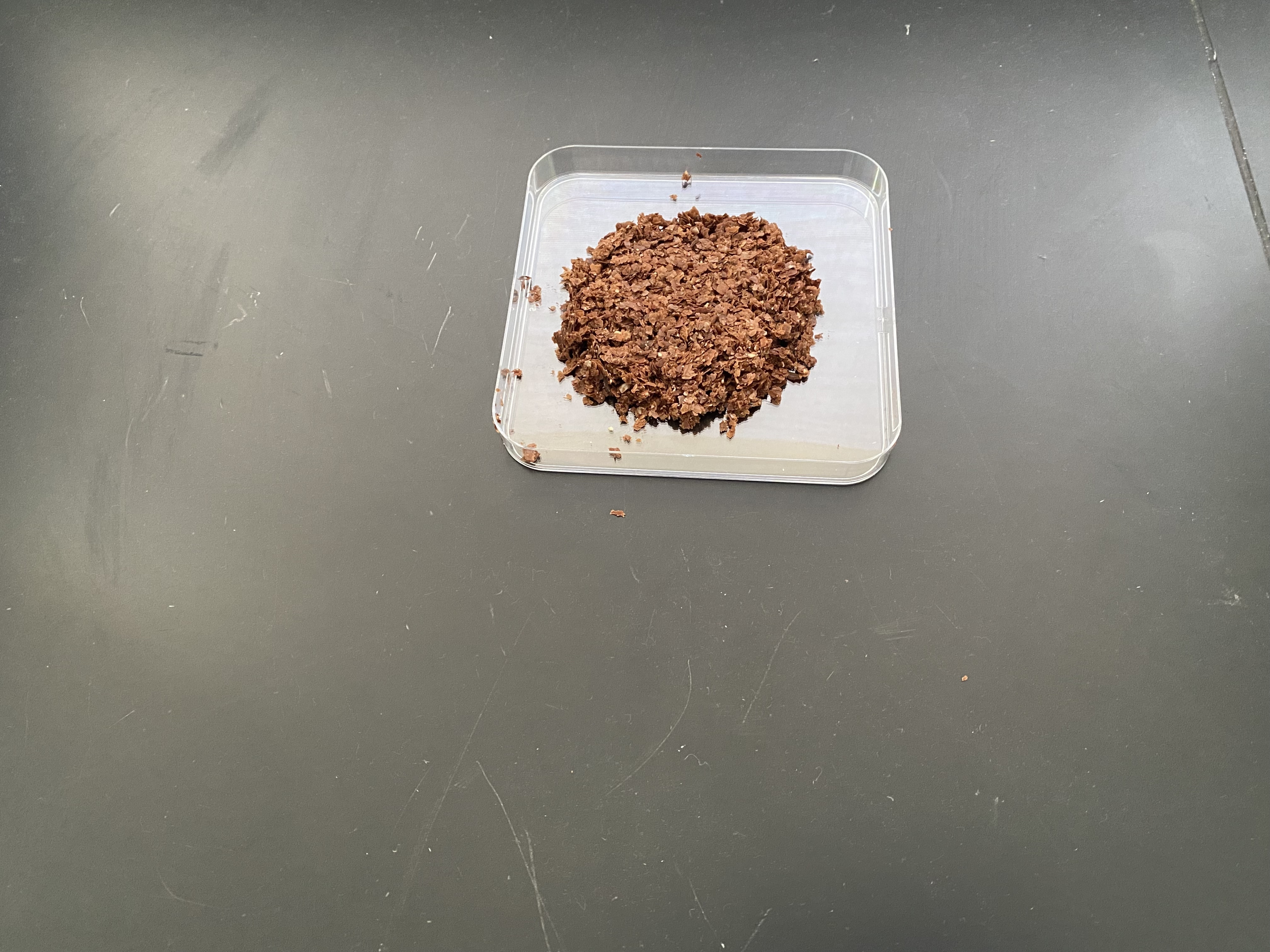

Supplement: Supplementary file 7 — Source data. [file 41564_2024_1799_MOESM7_ESM.zip › Fig4-sourcedata/2023-10-23_almondskins copy.jpg]

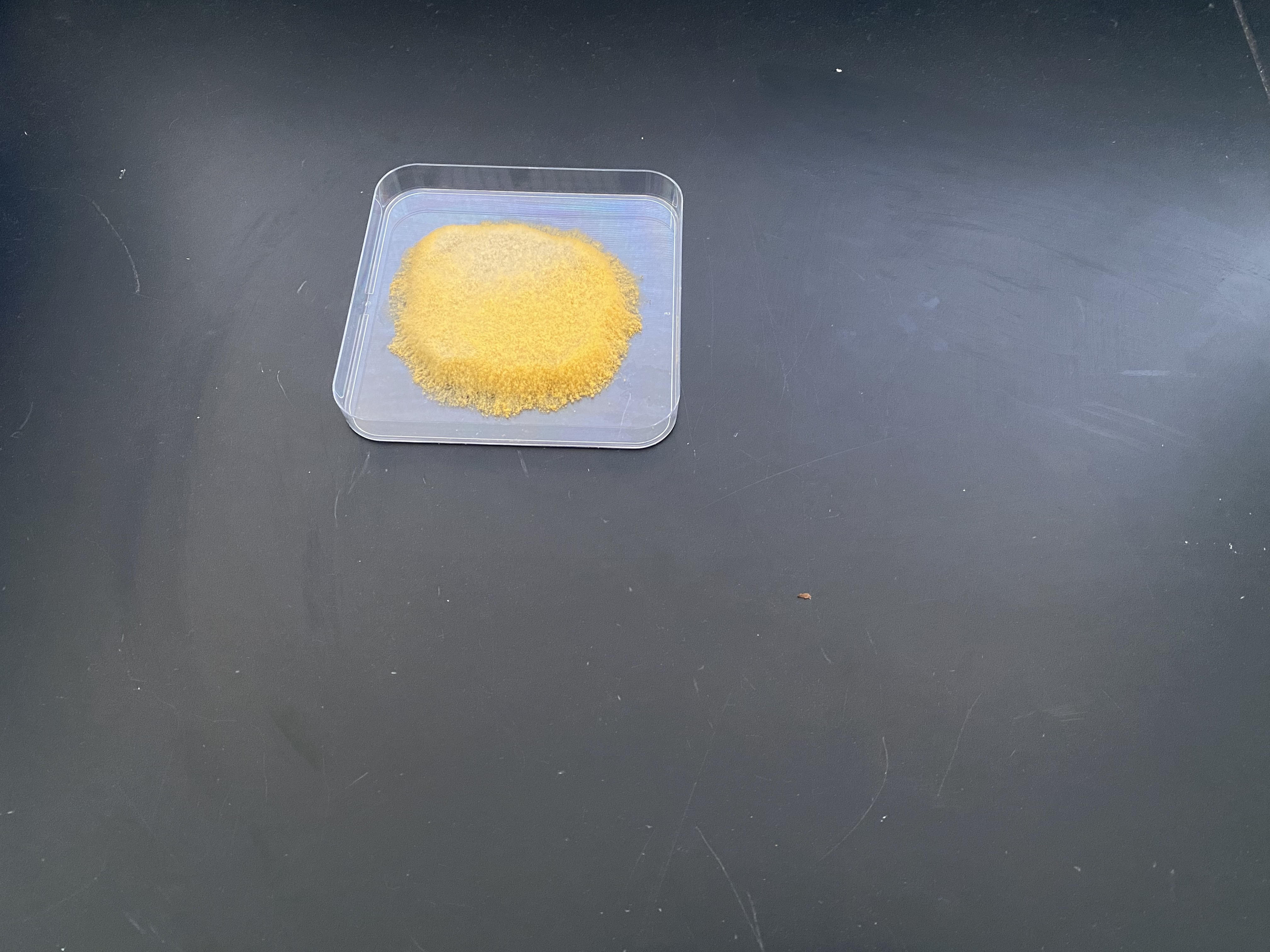

Supplement: Supplementary file 7 — Source data. [file 41564_2024_1799_MOESM7_ESM.zip › Fig4-sourcedata/2023-10-23_hempmilkwaste-NI copy.jpg]

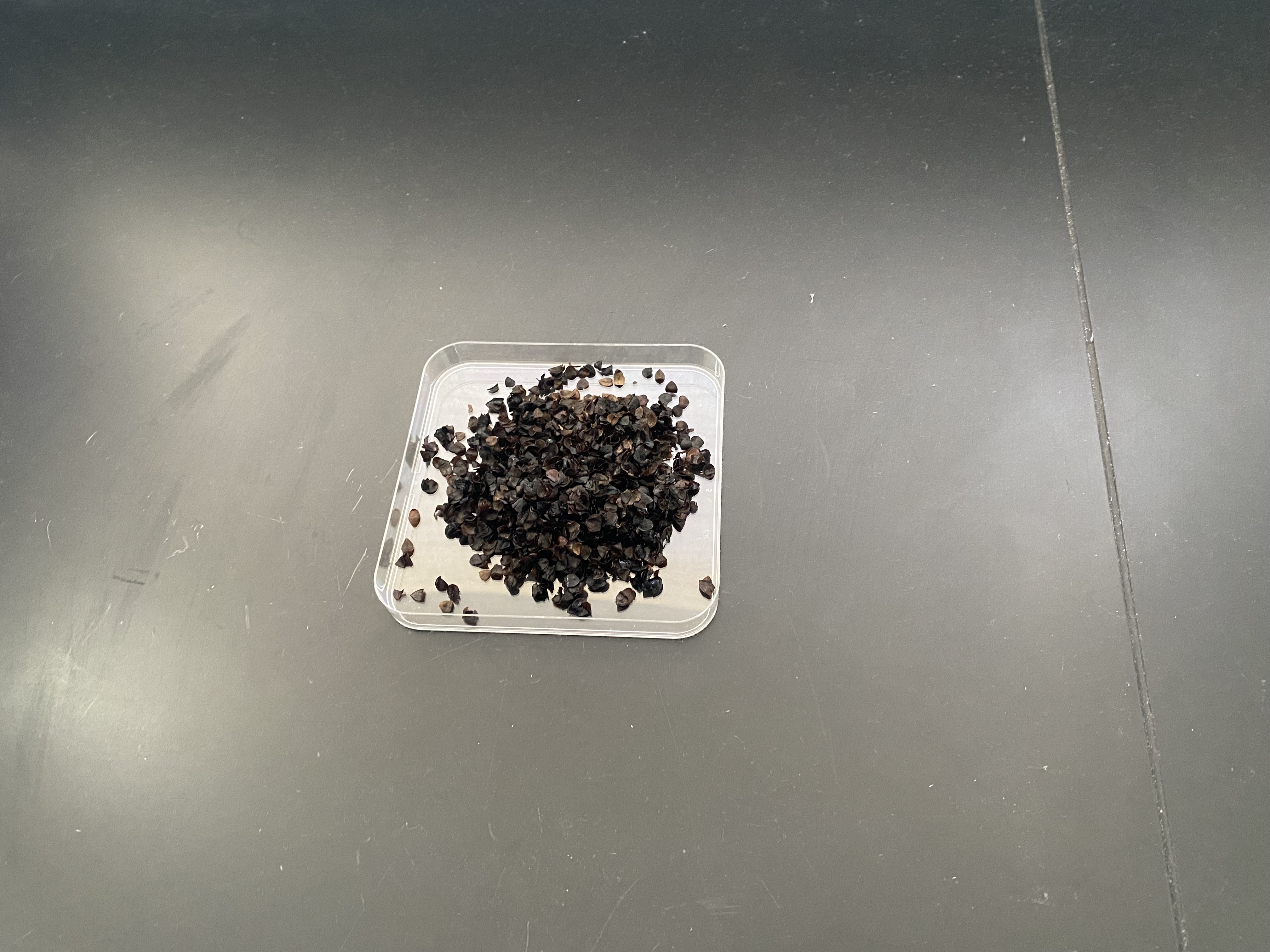

Supplement: Supplementary file 7 — Source data. [file 41564_2024_1799_MOESM7_ESM.zip › Fig4-sourcedata/2023-10-23_buckwheathulls copy.jpg]

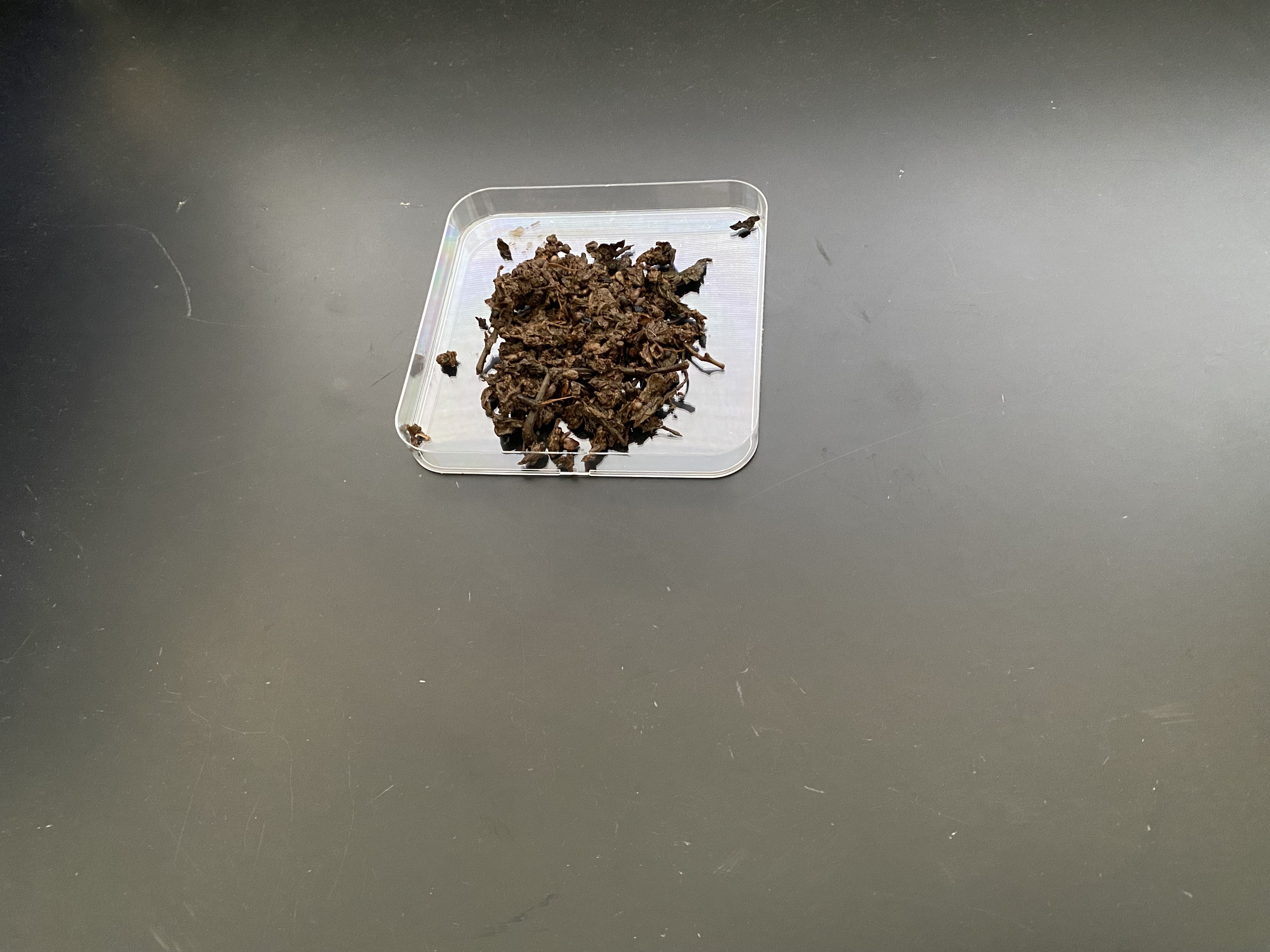

Supplement: Supplementary file 7 — Source data. [file 41564_2024_1799_MOESM7_ESM.zip › Fig4-sourcedata/10_grapepomace copy.jpg]

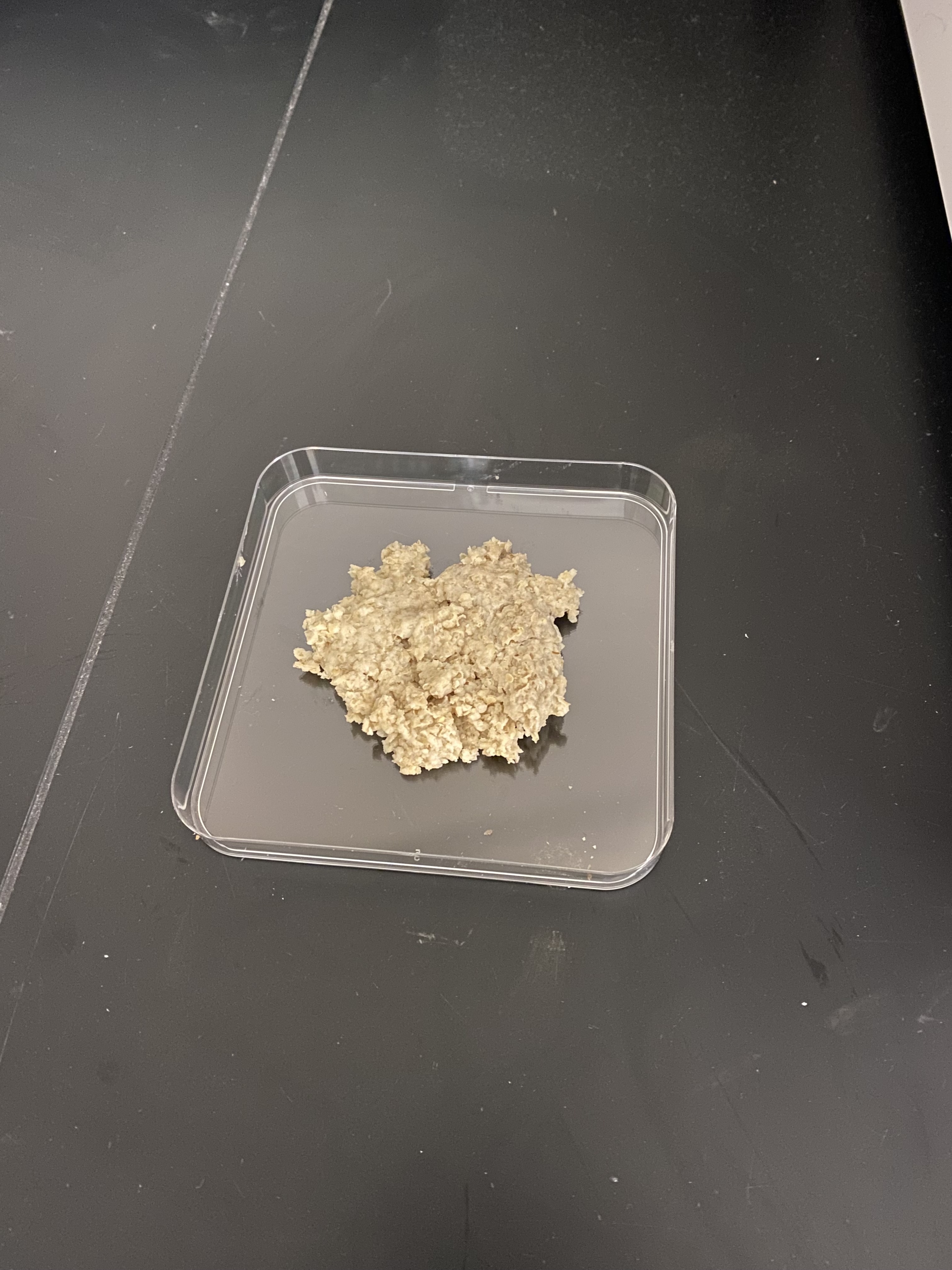

Supplement: Supplementary file 7 — Source data. [file 41564_2024_1799_MOESM7_ESM.zip › Fig4-sourcedata/2023-11-06_oatbran copy.jpg]

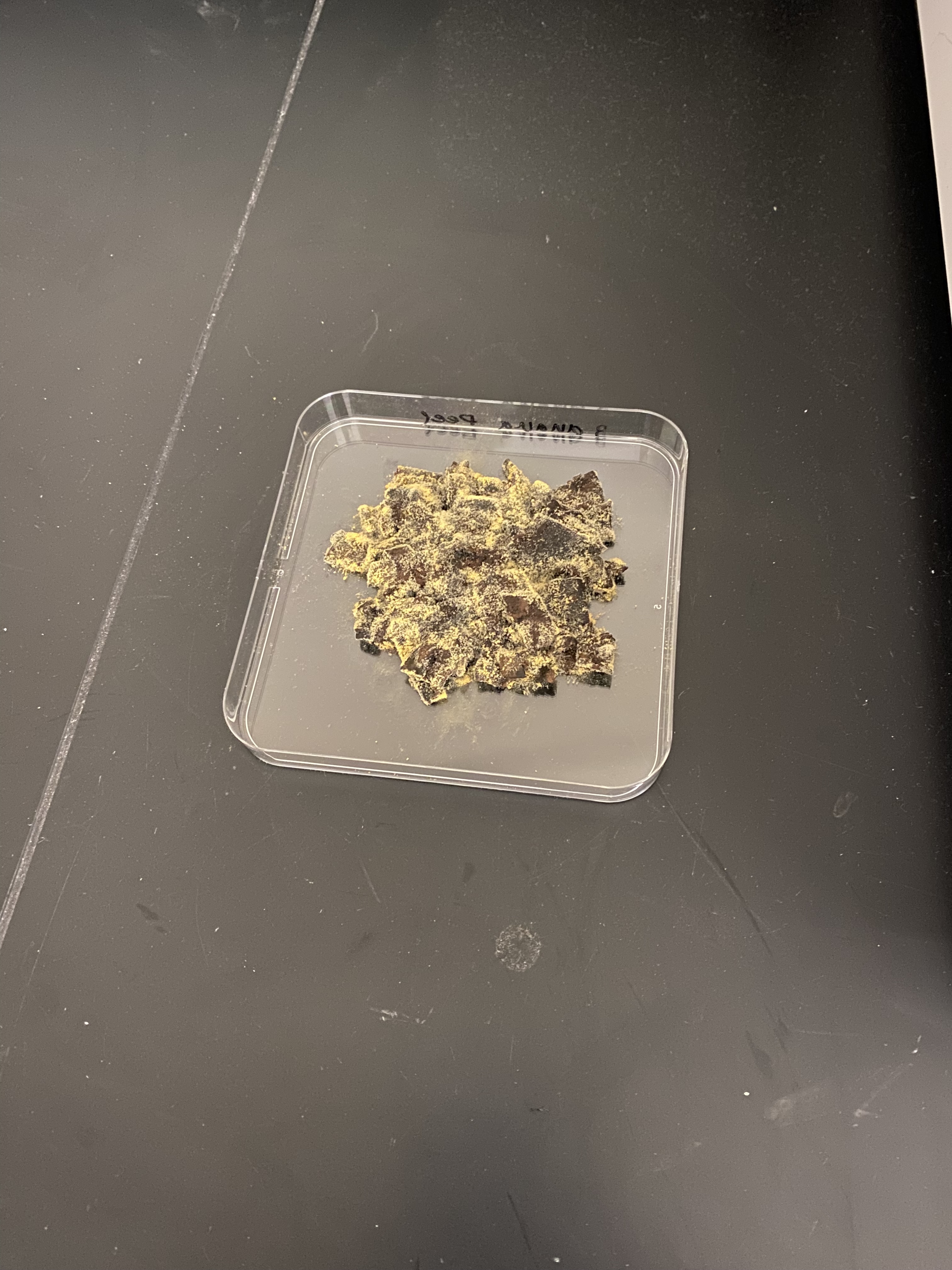

Supplement: Supplementary file 7 — Source data. [file 41564_2024_1799_MOESM7_ESM.zip › Fig4-sourcedata/2023-11-06_bananapeels-NI copy.jpg]

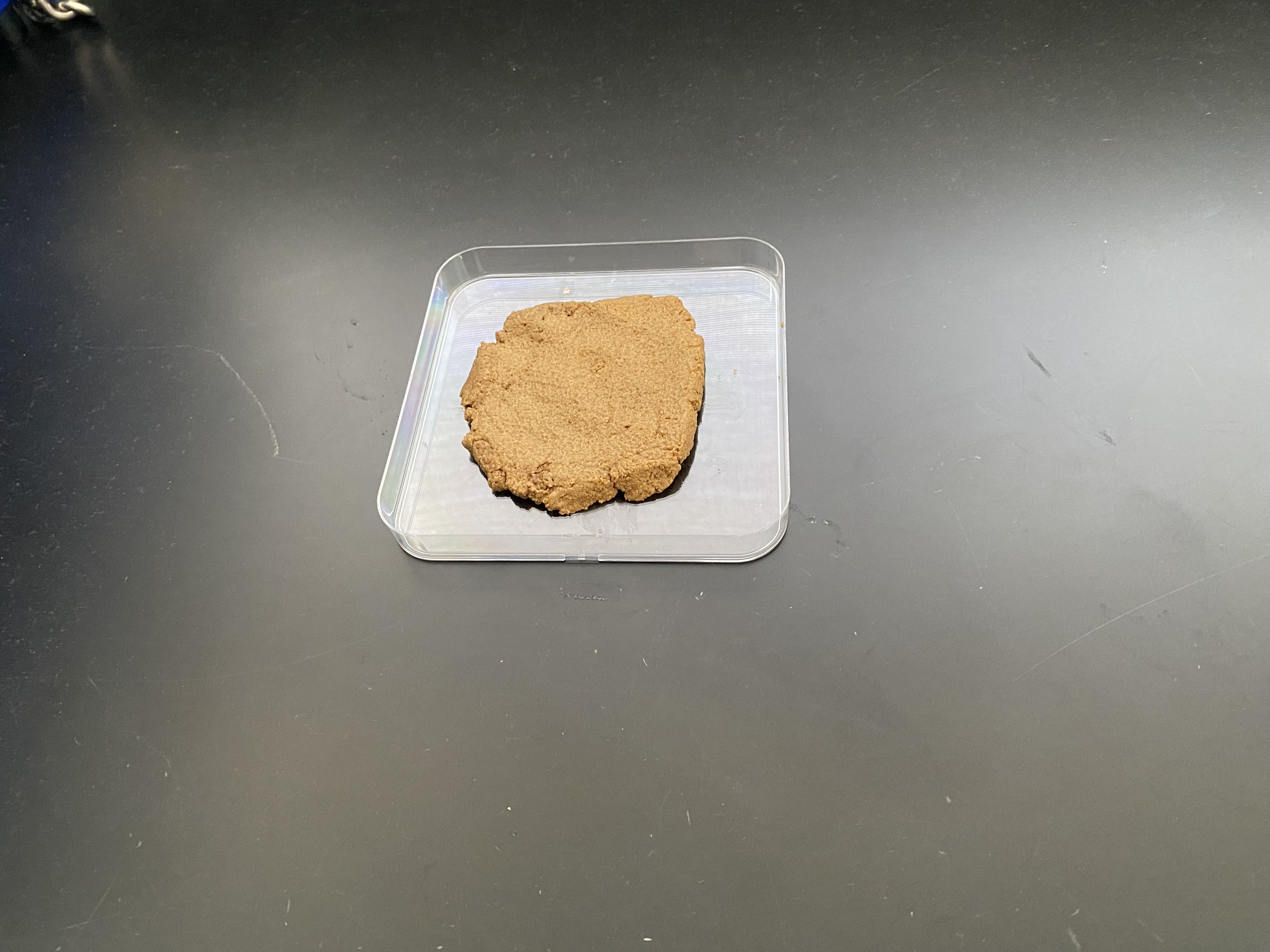

Supplement: Supplementary file 7 — Source data. [file 41564_2024_1799_MOESM7_ESM.zip › Fig4-sourcedata/5_pumpkinseedpresscake copy.jpg]

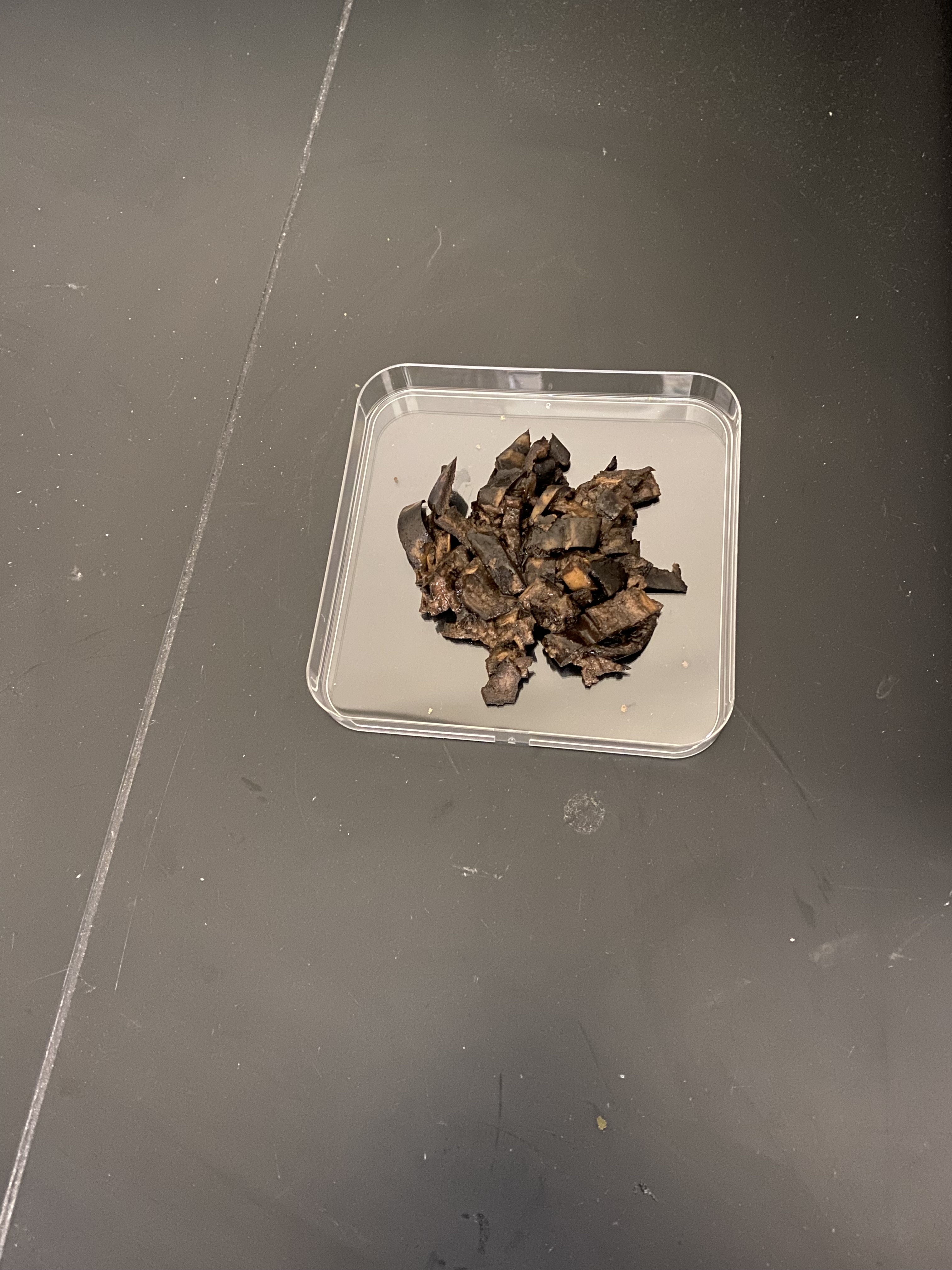

Supplement: Supplementary file 7 — Source data. [file 41564_2024_1799_MOESM7_ESM.zip › Fig4-sourcedata/2023-11-06_bananapeels copy.jpg]

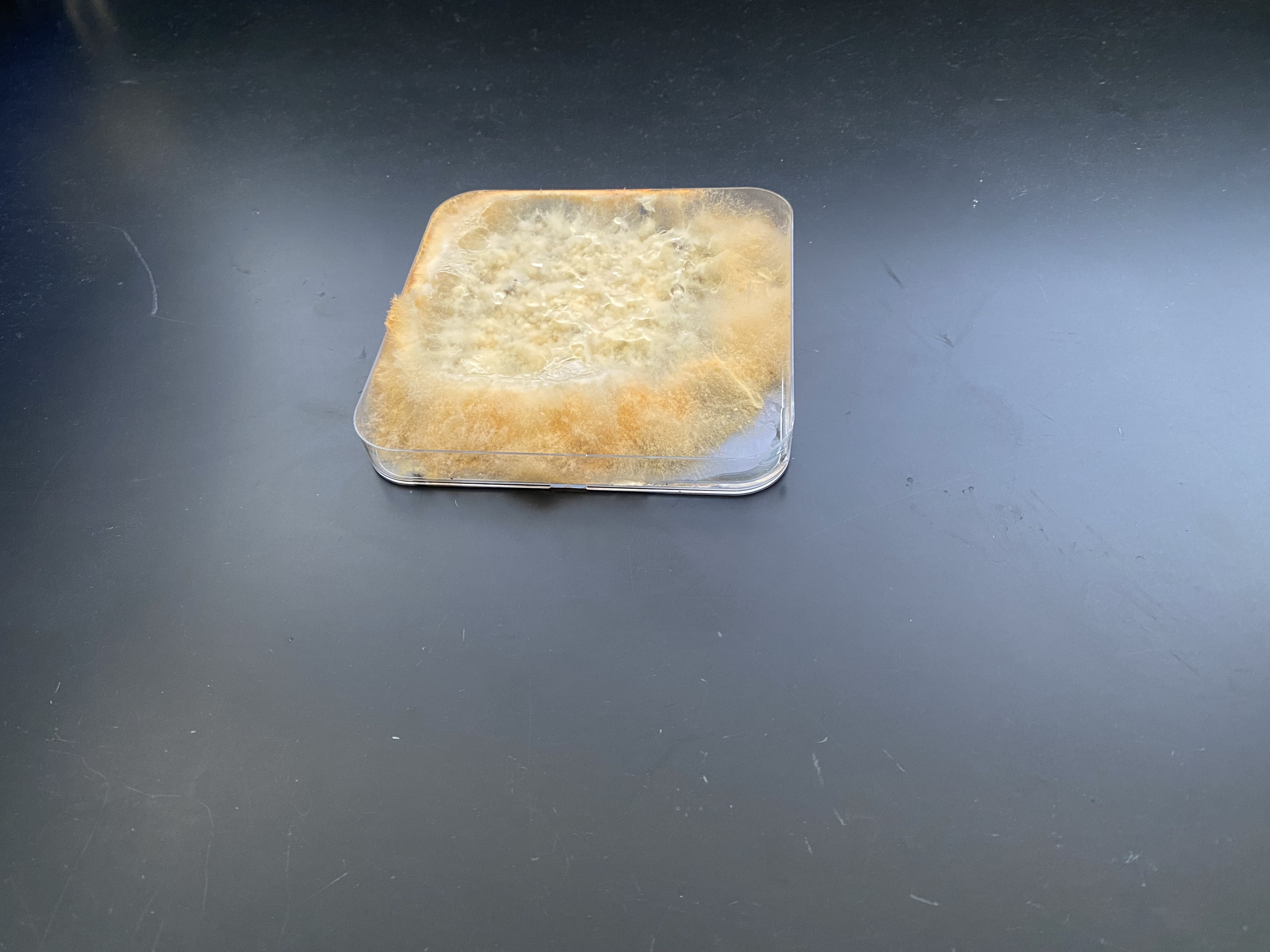

Supplement: Supplementary file 7 — Source data. [file 41564_2024_1799_MOESM7_ESM.zip › Fig4-sourcedata/3_ricemilkwaste-NI copy.jpg]

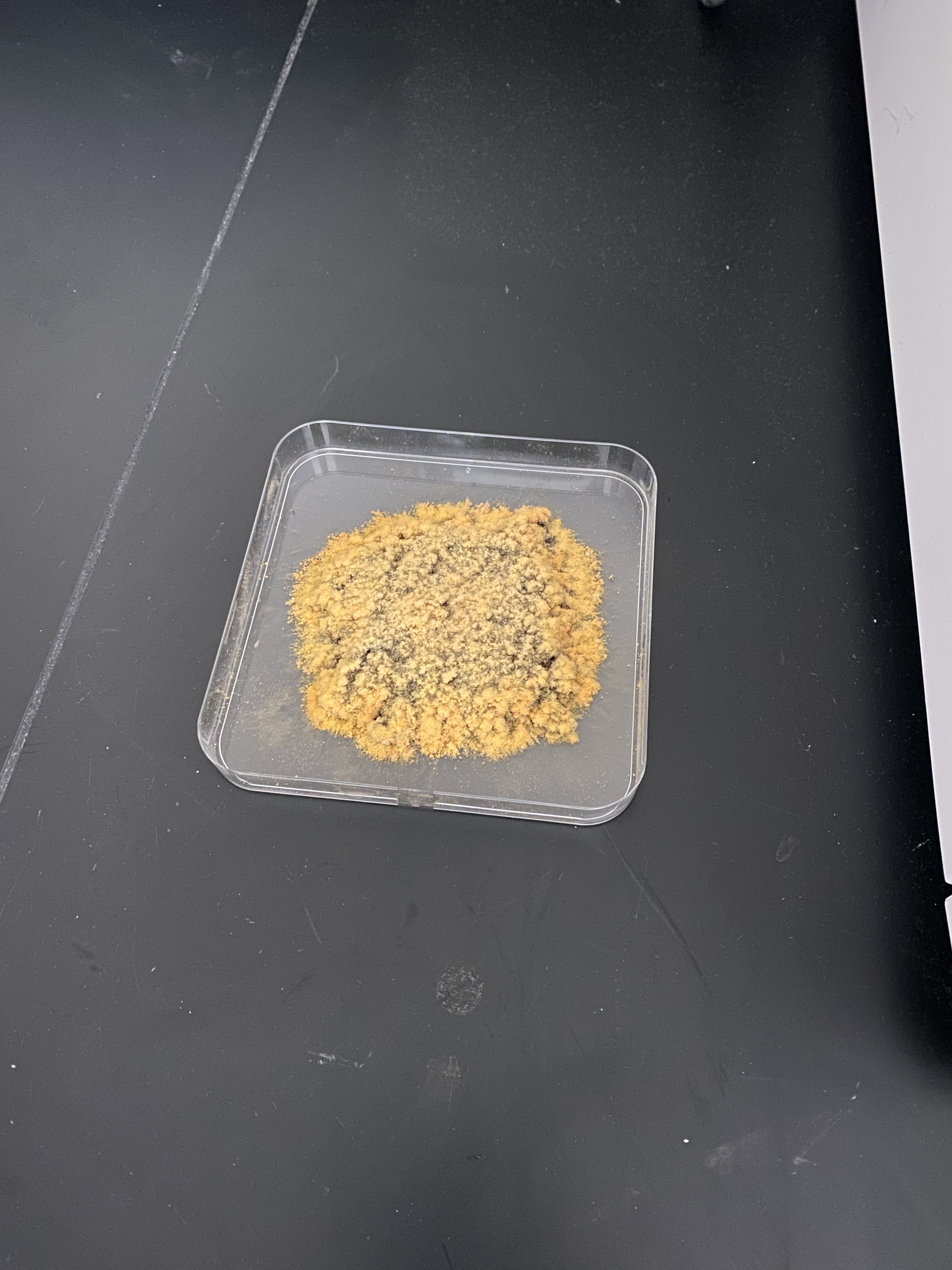

Supplement: Supplementary file 7 — Source data. [file 41564_2024_1799_MOESM7_ESM.zip › Fig4-sourcedata/2023-11-06_oro_barleyhull-husks copy.jpg]

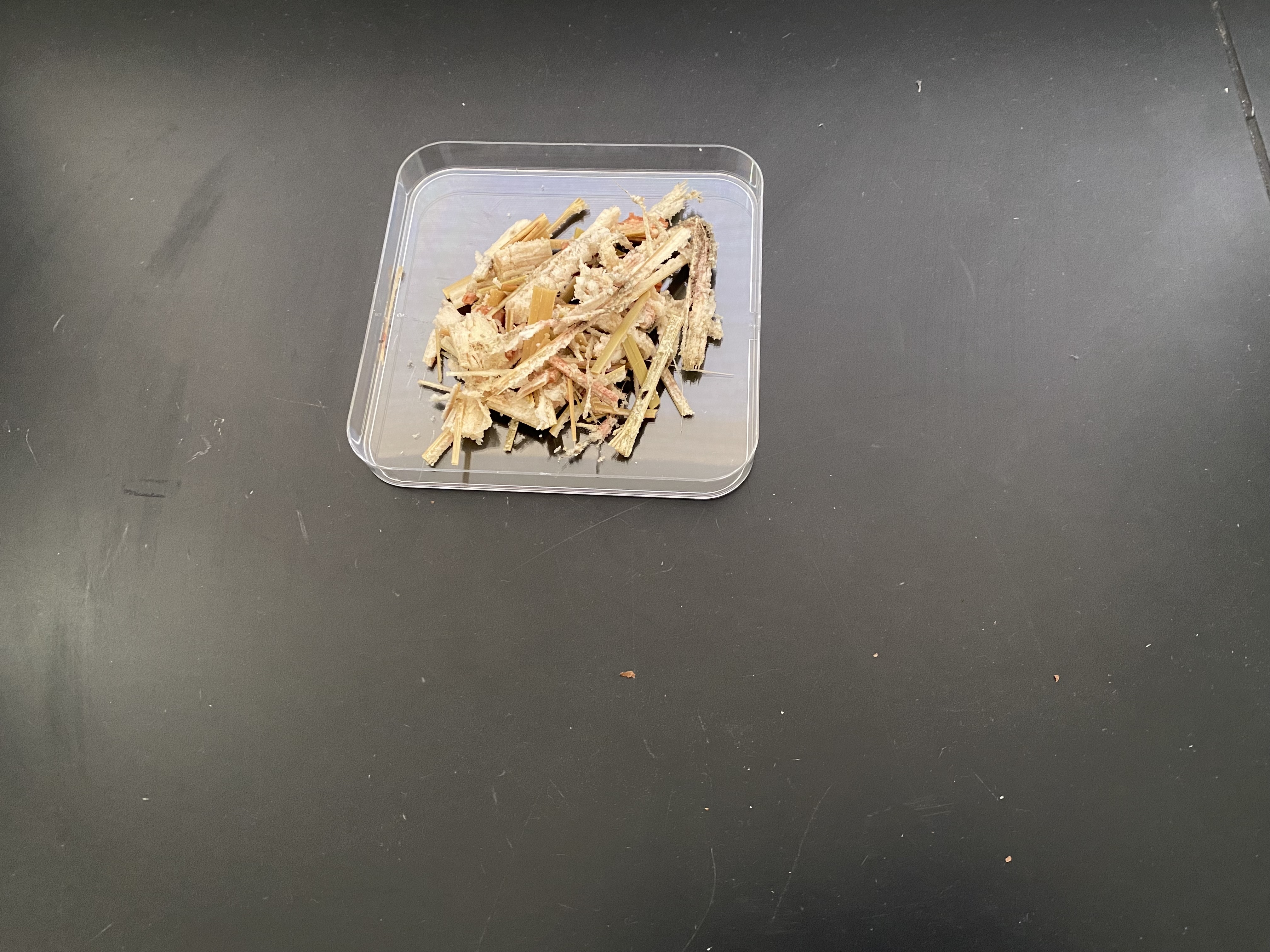

Supplement: Supplementary file 7 — Source data. [file 41564_2024_1799_MOESM7_ESM.zip › Fig4-sourcedata/2023-10-23_sorghumbagasse copy.jpg]

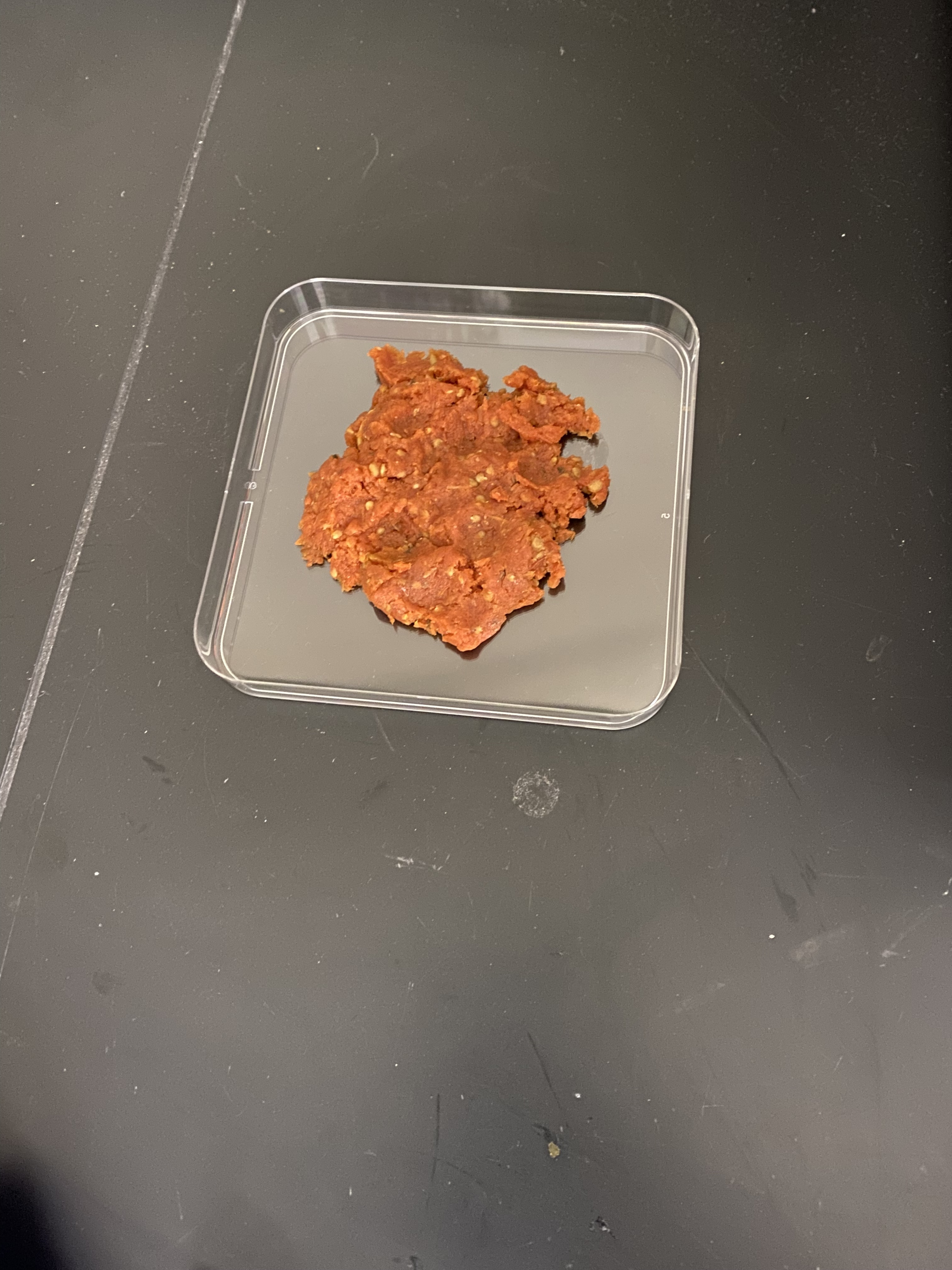

Supplement: Supplementary file 7 — Source data. [file 41564_2024_1799_MOESM7_ESM.zip › Fig4-sourcedata/2023-11-06_tomatopomace copy.jpg]

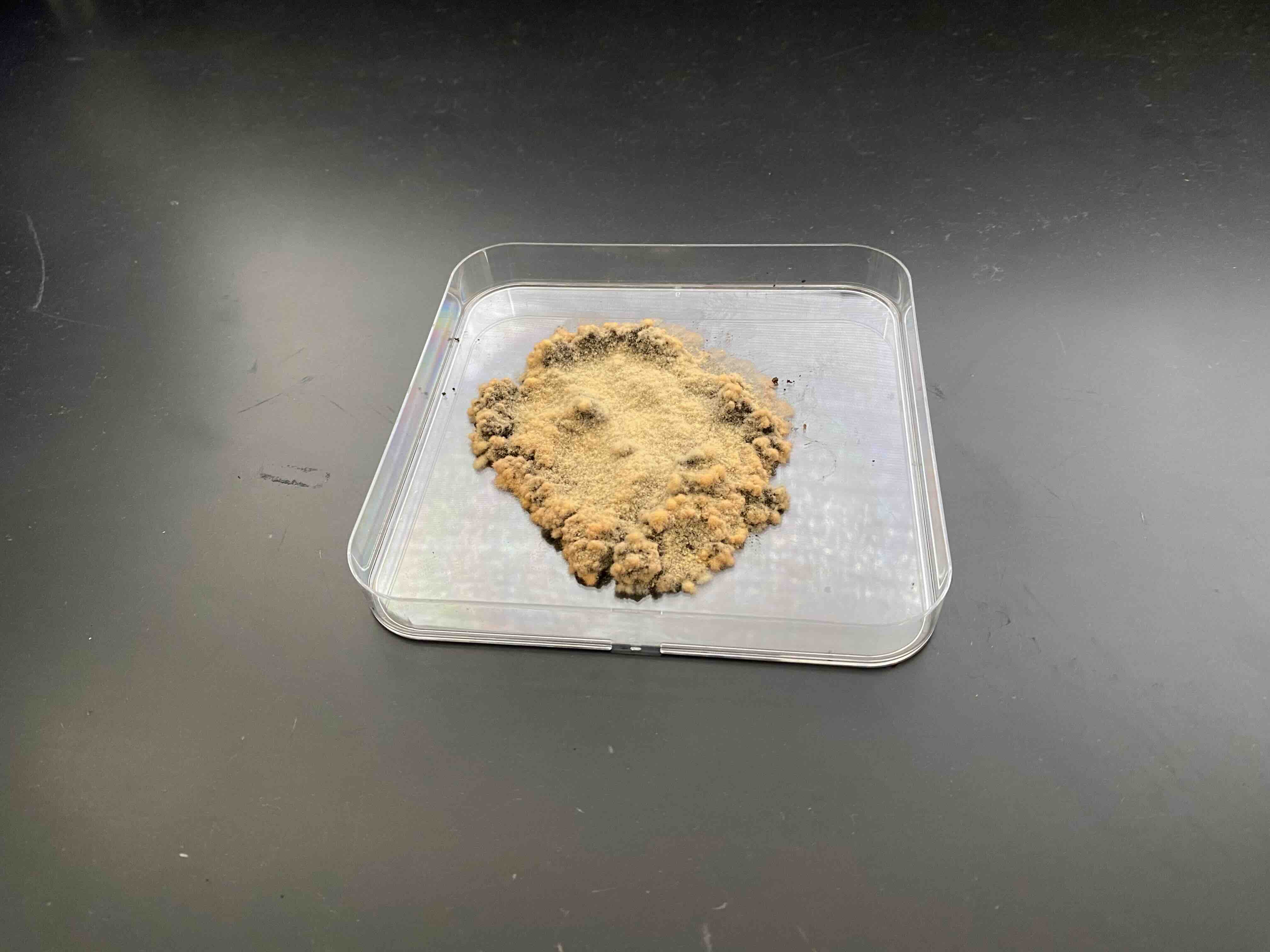

Supplement: Supplementary file 7 — Source data. [file 41564_2024_1799_MOESM7_ESM.zip › Fig4-sourcedata/1_coffegrounds-NI copy.jpg]

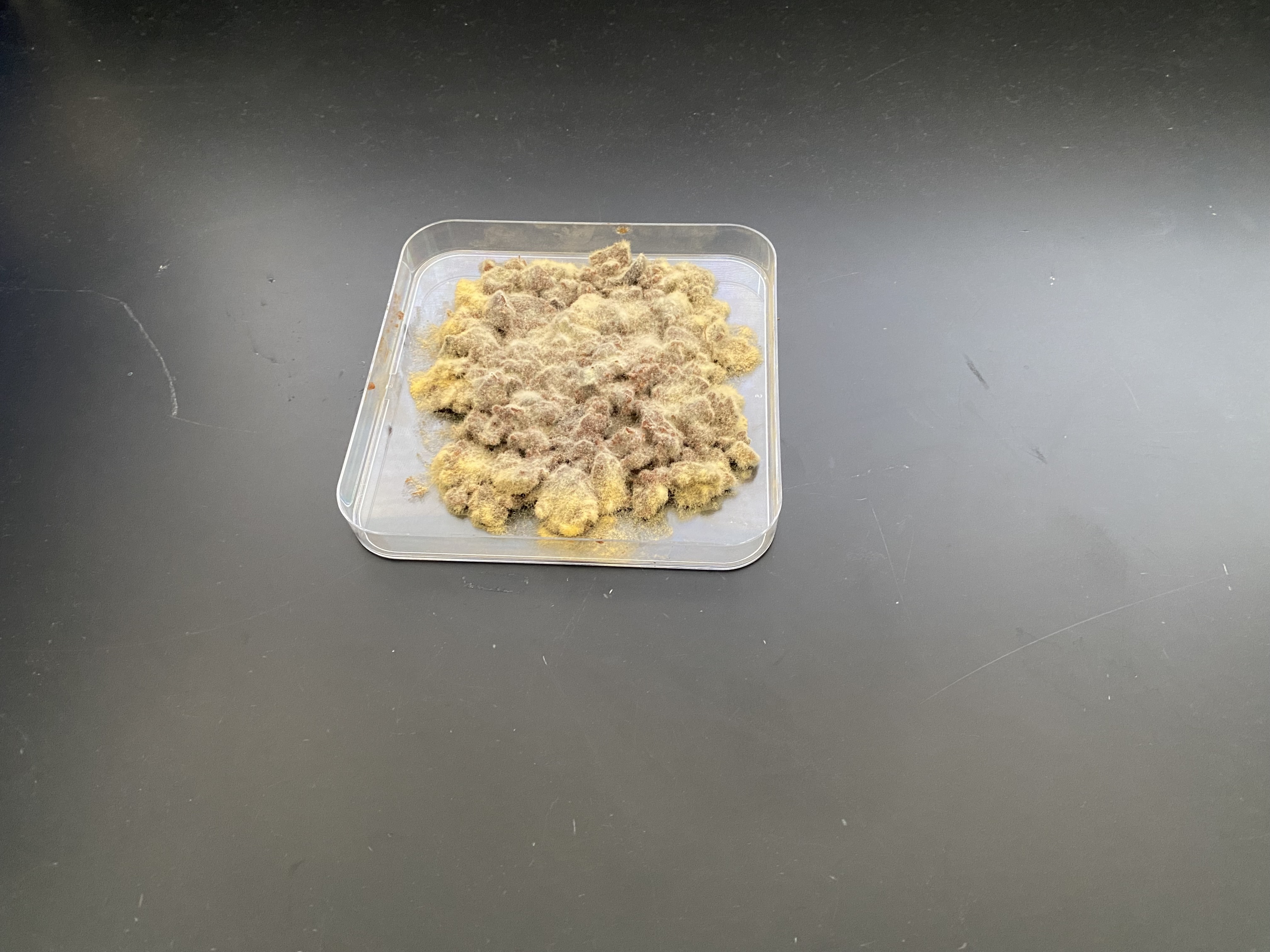

Supplement: Supplementary file 7 — Source data. [file 41564_2024_1799_MOESM7_ESM.zip › Fig4-sourcedata/9_almondhulls-NI copy.jpg]

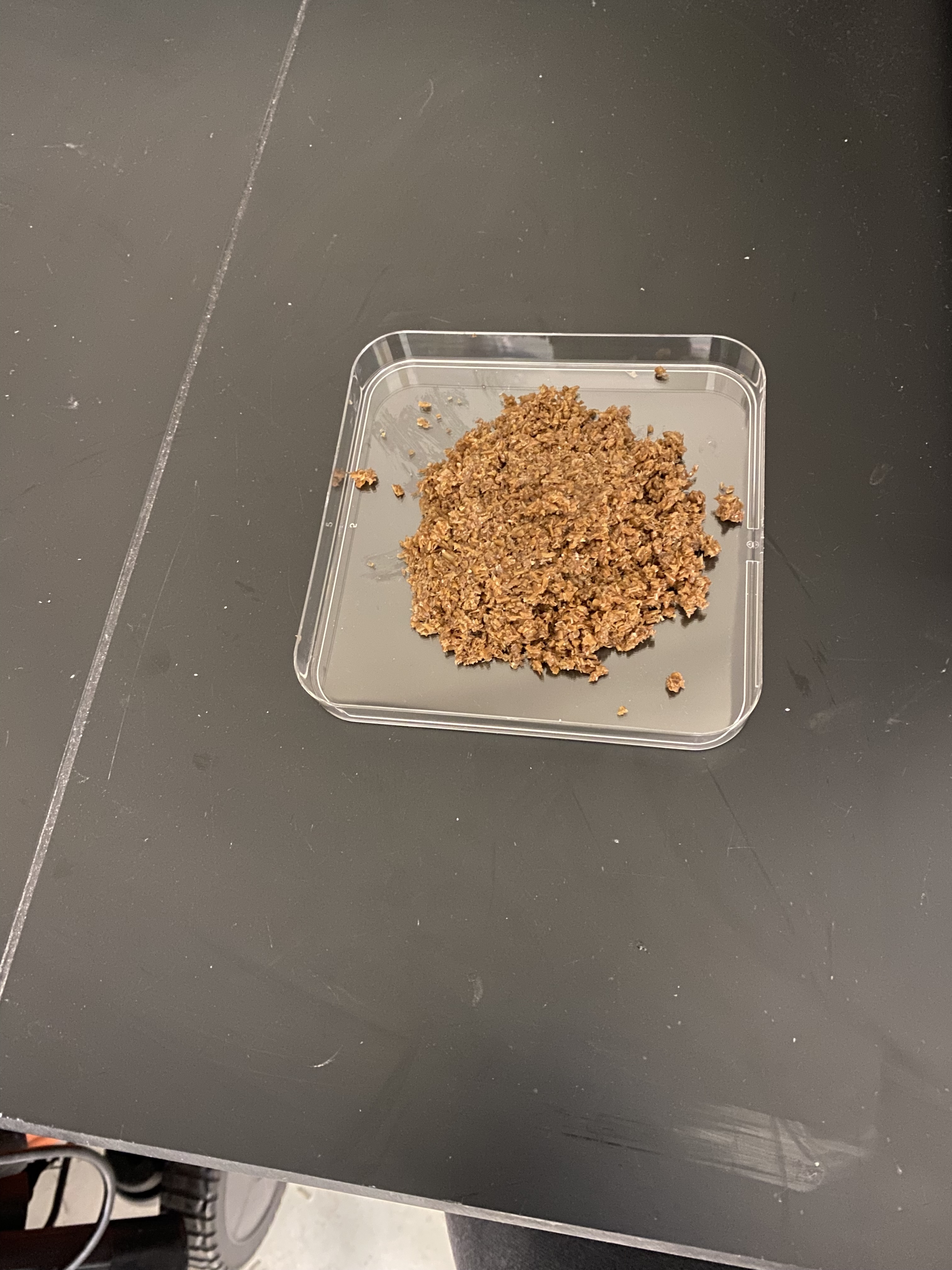

Supplement: Supplementary file 7 — Source data. [file 41564_2024_1799_MOESM7_ESM.zip › Fig4-sourcedata/2023-11-06_wheatbran copy.jpg]

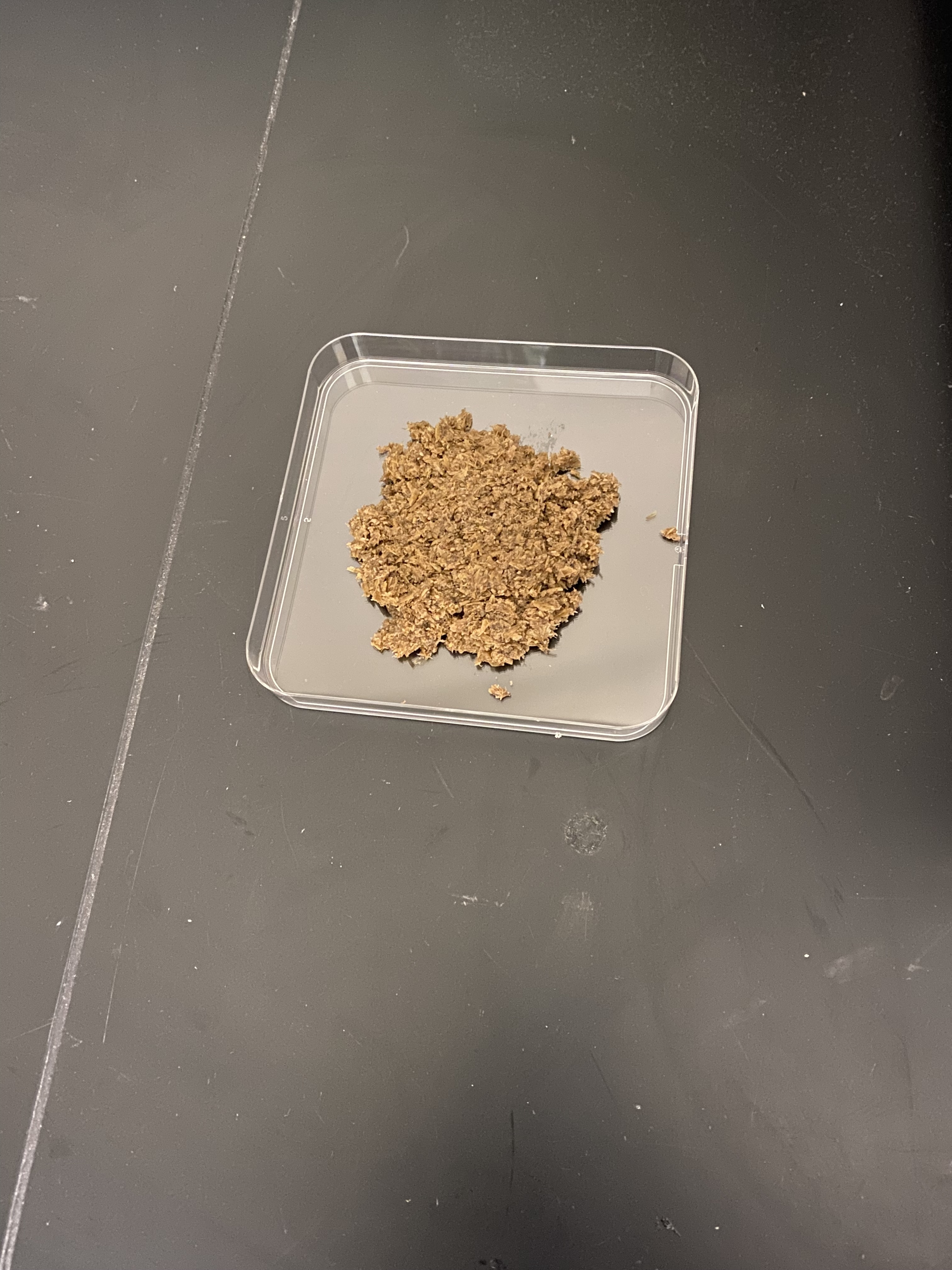

Supplement: Supplementary file 7 — Source data. [file 41564_2024_1799_MOESM7_ESM.zip › Fig4-sourcedata/2023-11-06_oro_barleyhull-husks-NI copy.jpg]

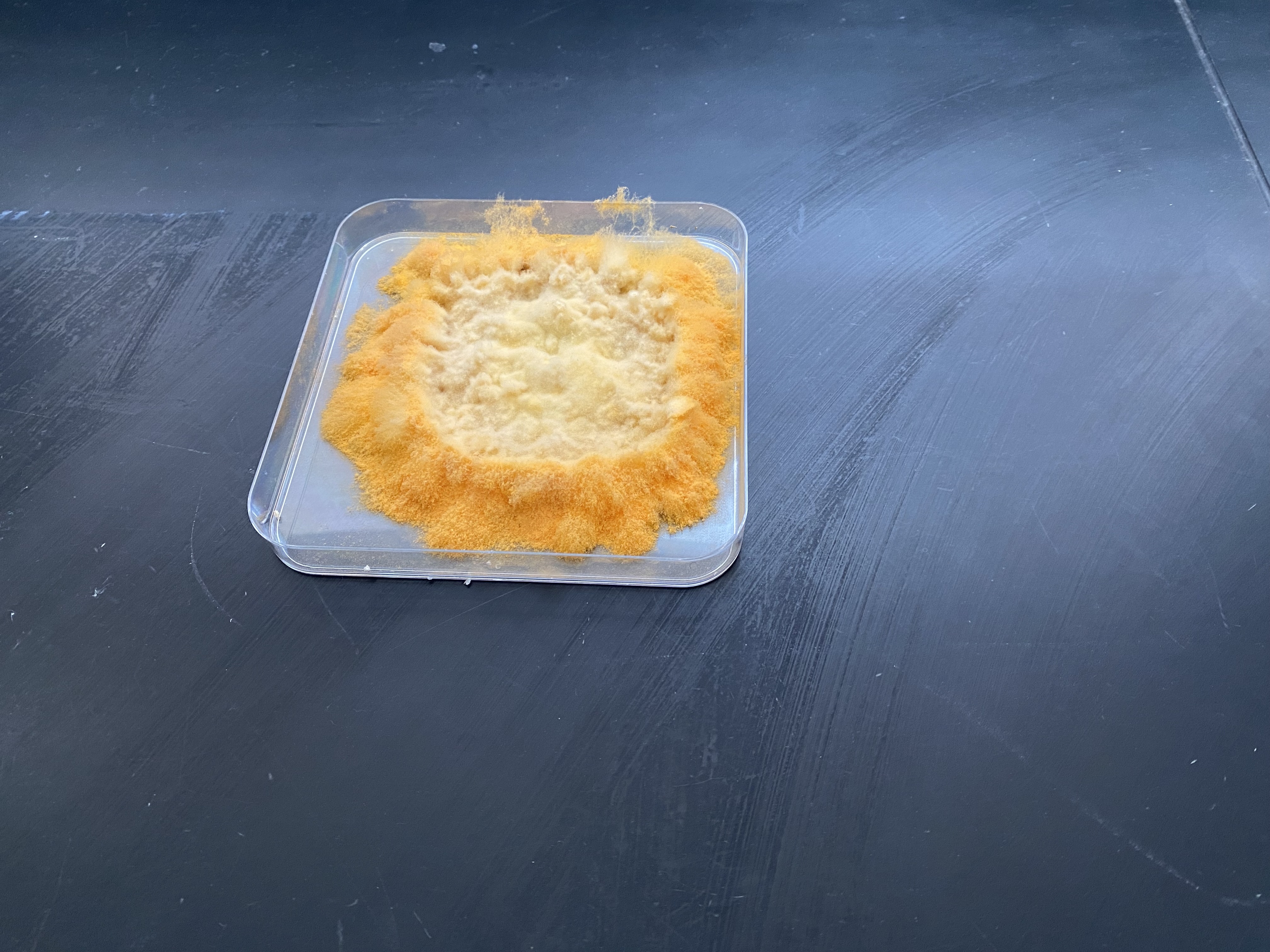

Supplement: Supplementary file 7 — Source data. [file 41564_2024_1799_MOESM7_ESM.zip › Fig4-sourcedata/Okara_2-NI copy.jpg]

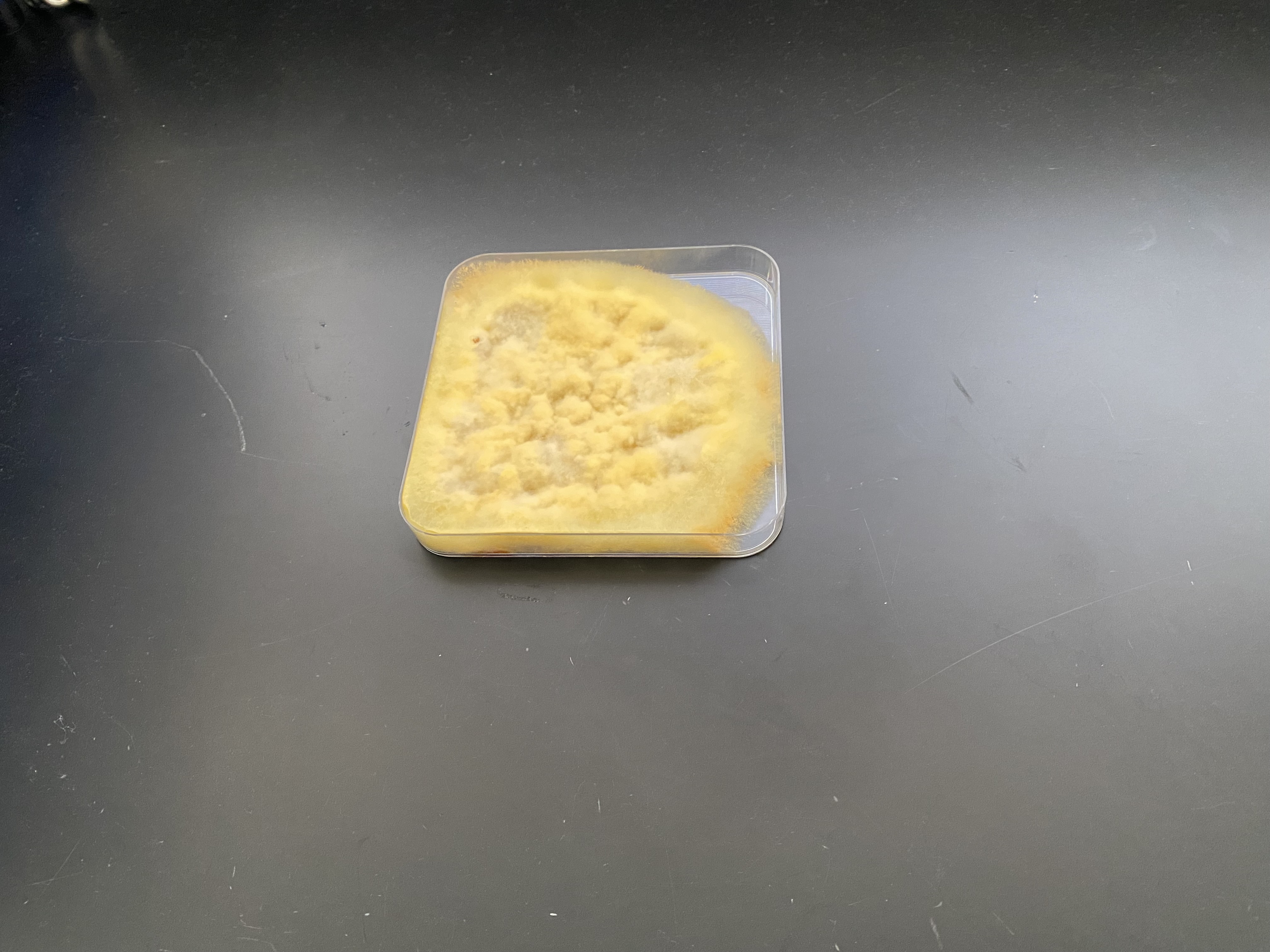

Supplement: Supplementary file 7 — Source data. [file 41564_2024_1799_MOESM7_ESM.zip › Fig4-sourcedata/6_carrotpulp-NI copy.jpg]

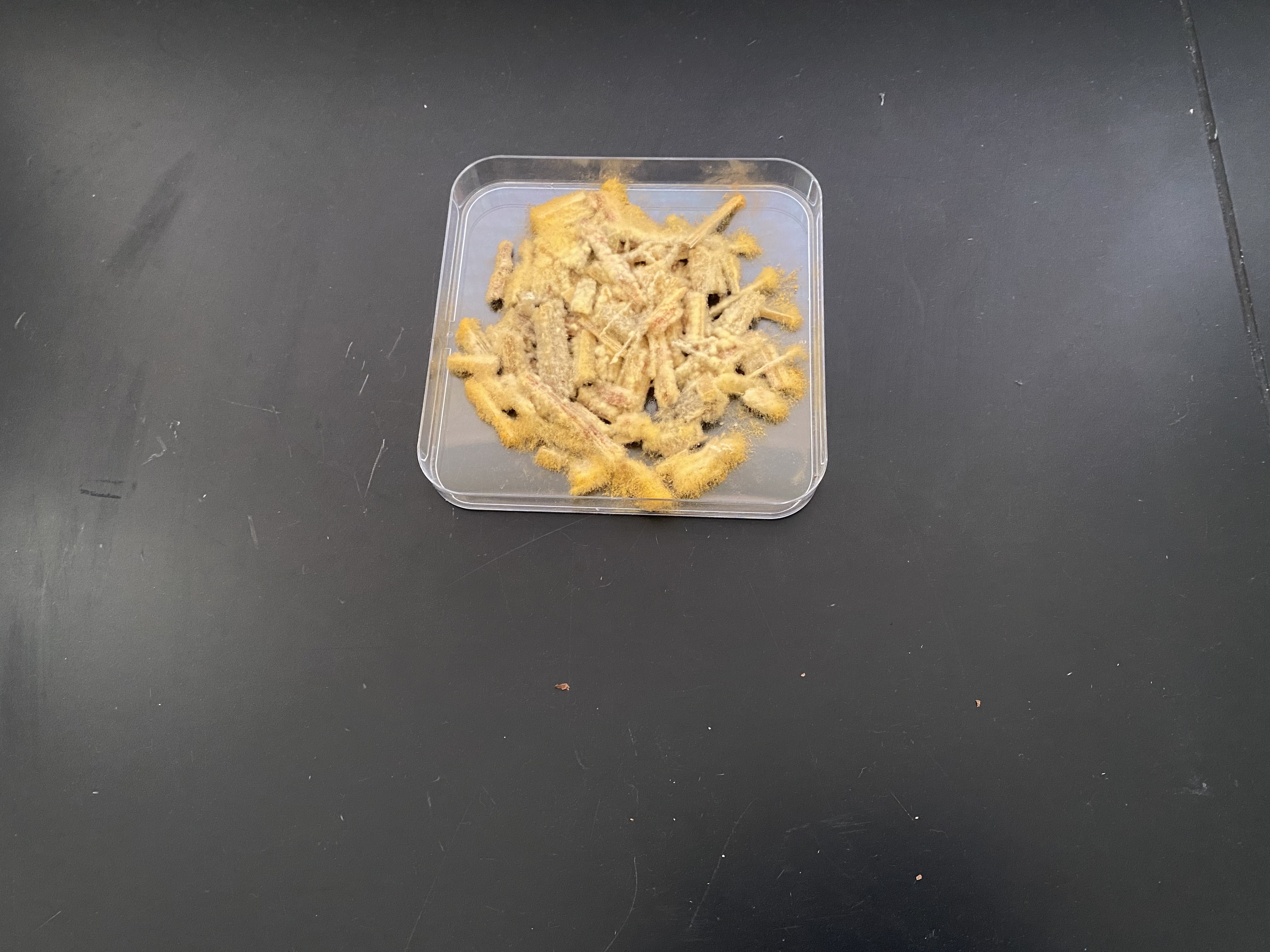

Supplement: Supplementary file 7 — Source data. [file 41564_2024_1799_MOESM7_ESM.zip › Fig4-sourcedata/2023-10-23_sorghumbagasse-NI copy.jpg]

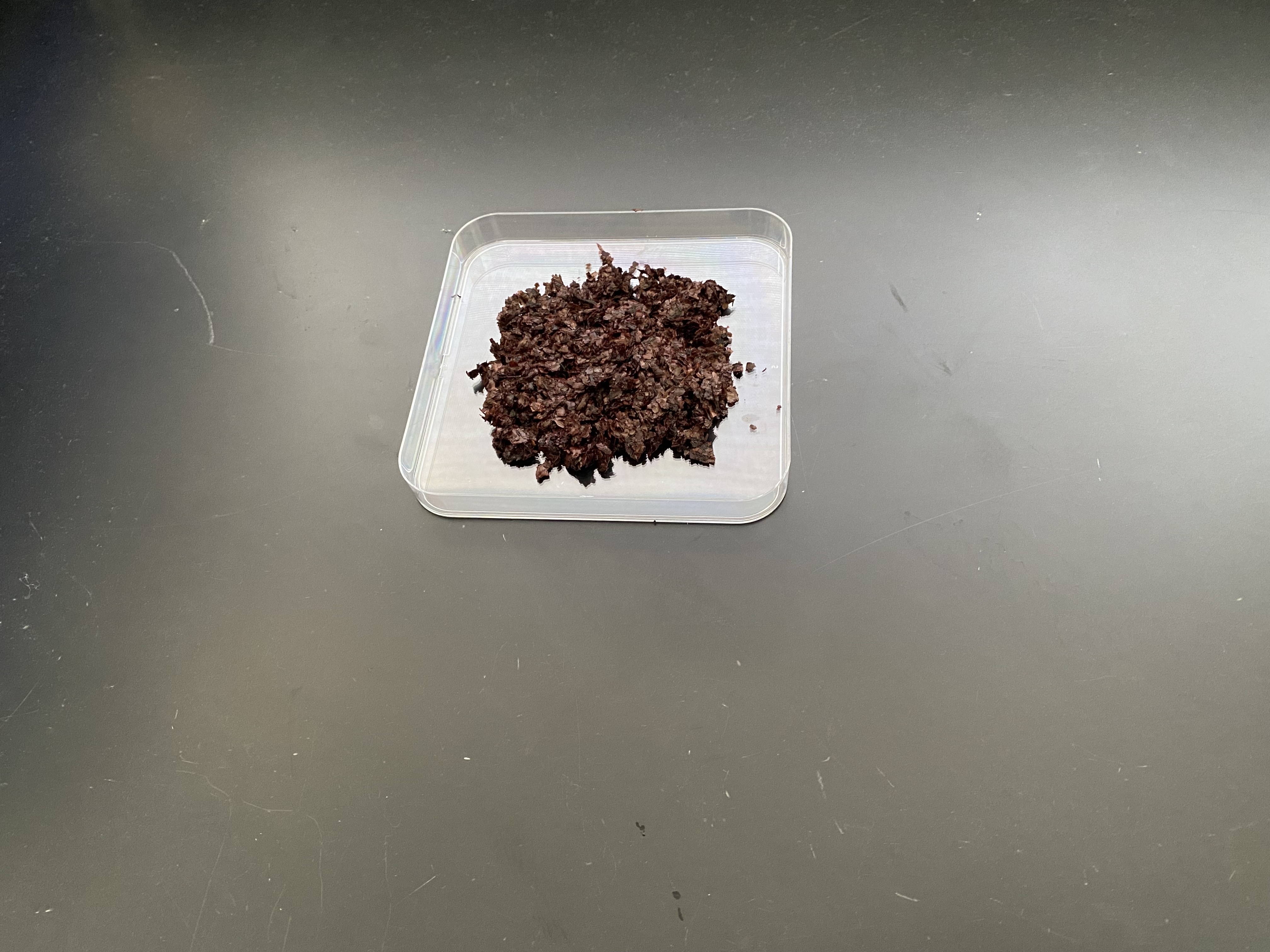

Supplement: Supplementary file 7 — Source data. [file 41564_2024_1799_MOESM7_ESM.zip › Fig4-sourcedata/7_hazelnutskin copy.jpg]

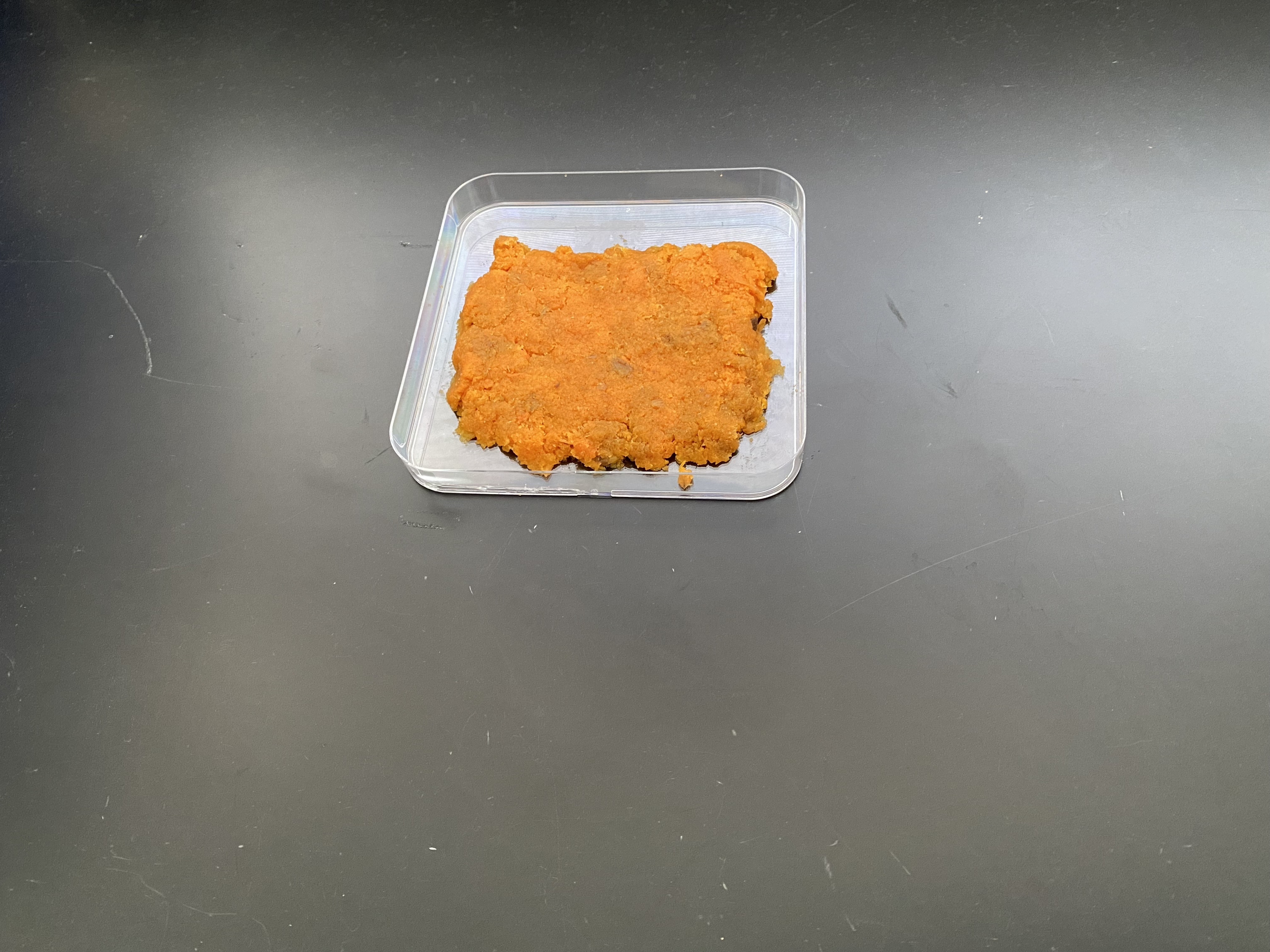

Supplement: Supplementary file 7 — Source data. [file 41564_2024_1799_MOESM7_ESM.zip › Fig4-sourcedata/6_carrotpulp copy.jpg]
